# Design of Heme Enzymes with a Tunable Substrate Binding Pocket Adjacent to an Open Metal Coordination Site

**DOI:** 10.1021/jacs.3c02742

**Published:** 2023-06-21

**Authors:** Indrek Kalvet, Mary Ortmayer, Jingming Zhao, Rebecca Crawshaw, Nathan M. Ennist, Colin Levy, Anindya Roy, Anthony P. Green, David Baker

**Affiliations:** † Institute for Protein Design, University of Washington, 3946 W Stevens Way NE, Seattle, Washington 98195, United States; ‡ Department of Biochemistry, University of Washington, Seattle, Washington 98195, United States; § Manchester Institute of Biotechnology, School of Chemistry, 131 Princess Street, The University of Manchester, Manchester M1 7DN, U.K.; ∥ Howard Hughes Medical Institute, University of Washington, Seattle, Washington 98195, United States

## Abstract

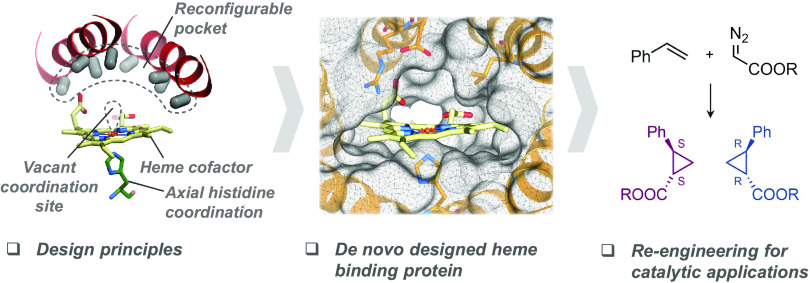

The catalytic versatility of pentacoordinated iron is highlighted by the broad range of natural and engineered activities of heme enzymes such as cytochrome P450s, which position a porphyrin cofactor coordinating a central iron atom below an open substrate binding pocket. This catalytic prowess has inspired efforts to design de novo helical bundle scaffolds that bind porphyrin cofactors. However, such designs lack the large open substrate binding pocket of P450s, and hence, the range of chemical transformations accessible is limited. Here, with the goal of combining the advantages of the P450 catalytic site geometry with the almost unlimited customizability of de novo protein design, we design a high-affinity heme-binding protein, dnHEM1, with an axial histidine ligand, a vacant coordination site for generating reactive intermediates, and a tunable distal pocket for substrate binding. A 1.6 Å X-ray crystal structure of dnHEM1 reveals excellent agreement to the design model with key features programmed as intended. The incorporation of distal pocket substitutions converted dnHEM1 into a proficient peroxidase with a stable neutral ferryl intermediate. In parallel, dnHEM1 was redesigned to generate enantiocomplementary carbene transferases for styrene cyclopropanation (up to 93% isolated yield, 5000 turnovers, 97:3 e.r.) by reconfiguring the distal pocket to accommodate calculated transition state models. Our approach now enables the custom design of enzymes containing cofactors adjacent to binding pockets with an almost unlimited variety of shapes and functionalities.

## Introduction

Heme-binding proteins carry out a wide range of chemical reactions in biology^1−5^ and directed evolution of cytochrome P450s, peroxidases, peroxygenases, globins, and other heme enzymes has led to a wealth of new catalytic activities.^6−18^ The key structural feature that gives rise to this catalytic versatility is a pentacoordinate heme iron cofactor positioned adjacent to an open substrate binding pocket. De novo design efforts have taken advantage of the simplicity and designability of helical bundle scaffolds^19^ to generate porphyrin-containing catalysts.^20−29^ However, this simplicity is also a limitation as such scaffolds cannot support large, open, and customizable active site pockets adjacent to the transition metal. These systems can also display considerable conformational flexibility/heterogeneity making them difficult to structurally characterize or rationally engineer, and covalent attachment of the heme cofactor may be needed to achieve selective catalysis. The design of porphyrin-binding proteins with large central cavities is a more difficult design challenge, as the side chain interactions that determine protein folding and stability generally reside in the protein core.

**Figure 1:**
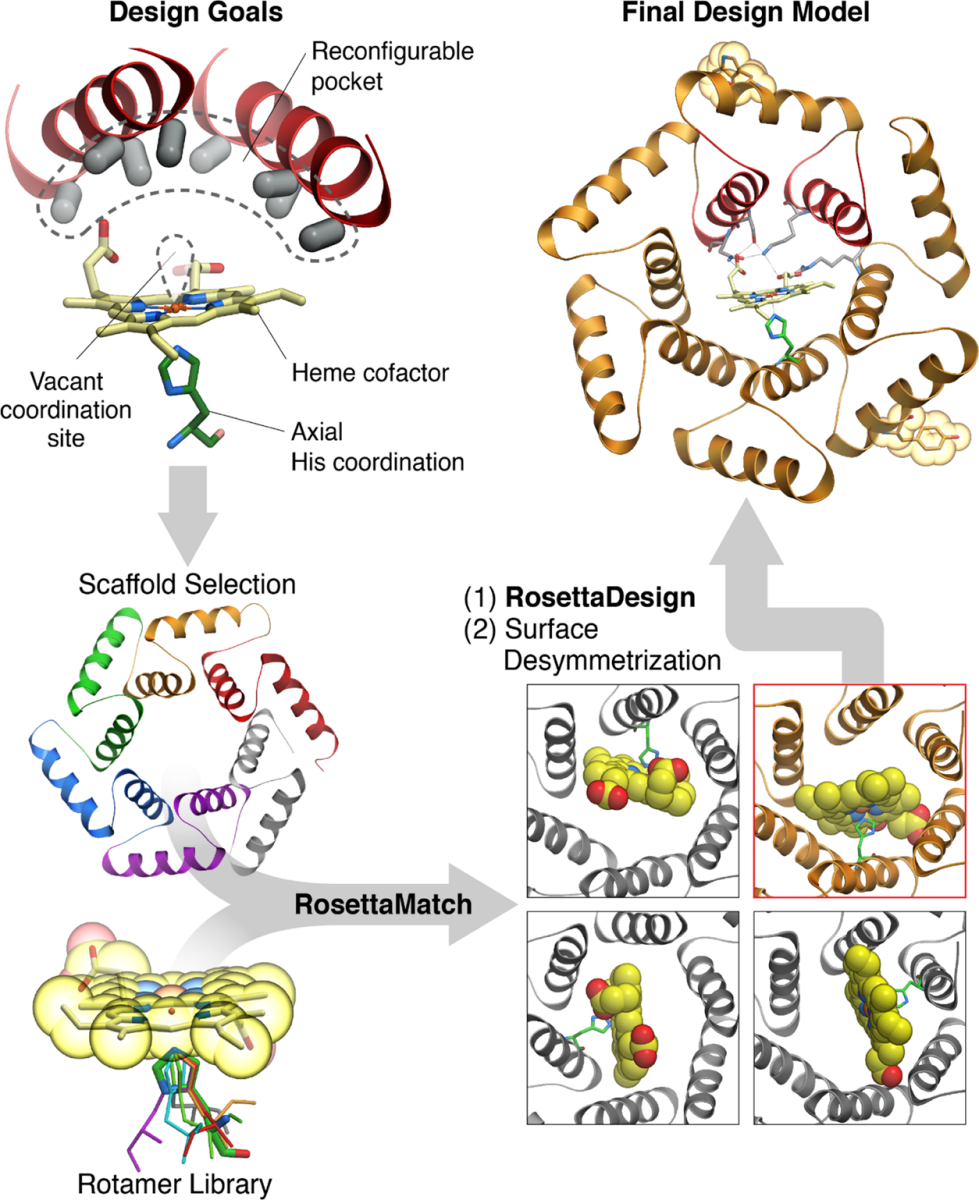
Schematic of the approach to computational design. To generate heme-binding proteins containing a reconfigurable pocket near a free coordination site on the Fe atom (top left), a Heme-HIS moiety was matched into the pore of helical solenoid scaffolds of appropriate size (bottom left and right). The sequence around the heme cofactor was then optimized using Rosetta (top right).

Advances in de novo protein design now enable the design of a wide variety of protein scaffold geometries extending far beyond helical bundles.^30−36^ With this control over structure and stability, protein functional sites can now be designed starting from a specification of an ideal site rather than being limited to particular scaffolds.^37,38^ We reasoned that this control could enable the design of hyperstable porphyrin-binding proteins with large reconfigurable pockets adjacent to an open transition metal coordination site. Such proteins could provide starting points for extending the catalytic potential of porphyrin-bound transition metals beyond natural scaffolds, as the shape of the catalytic pocket and arrangements of functional residues can be more extensively tuned. To achieve this goal, we aimed to design proteins with several key properties. First, a large central cavity lined by many reconfigurable side chains to enable the binding of a wide variety of substrates and tuning of active site geometry and chemistry. Second, very high stability, with the side chain interactions driving folding independent of the central cavity, to enable active site optimization free from the constraints of protein folding and stability. Third, overall backbone structure modularity to enable extensive reconfiguration of the structure lining one part of the active site without disruption of the overall fold to accommodate an even wider range of substrate sizes, shapes, and chemistries (Figure 1).

## Results and Discussion

### Design of a Heme-Binding Protein

We chose closed α helical solenoid repeat architectures as optimal structural solutions to the general porphyrin-based protein catalyst design challenge (Figure 1). These hyperstable proteins have large central cavities lined by side chains emanating from repeating structural units; the stability and structure of the fold are determined by hydrophobic packing interactions within the repeat units, and hence, the cavity lining residues are almost entirely reconfigurable. The protein backbone itself is reconfigurable in repeat proteins as individual repeats can be structurally diversified.^39^ Within the family of closed helical solenoids, we selected a toroidal scaffold (PDB id: 4YXX)^40^ with an interior cavity sufficiently large to position the heme cofactor and an adjacent substrate, but compact enough that side chains at the opposite end of the cavity from the heme can modulate catalysis, substrate binding affinity, and specificity.

We sought to incorporate a heme-binding site within the central cavity of the toroid, with a proximal histidine ligand directly coordinating the iron atom of the cofactor, the distal site open for catalysis, and sufficient non-polar and hydrogen bonding side chain–cofactor interactions at the base and edges of the binding pocket to hold the heme in place without obstructing the free coordination site or interfering with potential substrate binding. We used Rosetta Match^41^ to place a histidine-coordinated heme into the toroid, limiting the matched positions only to those that would place the cofactor into the center of the pore (Figure 1). The heme model contained a methyl group bound to the iron atom, opposite to the histidine, acting as a steric placeholder to ensure free volume for a future substrate. In a subset of calculations, a supporting Glu or Asp side chain hydrogen bonding to the heme-ligating histidine was included to increase active site pre-organization. The remaining amino acid identities and conformations flanking the heme-binding site were optimized using Rosetta combinatorial design calculations^42^ for binding the hemin cofactor and supporting the coordinating histidine. The resulting design models were evaluated based on the His–Fe interaction geometry, shape complementarity, and pre-organization of the heme-binding pocket (assessed by side chain packing calculations in the absence of the heme), and pre-organized side chain hydrogen bonds with the propionate groups of the hemin to enable precise control of heme-binding orientation (Supplementary Figure 30). For designs having these features, ligand docking calculations were carried out using Rosetta GALigandDock,^43^ and those 22 for which the designed heme-binding orientation was lower in energy than any alternative binding modes were selected for experimental characterization (see SI for further details on the selection process). To aid with structure determination, the surface of the protein was desymmetrized by introducing two mutations K29W and K165Y.

### Structural and Spectroscopic Characterization

**Figure 2:**
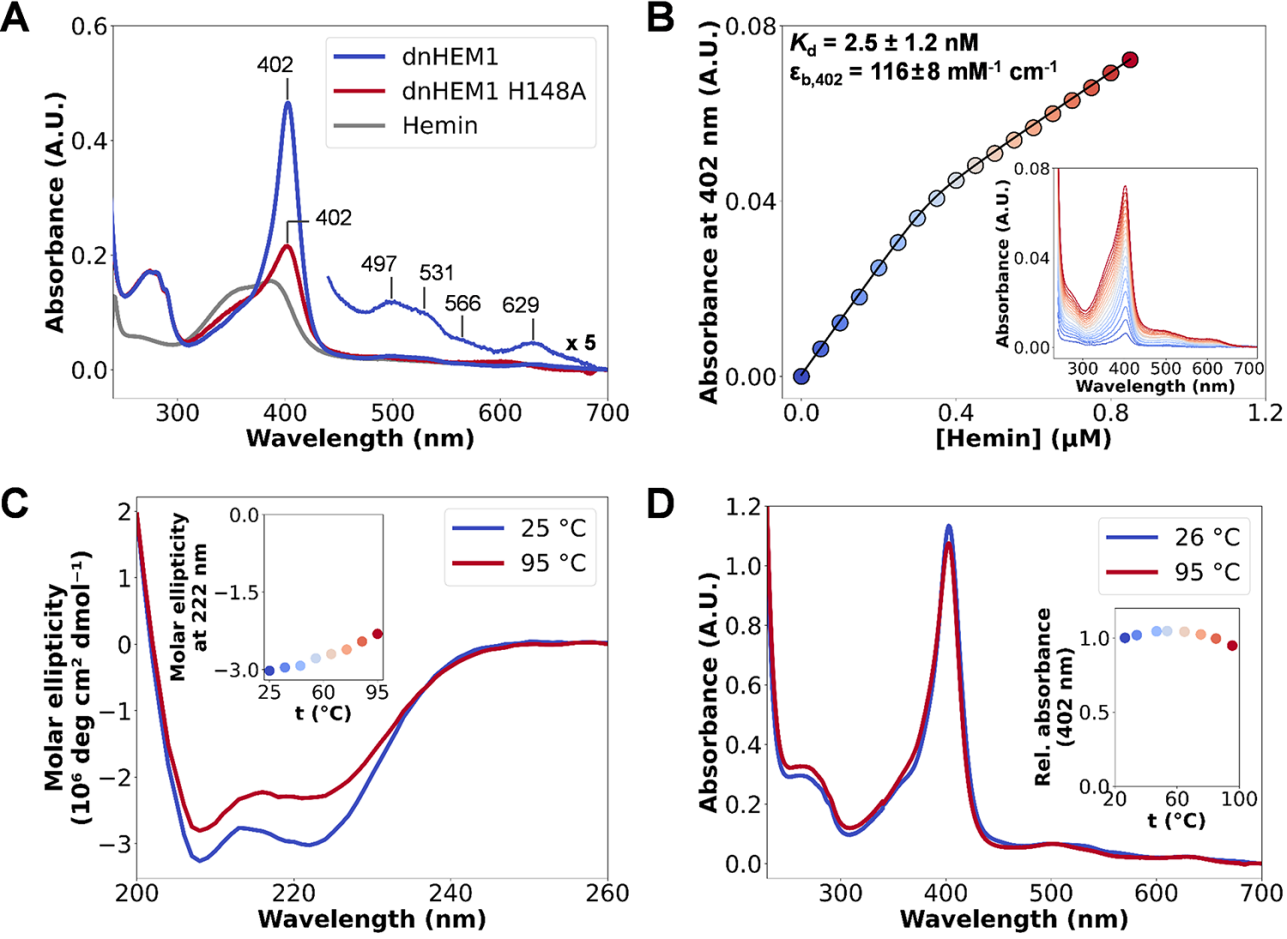
Characterization of Heme binding. (A) UV/Vis spectra of **dnHEM1** (blue) and its H148A mutant (red), after mixing 10 μM protein with 2 μM hemin, indicating the importance of H148 for heme binding. (B) *K*_d_ determination by heme titration into **dnHEM1** (0.4 μM) and following absorbance changes at 402 nm, which were plotted against heme concentration and fitted to a one-site binding equation (see SI). (C) CD spectra of ***holo*****-dnHEM1** at increasing temperatures. (D) UV/Vis spectra of ***holo*****-dnHEM1** collected at increasing temperatures indicate that heme-binding ability is retained at temperatures up to 95 °C.

We selected twenty-two designs for expression in *Escherichia coli* (*E. coli*) and found that fifteen of them were well-expressed, soluble, and had size exclusion chromatography (SEC) elution volumes consistent with the intended monomeric state (Supplementary Figures 1 and 2). To identify potential binders, we performed a qualitative assay by incubating 30 μM of purified *apo*-proteins with 10 μM hemin and directly recording the UV/Vis spectra (Supplementary Figure 3). One of the designs, **dnHEM1**, yielded a promisingly sharp Soret band (λ_Soret_ = 402 nm), together with Q band features at 497, 531, 566, and 629 nm, reminiscent of the spectral features of natural heme proteins (Figure 2A).^44^ The UV/Vis spectrum of the ferrous-CO bound state of **dnHEM1** has a sharp Soret feature at 417 nm, which is also consistent with a His-ligated heme iron (Supplementary Figure 12).^45^ Upon isolating a fully heme-loaded protein, we measured a *ReinheitszahlR*_Z_-value (*A*_Soret_/*A*_280_) of 5.62.^46^ The dissociation constant (*K*_d_) of this design for hemin was determined to be <10 nM by titrating hemin into a solution of the *apo*-protein and monitoring absorbance changes at 402 nm (Figure 2B); three independent titrations yielded an average *K*_d_ of 2.5 ± 1.2 nM (Supplementary Figure 10). Replacement of the putative heme-coordinating His148 ligand of **dnHEM1** by Phe or Ala leads to a substantial broadening of the Soret feature of proteins loaded with heme in vitro (Figure 2A and Supplementary Figure 5). **dnHEM1** is a hyperthermostable protein (*T*_m_ > 95 °C), as evidenced by minimal changes in the circular dichroism (CD) spectrum in the 25–95 °C temperature range (Figure 2C). The structural integrity of the protein is not dependent on heme binding, with no differences in CD *T*_m_ measurements observed between *apo* and *holo* variants (Supplementary Figure 7). UV/Vis spectra collected at increasing temperatures showed minimal changes in the Soret band intensity and wavelength, suggesting that **dnHEM1** retains most of its heme-binding ability even at 95 °C (Figure 2D). Similarly, minimal spectral changes are observed across a wide range of pH values (Supplementary Figure 13). Changing the surface charge of **dnHEM1** from positive (pI 10) to slightly negative (pI 6) through 12 mutations, bringing it closer to most naturally occurring proteins, also had no detrimental effect on its expression level, solubility, or heme-binding ability (as judged by the sharpness and λ_max_ value of the Soret band; see Supplementary Figure 8).

**Figure 3:**
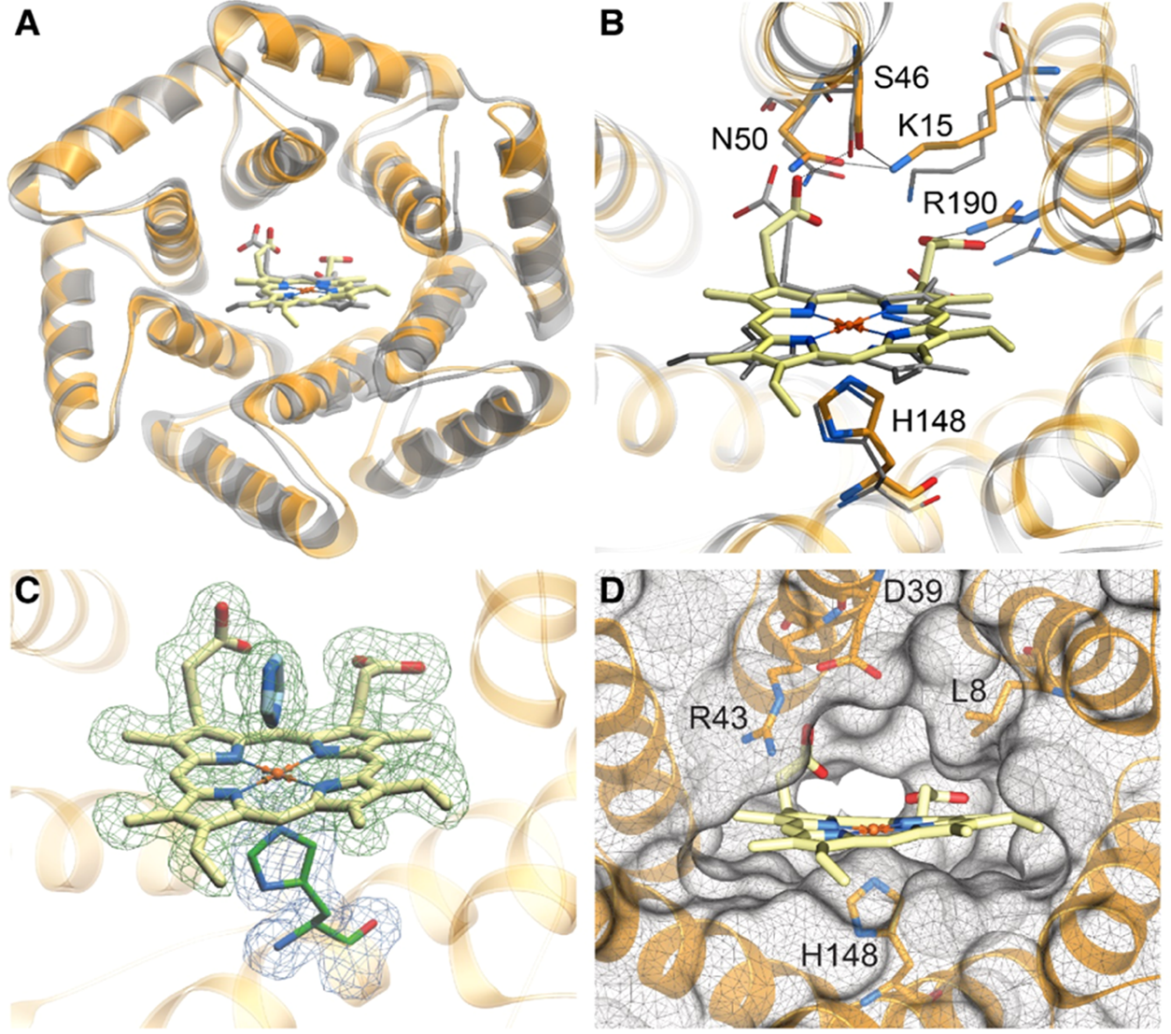
The crystal structure of **dnHEM1** (PDB id: 8C3W) closely matches the design model. (A) Crystal structure of **dnHEM1** (orange) overlaid with the design model (silver). (B) Superposition of the heme-binding site of the **dnHEM1** design model (silver) and the crystal structure (orange). Hydrogen bond interactions with heme are indicated with dashed lines. (C) Crystal structure of **dnHEM1** showing the electron density corresponding to heme and the exogenous imidazole in blue (*F*o – *F*c omit map contoured at 1σ) and His148 shown in green (2*F*o – *F*c map contoured at 1σ). (D) Open substrate binding pocket at the distal site upon omission of co-crystallized imidazole.

The crystal structure of heme-loaded **dnHEM1** was solved to 1.6 Å resolution (Figure 3A, PDB id: 8C3W) and closely matches the computational design model (1.18 Å backbone RMSD). The heme cofactor is positioned as designed but adopts a flipped conformation, which alters the relative positioning of the heme vinyl and methyl substituents, but preserves the positioning of the heme propionates. Similar flipped heme orientations have previously been observed in natural and engineered heme proteins.^44,47,48^ The propionate groups interact with a network of polar residues (K15, S46, N50, R190) (Figure 3B). The heme iron is coordinated by His148 as designed, which in turn forms a hydrogen bond with the backbone carbonyl oxygen of Ile144. The open coordination site is occupied by an exogenous imidazole in the **dnHEM1** crystal structure (Figure 3C). Overall, the crystal structure shows that we were successful in achieving our design goals; the heme is held in place by numerous polar and hydrophobic side chain–cofactor interactions, and the heme iron is coordinated on one side by a designed histidine and, on the other, by a small molecule from a solution that occupies a central binding cavity (Figure 3C,D).

### Engineering Peroxidase Activity

**Figure 4:**
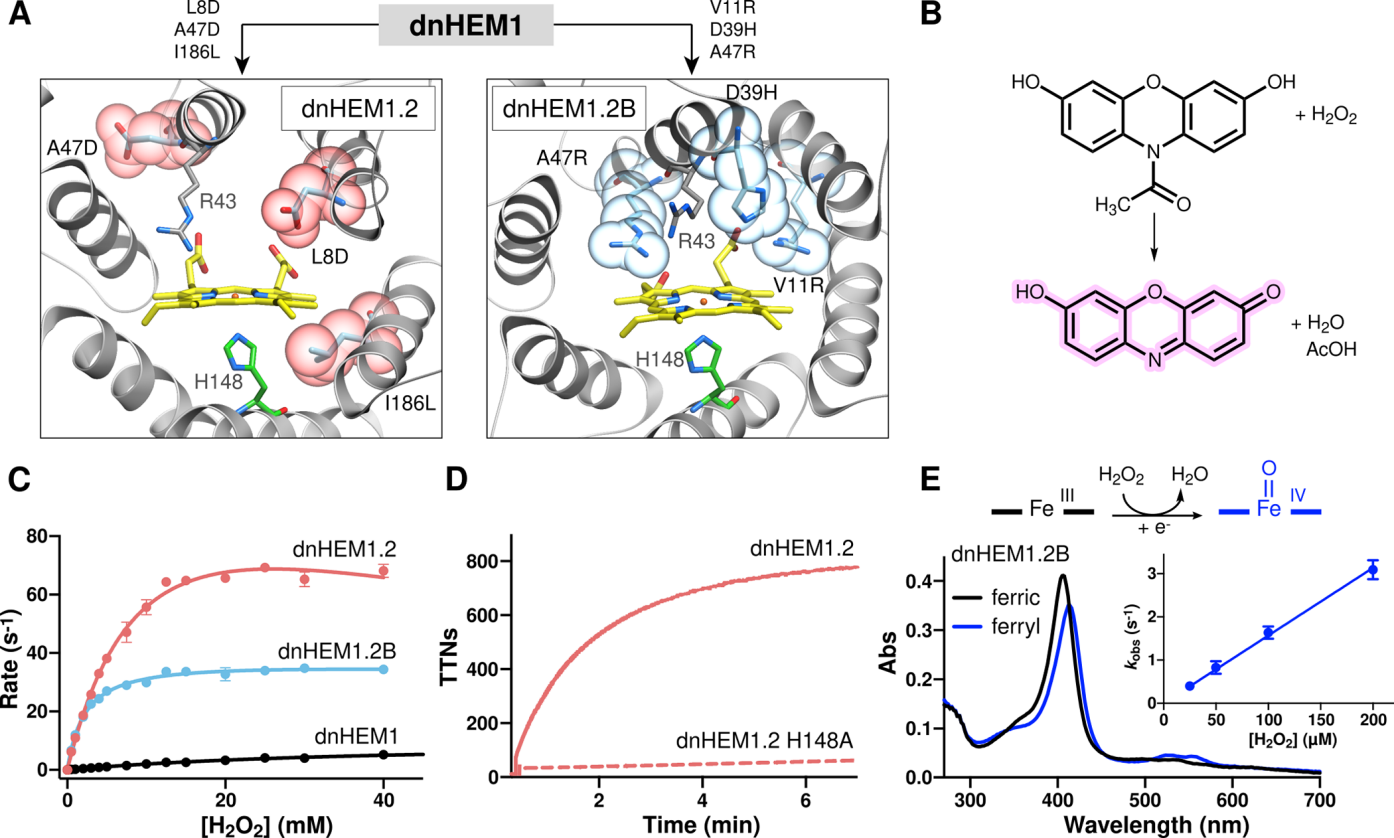
Directed evolution converts **dnHEM1** into a proficient peroxidase with a stable high valent ferryl intermediate. (A) AlphaFold2^52^ predicted models of **dnHEM1.2** and **dnHEM1.2B** indicating the mutations accumulated as a result of divergent directed evolution. Sites of mutation for **dnHEM1.2** and **dnHEM1.2B** are shown as atom colored ball and sticks with purple or blue carbons, respectively. The heme cofactor is shown as gray atom colored ball and sticks and CPK spheres. (B) Chemical scheme showing the conversion of Amplex Red dye to resorufin mediated by hydrogen peroxide and **dnHEM1** variants. (C) Michaelis–Menten plots under saturating concentrations of Amplex Red for **dnHEM1** (black, *k*_cat_ = 9.5 ± 0.2 s^–1^, *K*_M[H_2_O_2_]_ = 36.7 ± 1.6 mM), **dnHEM1.2** (red, *k*_cat_ = 129.5 ± 8.7 s^–1^, *K*_M[H_2_O_2_]_ = 11.5 ± 1.2 mM, *K*_I_ = 58.9 ± 10.7 mM) and **dnHEM1.2B** (blue, *k*_cat_ = 37.0 ± 0.3 s^–1^, *K*_M[H_2_O_2_]_ = 2.0 ± 0.1 mM_)_. Error bars represent SD *n* = 3. (D) Time course of resorufin formation by **dnHEM1.2** (red solid line, TTNs = 788 ± 18) and **dnHEM1.2** H148A (red dotted line). (E) Formation of a ferryl intermediate (blue) in **dnHEM1.2B** upon oxidation of the ferric enzyme (black line) with H_2_O_2_. The observed neutral ferryl heme state is most likely formed via rapid single electron transfer to a transient porphyrin-π cation radical species, with a redox active amino acid side chain being the most likely electron donor. *Inset*: A linear fit of *k*_obs_ vs [H_2_O_2_] was used to derive a bimolecular rate constant of (1.6 ± 0.05) × 10^4^ M^–1^ s^–1^ for ferryl heme formation. Error bars represent SD *n* = 3.

As a first step in exploring the catalytic potential of our designed heme protein, we assessed the peroxidase activity of **dnHEM1** using Amplex Red as a model substrate (Figure 4B).^49^ Arg43 in the distal pocket of **dnHEM1** sits in a similar position, relative to the heme cofactor, to a conserved catalytic arginine found in natural peroxidases. We found that under saturating concentrations of Amplex Red, **dnHEM1** catalyzed the peroxidase reaction with a *k*_cat_ of 9.5 ± 0.2 s^–1^ and *K*_M_ of 36.7 ± 1.6 mM for H_2_O_2_ (Figure 4C). To increase activity, we performed two rounds of directed evolution, targeting residues lining the heme-binding cavity, which were individually randomized using NNK degenerate codons. Beneficial mutations identified during each round were subsequently combined by DNA shuffling (Supplementary Figure 14). Following evolution, we arrived at two triple mutants (**dnHEM1.2**: L8D-A47D-I186L and **dnHEM1.2B**: V11R-D39H-A47R) with significantly improved peroxidase activity (Figure 4A). There are minimal changes to the CD spectra and thermostability in either variant (Supplementary Figures 15 and 16), and in both cases, the catalytic function is strongly dependent on the proximal His148 ligand (Figure 4D and Supplementary Figure 17). These observations suggest that both protein structure and heme-binding geometry are well conserved in these evolved enzymes. The most active variant (**dnHEM1.2**) has two additional carboxylate groups situated in the distal heme pocket and catalyzes the oxidation of Amplex Red with a *k*_cat_ of 129.5 ± 8.7 s^–1^ and *K*_M[H_2_O_2_]_ of 11.5 ± 1.2 mM. This *k*_cat_ value is ca. 20-fold and 5-fold lower than the rate of Amplex Red oxidation reported for HRP and the heavily engineered myoglobin-based peroxidase MbQ2.1 NMH, respectively, but is ca. 200-fold faster than Amplex Red oxidation by APX (see Table S8 of reference (44)).^44^ In contrast, improvements in catalytic efficiency observed in **dnHEM1.2B** (*k*_cat_ of 37.0 ± 0.3 s^–1^ and *K*_M[H_2_O_2_]_ of 2.0 ± 0.1 mM) are achieved by embedding two (positively charged) arginines and a His39 residue into the distal cavity (Supplementary Figure 18). Improvements in catalytic efficiency coincided with stabilization of a neutral ferryl [Fe(IV) = O] heme state (λ_Soret_ = 414 nm and optical features at 524/553 nm) akin to Compound II of natural peroxidases, which can be readily generated in this variant upon mixing of the ferric enzyme with hydrogen peroxide (Figure 4E, rate constants of (1.6 ± 0.05) × 10^4^ M^–1^ s^–1^ and 0.03 ± 0.005 s^–1^ for ferryl intermediate formation and decay, respectively, see Supplementary Figures 19 and 21). For comparison, the Compound II state of ascorbate peroxidase has a Soret maximum at 418 nm and Q bands at 528 and 559 nm. This stabilization of the ferryl state could plausibly arise from additional hydrogen bonding interactions with the ferryl oxygen as a result of distal pocket mutations introduced during evolution.^50,51^ Analysis of the **dnHEM1.2B** evolutionary trajectory reveals a single V11R mutation installed in the first round of evolution was sufficient to allow accumulation of the neutral ferryl heme state, with the additional D39H and A47R mutations increasing the rate of ferryl formation (*k*_obs_ of 1.2 and 3.0 s^–1^ for **dnHEM1** V11R and **dnHEM1.2B**, respectively, using 200 μM hydrogen peroxide, Supplementary Figures 20 and 21). These combined data highlight the potential for modulating the structures and reactivities of key metal-oxo intermediates within our dnHEM scaffolds.

### Computational Design of Enantiocomplementary Carbene Transferases

**Figure 5:**
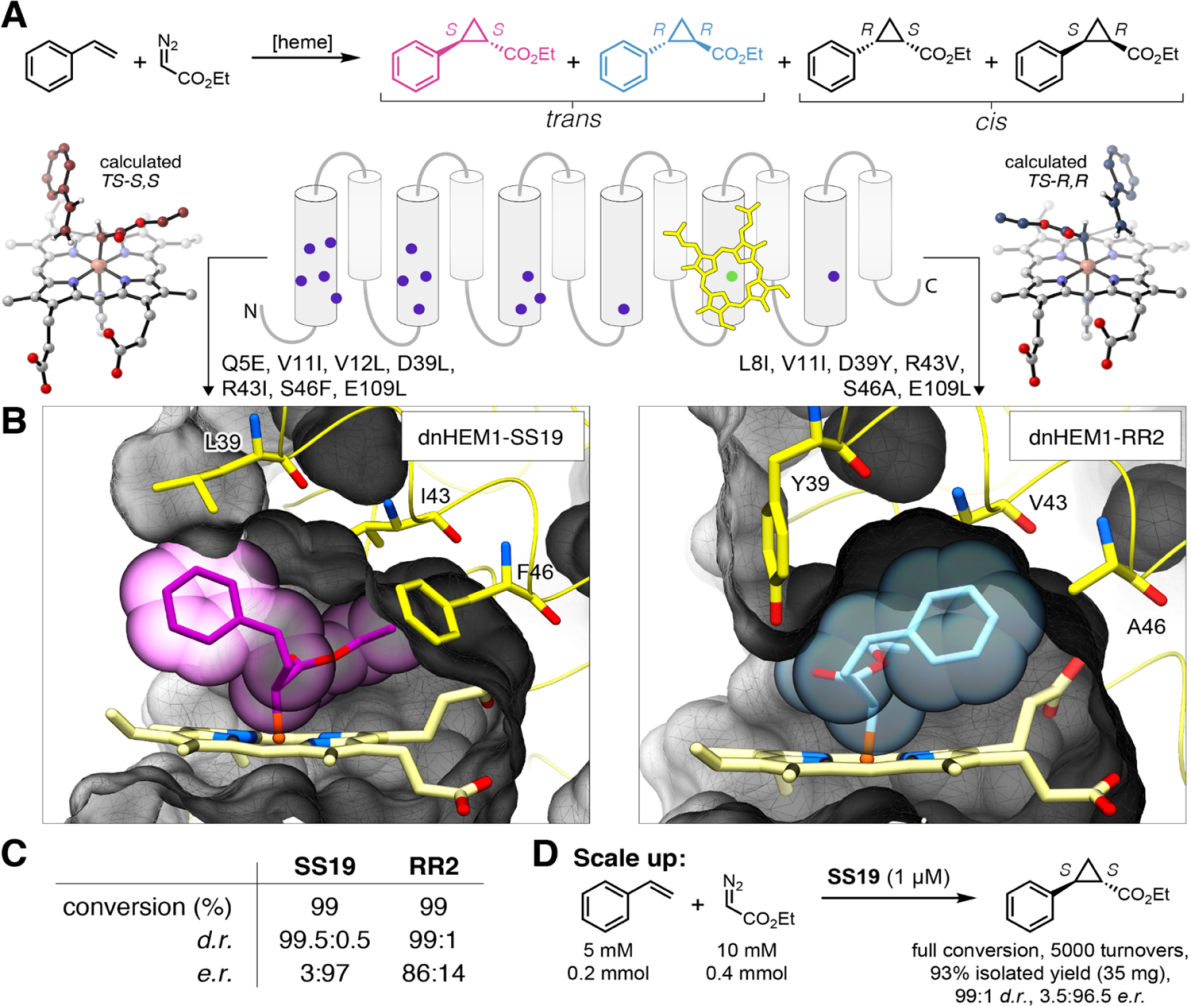
Computational redesign of **dnHEM1** for enantioselective olefin cyclopropanation activity. (A) Enantiocomplementary transition states for *R*,*R*-and *S*,*S*-cyclopropane formation were computed with DFT and selected positions in the distal pocket were redesigned with Rosetta. (B) Complementarity of the designed pocket with most selective enzymes for *S*,*S* (left) and *R*,*R* (right) transition state models. (C) Selectivities obtained with best variants for the synthesis of *S*,*S* and *R*,*R* cyclopropanes. Standard reaction condition: 1 μM catalyst, 1 mM styrene, 10 mM EDA, 100 μM dithionite, under N_2_ in aqueous potassium phosphate buffer (50 mM, NaCl 200 mM, pH 7.2) and 5% MeCN cosolvent for 2 h at 25 °C. See SI for the results with other designs. (D) Preparative scale synthesis of the *S*,*S* stereoisomer, catalyzed by **dnHEM1-SS19** over 2 h at r.t.

We next sought to computationally design new catalytic activities into **dnHEM1**, choosing as a model reaction the cyclopropanation of olefins, in particular, the reaction of ethyl diazoacetate (EDA) and styrene (Figure 5A). This reaction creates two new stereocenters, providing a stringent test of our control over reaction selectivity, and provides access to building blocks useful for the synthesis of bioactive molecules.^53^ The reaction pathways leading to the *S*,*S* and *R*,*R* enantiomers were modeled using density functional theory (DFT) calculations ((Supplementary Figure 32); as proof of principle, we considered only the energetically more facile *trans*-addition of styrene to the Fe-carbenoid intermediate). For each enantiomer, five TS conformers based on the rotation around the Fe-C(COOEt) bond were located and found to be within Δ*G*^‡^ = 3.6 kcal/mol (Supplementary Table 8). We overlaid these transition state models with the heme cofactor of **dnHEM1** and then optimized the sequence in the immediate vicinity of the reaction substrates using Rosetta FastDesign^54^ (a total of 14 residues in the distal pocket were targeted for *in silico* engineering, Figure 5B, Supplementary Figure 33). There was very little overlap between the pocket sequences designed for each of the two enantiomers. We used the metrics described above to select designs for experimental testing (His–Fe constraint score, shape complementarity, pocket stability). This yielded 35 designs that expressed well in *E. coli*, in addition to being soluble and monomeric.

We then evaluated the isolated heme-loaded proteins for cyclopropanation activity with styrene and EDA as the coupling partners in the presence of Na_2_S_2_O_4_ as a reductant. All designs had high diastereoselectivity (d.r. > 95:5) in favor of the *trans*-product, which contrasts the modest selectivity (d.r. 76:24) achieved with free hemin in solution (Supplementary Figure 23 and Table 4). Furthermore, all proteins designed to mediate *S*,*S*-selective catalysis did so, with 2 designs giving enantiomeric ratios (e.r.) > 95:5. Designing *R*,*R*-selective catalysts proved to be more challenging. Nevertheless, we were able to generate *R*,*R*-selective designs with up to 86:14 e.r., highlighting how with computational design it is possible to create substrate binding pockets that are able to discriminate between the two enantiomeric transition states. For comparison, neither the parent design **dnHEM1** nor the evolved peroxidases (**dnHEM1.2** and **dnHEM1.2B**) gave any appreciable levels of enantiocontrol for olefin cyclopropanation (Supplementary Figure 24). Redesign of the **dnHEM1** catalytic pocket for cyclopropanation activity through 6–7 mutations, of which four were from polar to apolar amino acids, had no detrimental effect on protein stability, with the most selective *S*,*S*- and *R*,*R*-designs (**dnHEM1-SS19** and **dnHEM1-RR2)** retaining their secondary structures and heme-binding abilities at 95 °C (Supplementary Figures 25 and 26). To demonstrate synthetic utility, we performed a preparative scale cyclopropanation using **dnHEM1-SS19** as a biocatalyst. Using only 0.02 mol% enzyme, complete conversion of styrene (5 mM) was achieved within 2 h, affording optically enriched *S*,*S*-product (3.5:96.5 e.r., 35 mg) as a single diastereomer in 93% isolated yield (Figure 5D and Supplementary Figure 27). The high reaction yields, total turnover numbers, and stereocontrol achievable with **dnHEM1-SS19** is particularly impressive given that this enzyme was designed in silico and has not been subjected to any evolutionary optimization. By way of comparison, the turnover numbers and selectivity achieved by **dnHEM1-SS19** compare favorably to those reported for the de novo carbene transferase C45,^24^ which contains a covalently bound heme iron cofactor, and are only marginally lower than the values reported for the engineered myoglobin H64V V68A variant.^8^

## Conclusions

The crystal structure of **dnHEM1** demonstrates the attainment of our design goal: the creation of high-affinity heme-binding proteins with an open coordination site adjacent to a large reconfigurable substrate binding cavity. The ability to alter the surface charge, embed polar residues in the distal cavity to facilitate peroxidase catalysis, and computationally design substrate binding pockets for enantiocomplementary olefin cyclopropanations all testify to the engineerability of our designed systems. There are exciting routes moving forward from this initial study. First, we have not yet taken advantage of the reconfigurability of the individual repeat units in the designed solenoid - this should enable the more complete customization of the binding pocket to accommodate new substrates of interest. Second, using cysteine rather than histidine as an axial ligand to the heme iron will open up new catalytic possibilities by allowing the generation of more potent ferryl intermediates, as illustrated by the catalytic prowess of P450s and peroxygenases. Finally, our methods can be readily extended to design customized proteins that bind a range of biological or non-natural catalytic cofactors to extend the range of accessible chemistries;^55−57^ the pore size and internal geometry of α helical solenoids can readily be varied.^40^ We anticipate the enzymes and design principles presented here will serve as a platform for the creation of hyperstable biocatalysts for diverse applications.

## Supporting Information Available

Data supporting the findings of this work, including methods and protocols, supplementary figures and tables, sequence, and crystallographic data are available within the Supplementary Information files (PDF)
Energies and molecular coordinates of calculated structures are provided in the Supplementary Information files (XYZ)
Computational models of designed proteins (ZIP)
Scripts and files associated with computational design are available for download on GitHub^58^

## Supplementary Material

ja3c02742_si_001.pdf

ja3c02742_si_002.xyz

## Accession Codes

PDB id 8C3W contains the coordinates and associated structure factors of dnHEM1 that have been deposited in the Protein Data Bank (PDB) database.
