# Supplementary material for: Design of Heme Enzymes with a Tunable Substrate Binding Pocket Adjacent to an Open Metal Coordination Site
Source: J Am Chem Soc. 2023 Jun 21;145(26):14307–15. doi: 10.1021/jacs.3c02742 (PMC10326885; doi:10.1021/jacs.3c02742)
Supplement: Supplementary file 1 — ja3c02742_si_001.pdf [file ja3c02742_si_001.pdf]

Supporting Information

Design of Heme Enzymes with a Tunable Substrate Binding Pocket Adjacent to an Open Metal Coordination Site

Indrek Kalvet[‡1,2,4], Mary Ortmayer[‡3], Jingming Zhao[‡3], Rebecca Crawshaw[3], Nathan M. Ennist[1,2], Colin Levy[3], Anindya Roy*[1,2], Anthony P. Green*[3], David Baker*[1,2,4]

[‡]Equal contribution
*To whom correspondence should be addressed
[1]Institute for Protein Design, University of Washington, 3946 W Stevens Way NE, Seattle, WA 98195.
[2]Department of Biochemistry, University of Washington, Seattle, WA 98195. [3]Manchester Institute of Biotechnology, School of Chemistry, 131 Princess Street, University of Manchester, Manchester M1 7DN, UK. [4]Howard Hughes Medical Institute, University of Washington, Seattle, WA 98195.

Contents

1. Experimental procedures 2
1.1. Characterization of de novo designed heme binding proteins 8
1.2. Analysis of dnHEM1 mutants 11
1.3. Peroxidase evolution 18
1.4. Olefin cyclopropanation studies 26
1.5. Amino acid and DNA sequences of de novo designed heme binding proteins 33
2. Crystallographic data 44
3. Computational details 46
3.1. Heme binding site design 46
3.2. DFT optimization of transition states 53
3.3. Carbene transferase active site design 55
3.4. Energetic and thermal data for computed structures 58
4. References 63

1. Experimental procedures

Materials

All materials were obtained from commercial suppliers and used as received, unless stated otherwise. Chemicals were sourced from Sigma-Aldrich unless otherwise stated. Flash column chromatography was performed with Merck silica gel 60 (35–70 mesh). Polymyxin B sulfate was purchased from Alfa Aesar; LB-agar Miller, LB Miller media and 2×YT media from Formedium; Terrific Broth II (TB-II) from MP Biomedical; *Escherichia coli* (*E. coli*) BL21 DE3 (#C2527I; NEB), *E. coli* 5 alpha (DH5α), Q5 DNA polymerase, Phusion polymerase, Gibson Master Mix, T4 DNA ligase and restriction enzymes from New England BioLabs (NEB). Oligonucleotides and genes were synthesized by Integrated DNA Technologies (IDT).

^{1}H and ^{13}C NMR spectra were recorded on a Bruker Advance (^{1}H at 400 MHz, ^{13}C at 100 MHz) spectrometer. ^{1}H and ^{13}C spectra are referenced to residual solvent signals; $CDCl_3$ 7.26 ppm for ^{1}H and 77.0 ppm for ^{13}C. Coupling constants (J) are reported in Hz and coupling patterns are described as d = doublet, q = quartet, m = multiplet.

Construction of pET29b(+) dnHEM1 and variants

Genes encoding the 22 designs (denoted with the prefix 'IK_HC015_') were purchased as subcloned genes in pET29b(+) vector with an additional 19-residue C-terminal sequence containing a His-tag and SNAC cleavage site[1] between the *Nde*I and *Xho*I restriction sites (full sequence: M<design>GGSGGSHHWGSGSHHHHHH). The genes were codon optimized for *E. coli* expression. Plasmids were purchased from Integrated DNA Technologies (IDT).

The dnHEM1 H148A, dnHEM1 H148F, dnHEM1.2 H148A and dnHEM1.2B H148A mutants were generated using overlap extension PCR followed by *Nde*I and *Xho*I digestion and ligated into pET29b(+) using T4 DNA ligase. All constructs were verified using Sanger sequencing (Eurofins or GeneWiz/Azenta).

Cloning DNA sequences with Gibson assembly

Plasmids encoding the carbene transferase designs and dnHEM1 (pI=6) were generated by Gibson assembly. Double-stranded DNA fragments encoding the designs were purchased from Integrated DNA Technologies (IDT) as eBlocks™ Gene Fragments. For carbene transferase designs, the DNA sequence was assembled from two fragments through an overlapping region encoding residues A111-L116, with the 5' (N-terminal) fragment containing mutations introduced through Rosetta redesign, and the second fragment encoding the second half of the protein sequence. Overhang sequences complementary to those found in the 3' and 5' ends of the linear vector were added to the 5' end of the first and 3' end of the second fragment. An overlapping sequence of 18 nucleotides (GCGGCTTTAGCCCTGCTG) was used to assemble the two fragments. Full sequence: M<design>GGSGGSHHWGSGSLEHHHHHH)

The following DNA sequence was used as the second fragment (two-fragment assembly overhang highlighted in **bold**; vector assembly overhang highlighted in *italics*):

GCGGCTTTAGCCCTGCTGACCGCAGCTAAACTAGGCACTACAGTCGAGGAAGCTGTACAGGAGGCTTTGCAGCTTAAAACTAAACT
AGGCGTTTCACTGATAGAAGCATTGCATATCCTGTTGACGGCCGCAGTACTGGGGACAACTGTAGAAGAGGCTGTATATCGAGCGC
TTAAACTCAAGACAAAGCTAGGGGTTAGTTTGCTCCAGGCAGCGGCCATATTGATTTTAGCAGCGCGCCTGGGGACTACAGTAGAG
CAGGCTGTAGAACGCGCATTGCAACTAAAGACTAAGCTT*GGTGGCGGGAGCGGTGGCTC*

The DNA sequence of the first fragment was optimized for bacterial expression while avoiding overlapping regions of 8 nt or longer with the second DNA fragment.

Gibson assembly was performed using pET29b(+) linear DNA vector and Gibson Master Mix (#2611; NEB) according to the manual.[1,2] The assembled DNA was transformed into DH5α competent *E. coli.* The isolated plasmids were sequence-verified by Sanger sequencing using T7 and T7-Term primers.

Preparation of linear vector for Gibson assembly

A linear DNA vector encoding the SNAC cleavage site sequence and hexahistidine tag, for use in Gibson assembly reactions, was prepared by amplifying an empty pET29b(+) vector together with primers (Supplementary Table 3, GibsonV_F, GibsonV_R) using Phusion polymerase (#M0531; NEB, denaturation for 30s at 98 °C; 32 cycles of 10s at 98 °C, 30s at 64 °C, 150s at 72 °C; final extension of 10 minutes at 72 °C). PCR product was purified by agarose gel electrophoresis followed by isolation with Qiagen QIAquick Gel Extraction Kit.

Cloning DNA sequences via Golgen Gate cloning

A subset of designs were cloned using *Bsa*I restriction enzyme and T4 DNA ligase following the Golden Gate cloning protocol using Golden Gate Master Mix (#E1601, NEB).[3] Double-stranded DNA fragments encoding the designs were purchased from Integrated DNA Technologies (IDT) as eBlocks™ Gene Fragments. The DNA fragments encoding design sequences and including overhangs suitable for a *Bsa*I restriction digest were cloned into a custom pET29b(+) target vector containing lethal ccdb gene, and C-terminal SNAC and hexahistidine tags (#191551, Addgene). Full sequence: `MSG<design>GSGSHHWGSTHHHHHH`)

The assembled DNA was transformed into *E. coli* BL21(DE3) competent *E. coli.* The isolated plasmids were sequence-verified by Sanger sequencing using T7 and T7-Term primers.

Protein expression and purification

Chemically competent *E. coli* BL21(DE3) cells were transformed with the appropriate plasmid, encoding a designed protein. A single colony of freshly transformed cells was cultured for 6 h at 37 °C with shaking at 225 r.p.m. in 5 mL of TB-II medium containing 50 µg/mL kanamycin and 0.8% dextrose. These starter cultures were used to inoculate 50 mL TB-II autoinduction media containing 2 mM $MgSO_4$ and 50X diluted 5052 solution (50X 5052 = 25% glycerol, 2.5% glucose, 10% α-lactose), supplemented with 50 µg mL^{-1} kanamycin antibiotic, and grown overnight at 37 °C. Cells were harvested by centrifugation at 4100 *g* for 7 min, and resuspended in 30 mL lysis buffer (25 mM Tris-HCl, 300 mM NaCl, 25 mM imidazole, pH 8.2, and containing Pierce protease inhibitor tablet, 100 µg mL^{-1} lysozyme and 10 µg mL^{-1} deoxyribonuclease), and lysed by ultrasonication (13 mm probe, 2.5 mins, 10s on, 10s off, 65% amplitude). Cell lysates were cleared by centrifugation (15,000 g, 20 min). The cleared lysates were loaded onto nickel affinity gravity columns (Ni-NTA) to purify the designed proteins by immobilized metal-affinity chromatography (IMAC). Proteins were either eluted off the column with a buffer containing 25 mM Tris-HCl, 300 mM NaCl, 300 mM imidazole pH 8 to obtain uncleaved protein or subjected to SNAC-tag cleavage conditions (see below) to cleave off the hexahistidine tag.

SNAC-tag cleavage

Adapted from a published protocol.[1] The following protocol was applied to proteins obtained from 50-100 mL expressions, loaded onto 1-1.5 mL Ni-NTA resin. The resin is loaded with protein by incubating the lysis supernatant and the resin for 30 minutes on a nutating platform. Protein-loaded resin is subjected to 5 washing steps: 1) 20 mL lysis buffer (25 mM Tris-HCl, 300 mM NaCl, 25 mM imidazole); 2) 20 mL lysis buffer with 1M NaCl; 3) 20 mL lysis buffer; 4) 20 mL TBS (25 mM Tris-HCl, 300 mM NaCl); 5) 20 mL SNAC buffer (100 mM CHES-NaOH, 100 mM Acetone oxime, 100 mM NaCl, pH 8.6; without $NiCl_2$). Thereafter, the column is capped and 15 mL of SNAC buffer containing 2 mM $NiCl_2$ is added, and the column incubated on a nutating platform for 18 hours. SDS-PAGE was used to monitor the completion of the cleavage reaction. The flow-through fraction (containing the cleaved protein) was collected, and the resin washed with 10 mL of lysis buffer. These fractions were combined and concentrated down to 1 mL using Amicon™ Ultra-15 10K centrifugal filters.

Size-exclusion chromatography

Following IMAC purification, designs were further purified by SEC on ÄKTAxpress (GE Healthcare) using a Superdex Increase 75 10/300 GL column (GE Healthcare) in TBS buffer at 0.8 mL/min flowrate. The monomeric or smallest oligomeric fractions of each run (eluting at approximately 13.5 ml) were collected. The resulting samples were generally > 95% homogeneous on SDS–PAGE gels. SEC retention volume to molecular weight equivalencies were calibrated with protein standards (Cytiva LMW (#28403841). Further comparisons were made with selected proteins between using running buffers consisting of either 25 mM Tris, 300 mM NaCl, pH 8.2 or 50 mM KPi, 200 mM NaCl, pH 7.2.

Protein production and purification of in vitro loaded dnHEM1

Chemically competent *E. coli* BL21(DE3) cells were transformed with the appropriate pET29b(+)_dnHEM1 construct. A single colony of freshly transformed cells was cultured for 18 h in 5 mL of LB Miller medium containing 50 μg/mL kanamycin. 500 μL of the culture was used to inoculate 50 mL of 2xYT medium supplemented with 50 μg/mL kanamycin. The culture was incubated for ~2 h at 37 °C with shaking at 180 r.p.m to an optical density at 600 nm (OD_{600}) of ~0.5 A.U.. Protein expression was induced with the addition of IPTG to a final concentration of 0.1 mM. The induced cultures were incubated for ~20 h at 25 °C, and the cells were subsequently harvested by centrifugation at 3220 *g* for 10 min. The pelleted bacterial cells were suspended in lysis buffer (50 mM KPi, 300 mM NaCl, 20 mM imidazole, pH 7.5) supplemented with lysozyme (1 mg mL^{-1}), DNase (1 μg mL^{-1}) and a Complete EDTA free protease inhibitor cocktail tablet (Roche), and subjected to sonication (13 mm probe, 15 mins, 20 s on, 40 s off, 40 % amplitude). Cell lysates were cleared by centrifugation (10,000 g, 30 min). To maximize the heme occupancy, the clarified lysates were mixed with hemin to a final concentration of 20 μM (400 μM stock solution in assay buffer) for 30 mins at room temperature. The heme loaded clarified lysates were subjected to affinity chromatography using Ni-NTA Agarose (Qiagen). His-tagged variants were eluted using elution buffer (50 mM KPi, 300 mM NaCl, 250 mM imidazole, pH 7.5). The eluent containing purified protein was buffer exchanged into assay buffer (50 mM KPi, 200 mM NaCl, pH 7.2) using a 10DG column (Bio-Rad) and analyzed by sodium dodecyl sulfate-polyacrylamide gel electrophoresis (SDS-PAGE) and mass spectrometry. The concentration of heme loaded proteins were determined using the extinction coefficient of the Soret peak (as determined using a pyridine hemochromogen assay (Supplementary Table 1)).

Apo dnHEM1 production

For the expression of *apo* dnHEM1 protein, a modified M9 minimal medium (1x M9 salts, 0.2% glucose, 0.1 mM $CaCl_2$, 2mM $MgSO_4$, 4 mg mL^{-1} casamino acids, 10 µg mL^{-1} thiamine chloride) was used to minimize heme contamination. A single colony of freshly transformed cells was cultured for 18 h in 5 mL modified M9 medium containing 50 µg mL^{-1} kanamycin. A starter culture (500 µL) was inoculated to 50 mL modified M9 medium supplemented with 50 µg mL^{-1} kanamycin. The culture was grown for 3 h at 37 °C, 180 r.p.m. to OD_{600} of 0.6. Protein expression was induced with the addition of IPTG to a final concentration of 0.1 mM. The induced culture was incubated for 20 h at 25 °C (180 r.p.m.), and the cells were subsequently harvested by centrifugation (3,220 *g* for 10 min). Pelleted cells were resuspended in lysis buffer (see above) and subjected to sonication. Cell lysates were cleared by centrifugation (10,000 *g* for 30 min) and the supernatant were subjected to affinity chromatography using Ni-NTA Agarose (Qiagen). The His-tagged protein was eluted using the 10 mL elution buffer (see above), and buffer exchanged into assay buffer with a 10DG desalting column (Bio-Rad). The purified apo protein was aliquoted, flash-frozen in liquid nitrogen and stored at -80 °C immediately. The protein concentration was determined spectrophotometrically on a NanoDrop (Thermo Fisher) with an extinction coefficient of 12,490 M^{-1} cm^{-1} (calculated using ProtParam Expasy) at 280 nm.

Hemoprotein extinction coefficient calculations

The pyridine hemochromagen assay was used to determine the extinction coefficient of the Soret maximum, according to the method of Berry and Trumpower (Supplementary Table 1).[4]

Supplementary Table 1. The extinction coefficients of Soret band and R_z values (A_{Soret} / A_{280}) of *holo* dnHEM1 and variants.

	**Extinction coefficient ($mM^{-1} \cdot cm^{-1}$)**	**R_z**
dnHEM1	116 ± 8	5.62
dnHEM1.2	128 ± 1	3.92
dnHEM1.2B	111 ± 2	3.83
dnHEM1-SS19	95 ± 1	3.51
dnHEM1-RR2	104 ± 1	4.14

MS analysis

MS data for dnHEM1 variants were acquired on a 1200 series LC QTOF 6510 MS (Agilent). The final protein concentrations were adjusted to 10 µM in 50 mM KPi 200 mM NaCl pH 7.2. A sample volume of 5 µL of sample was injected onto the LC-MS system and desalted inline with 1 mL min^{-1} 5% acetonitrile (0.1% formic acid). Protein was eluted over 1 minute by 95% acetonitrile. The resulting multiply charged spectrum was analyzed by an QTOF 6510 (Agilent) in ESI positive ion mode, and deconvoluted using Masshunter Software (Agilent). The instrument was tuned and calibrated with reference solution.

Alternatively, an Agilent 1200series LC G6230B TOF LC-MS with an AdvanceBio RP-Desalting column was used (A: H_2O with 0.1% Formic Acid, B: Acetonitrile with 0.1% Formic Acid). Final protein concentrations were adjusted to 1-2 mg mL^{-1} in 30 mM Tris-HCl 300 mM NaCl pH 8.2. Subsequent data deconvolution was performed in Bioconfirm using a total entropy algorithm. All data are presented in Supplementary Table 2.

Supplementary Table 2. Mass spectrometry data for dnHEM1 variants. 'Cleaved' refers to proteins obtained upon cleavage of the hexahistidine tag via the SNAC-cleavage site.

Variant	Expected Mass	Observed Mass
dnHEM1	24029	24029
dnHEM1 (cleaved)	22371	22375
dnHEM1_H148A (cleaved)	22304	22305
dnHEM1_H148F (cleaved)	22381	22381
dnHEM1_pI6 (cleaved)	22261	22261
dnHEM1.2	24075	24075
dnHEM1.2B	24194	24193
dnHEM1-SS19 (pI=10)	24102	24100
dnHEM1-SS19 (pI=6)	24190	24191
dnHEM1-RR2 (pI=10) (Met missing)	23915	23915
dnHEM1-RR2 (pI=6)	24136	24135

Spectrophotometric heme binding assay

To qualitatively determine the heme binding ability of the de novo designed proteins, UV−Vis spectra were measured of the protein and hemin mixture using an Agilent Cary 8454 or Jasco Spec V750 spectrophotometer with a 10 mm pathlength cuvette. Spectra in the 230-700 nm range were collected of solutions containing 30 µM of purified protein and 10 µM of hemin (unless stated otherwise). Samples were prepared by mixing 5 µL of hemin solution (200 µM stock solution in DMF) into a protein solution in TBS buffer, adding up to a total volume of 100 µL. Data are presented in Supplementary Fig. 3.

Spectrophotometric heme titration and determination of dissociation constant

To determine the affinity of dnHEM1 for ferric heme B, we performed a binding titration following methods reported previously.[5] Briefly, a heme stock solution was prepared in DMSO with a concentration of 150 to 400 µM heme as determined by pyridine hemochromagen assay.[4] We prepared 2.5 mL of 0.4 to 1.5 µM *apo*-dnHEM1 in aqueous buffer with 200 mM NaCl, 50 mM potassium phosphates, pH 7.3, and 0.5% w/v octyl-β-glucoside to minimize aggregation. The protein solution was added to a 1-cm pathlength quartz cuvette with a stir bar, and an absorbance spectrum was recorded with a Jasco V-750 UV−vis spectrophotometer. Aliquots of heme stock solution were added to the protein sample with stirring at 25°C. After each aliquot was added, the protein-heme mixture was allowed to equilibrate for at least 10 minutes, at which point another absorbance spectrum was recorded. Heme aliquots were added and spectra recorded until a 2.5-fold excess of heme had been added in total. Absorbance values at 402 nm (the Soret maximum in heme-bound state) were plotted against heme concentration (Figure 2B), and the data were fitted using Origin 8.1 to a one-site binding equation:

$$A = H_{tot}\varepsilon_{free} + \left(\varepsilon_{bound} - \varepsilon_{free}\right)\frac{K_d + P_{tot} + H_{tot} - \sqrt{(K_D + P_{tot} + H_{tot})^2 - 4P_{tot}H_{tot}}}{2}$$

In this equation, the total absorbance at 402 nm is given as A. The total heme concentration is $H_{tot,}$, P_{tot} represents the total protein concentration, K_d is the dissociation constant, and ε is the extinction coefficient of heme when it is free in solution or bound to protein. The collected spectra and absorbance-[heme] curves are depicted in Supplementary Fig. 10.

Variable temperature spectrophotometric measurements

To observe changes in the spectral properties of bound heme at increasing temperatures, UV−Vis spectra were measured of in vitro loaded holo-proteins using the Jasco Spec V750 spectrophotometer and a 10 mm pathlength cuvette. Spectra in the 230-800 nm range were collected at every 10 °C intervals between 25 °C and 95 °C. Temperature was increased at the rate of 5 °C min^{-1}, and spectra were acquired after the temperature had stabilized to within 0.5 °C of target temperature for 5 seconds. Measurements were performed with 20 µM solutions of purified holoprotein in TBS buffer (25 mM Tris-HCl, 300 mM NaCl, pH 8). Data are presented in Supplementary Fig. 16 and Fig. 26.

pH-dependent spectrophotometric measurements

To observe changes in the spectral properties of bound heme at various pH levels, UV−Vis spectra were measured of the mixture of 10 µM *apo*-dnHEM1 and 2 µM hemin, the in vitro loaded holo-proteins (7.5 µM dnHEM1, dnHEM1-RR2, dnHEM1-SS19), and 2 µM free hemin using the Jasco Spec V750 spectrophotometer and a 10 mm pathlength cuvette. Spectra in the 230-800 nm range were collected at at ph levels 3, 4, 5, 6, 7, 8, 9 and 10. The universal Britton-Robinson buffer system was used across the entire pH range to ensure comparable buffer conditions. The buffer consists of 150 mM NaCl and equimolar quantitites (40 mM) of H_3PO_4, $B(OH)_3$ and acetic acid, with the pH adjusted using NaOH. Any particulate was removed by filtration through a 0.22 µm filter before use.

The samples were prepared by mixing 151 µL of the pH-buffer with 9 uL of the protein solution (137 µM, in 50 mM KPi buffer containing 200 mM NaCl). Hemin samples were prepared by mixing 158 uL of the pH-buffer with 2 µL of 150 µM solution of hemin in DMSO. Data are presented in Supplementary Fig. 13.

Circular dichroism

To determine secondary structure and thermostability of the designs, far-ultraviolet circular dichroism (CD) measurements were carried out on a JASCO J-1500 instrument. The 200 to 260 nm wavelength scans were measured at every 10 °C intervals from 25 °C to 95 °C. Temperature was increased at the rate of 2 °C min^{-1}, and spectra were acquired after the temperature had stabilized to within 0.1 °C of target temperature for 5 seconds. Wavelength scans and temperature melts were performed using 0.40 mg mL^{-1} protein in 25 mM Tris-HCl, 30 mM NaCl buffer at pH 8.2 with a 1 mm path length cuvette. Protein concentrations were determined by absorbance at 280 nm, measured using a NanoDrop spectrophotometer (Thermo Scientific) using predicted extinction coefficients.[6] Data are presented in Supplementary Fig. 7, Fig. 15 and Fig. 25.

1.1. Characterization of de novo designed heme binding proteins

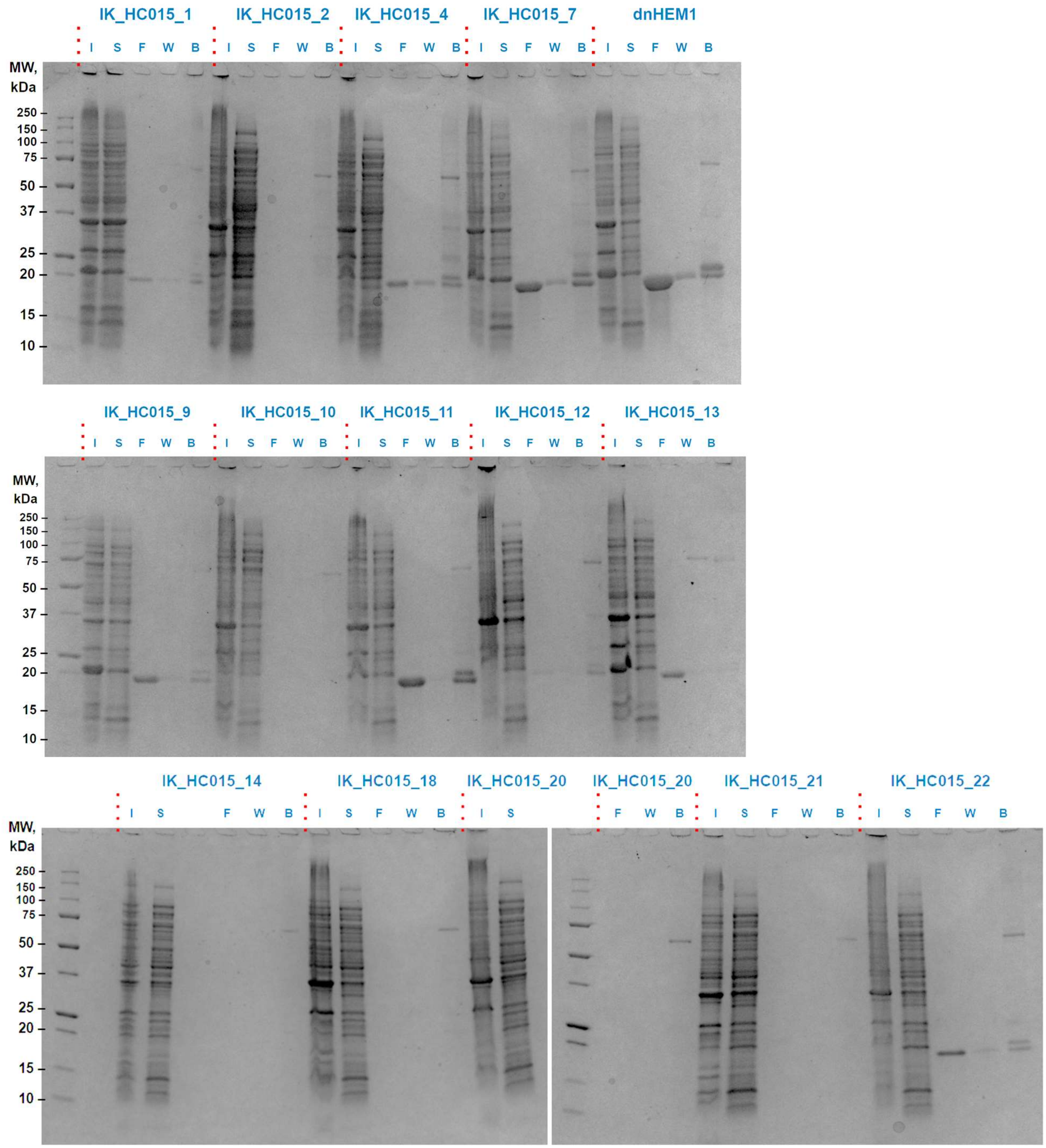

Supplementary Fig. 1. SDS-PAGE analysis of selected designed heme binding proteins before and after SNAC cleavage reaction. I = insoluble pellet; S = soluble fraction; F = flow-through after SNAC cleavage; W = wash fraction after SNAC cleavage; B = sample from Ni-NTA beads after SNAC cleavage reaction.

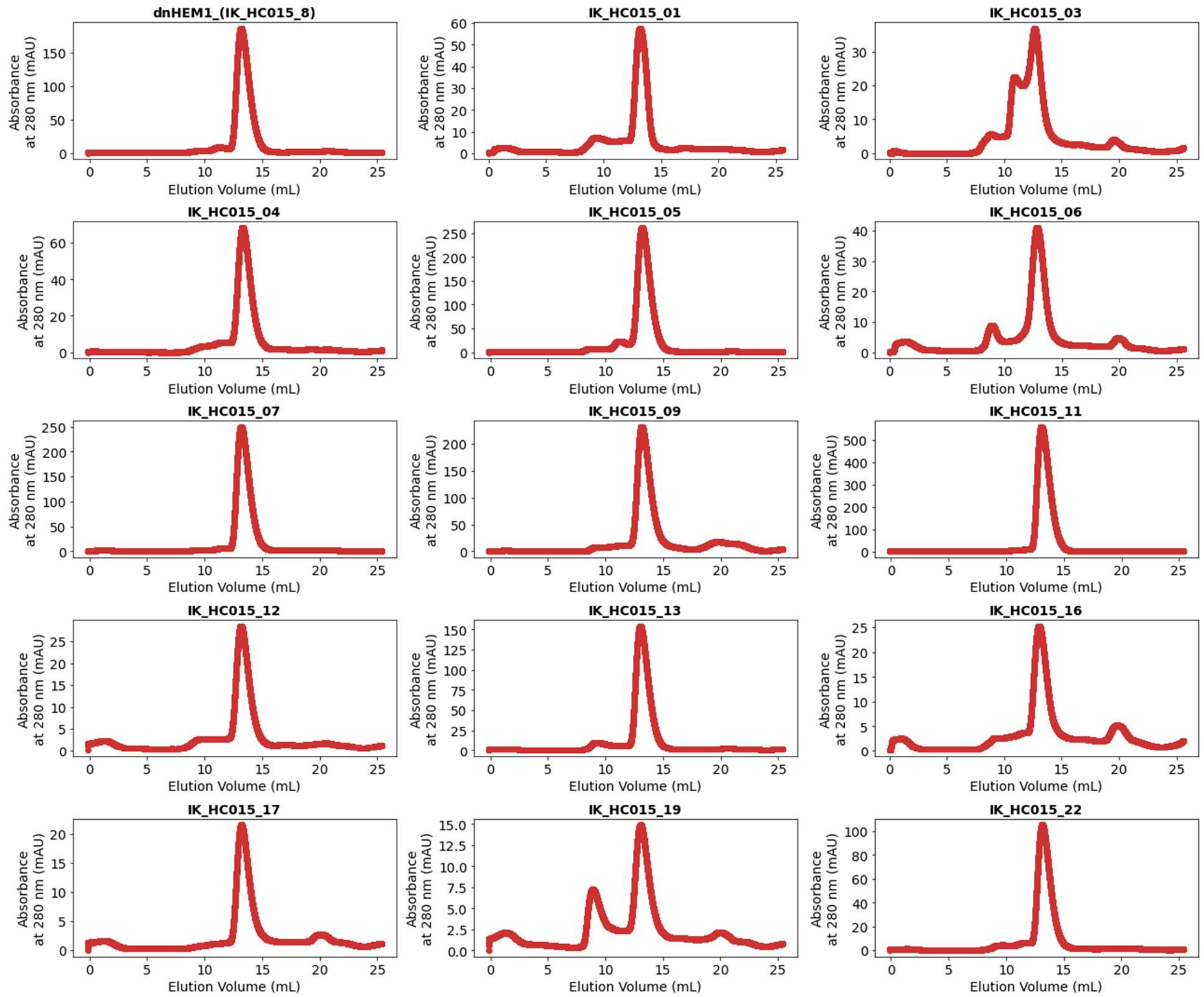

Supplementary Fig. 2. Size-exclusion chromatograms of designed heme binding proteins after SNAC cleavage reaction. Data were collected using a Superdex Increase 75 10/300 GL column (GE Healthcare) in a buffer containing 25 mM Tris-HCl and 300 mM NaCl at pH 8.2. Void volume of the column is 8.5 mL.

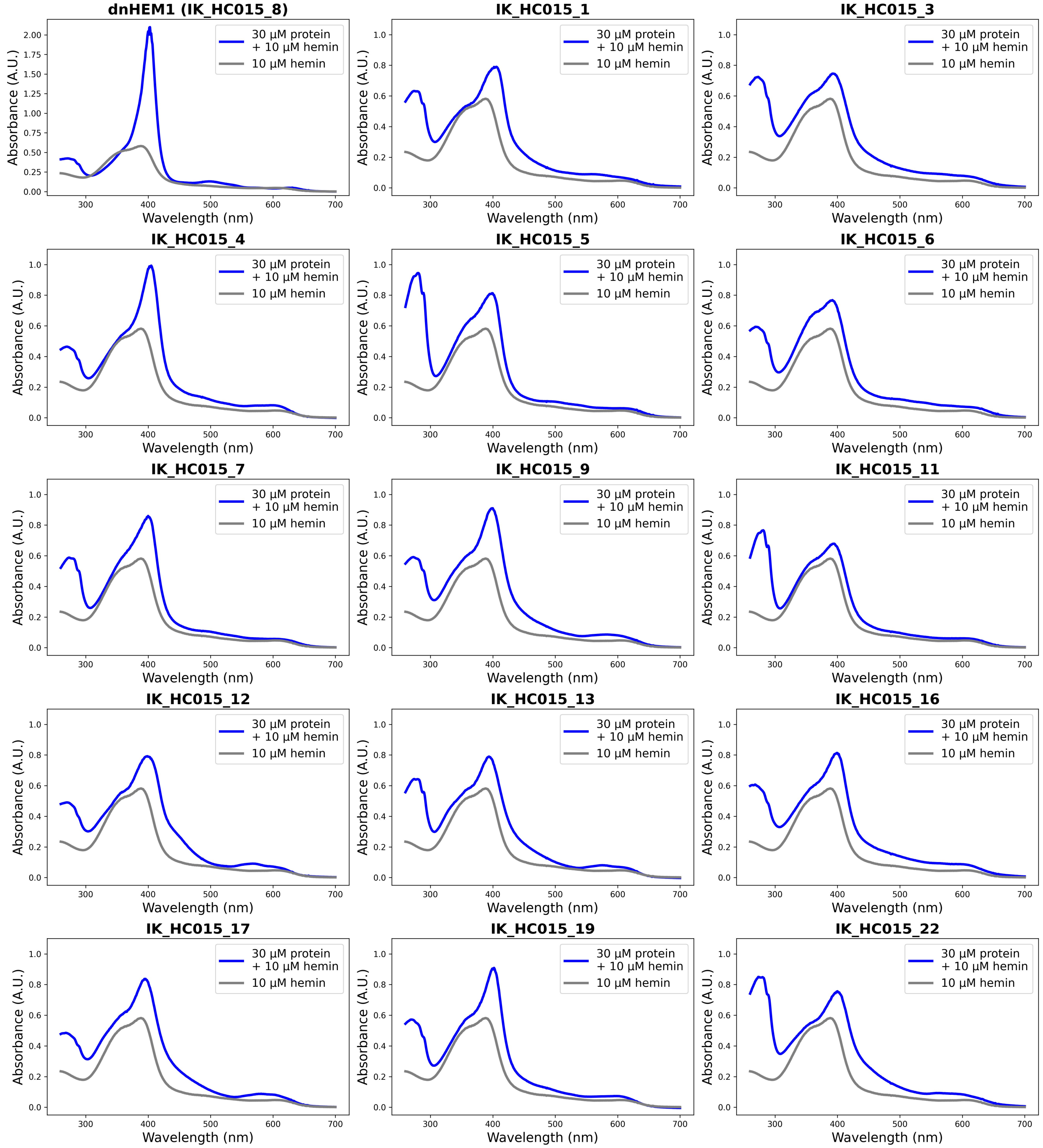

Supplementary Fig. 3. UV−Vis spectra recorded for 30 μM de novo designed proteins mixed with 10 μM hemin (blue trace), and 10 μM free hemin (gray trace).

1.2. Analysis of dnHEM1 mutants

The pI of dnHEM1 was lowered from 10.0 to 6.0 in order to bring it closer to most naturally occurring proteins, and to determine how it affects its ability to bind heme. This was achieved by mutating 12 arginine and lysine residues on the surface of the protein to GLU, ASN or GLN: K25Q, K60Q, R61E, K64Q, K95N, K99N, K130Q, R131E, K134Q, K200Q, R201E, K204Q.

The H148A and H148F mutants of dnHEM1, as well as the low pI variant, were expressed following a standard protocol as described above (including the SNAC-tag cleavage). Size-exclusion chromatography indicated that mutating the His148 or the surface Arg and Lys residues had no effect on the oligomerization state (Supplementary Fig. 6 and 8A).

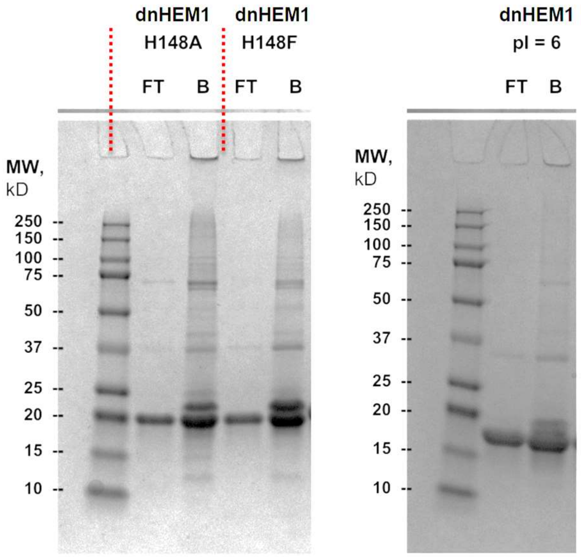

Supplementary Fig. 4. SDS-PAGE analysis of dnHEM1 mutants after SNAC cleavage reaction. FT = flow-through fraction containing cleaved protein; B = cleaved and uncleaved protein remaining bound to the Ni-NTA resin.

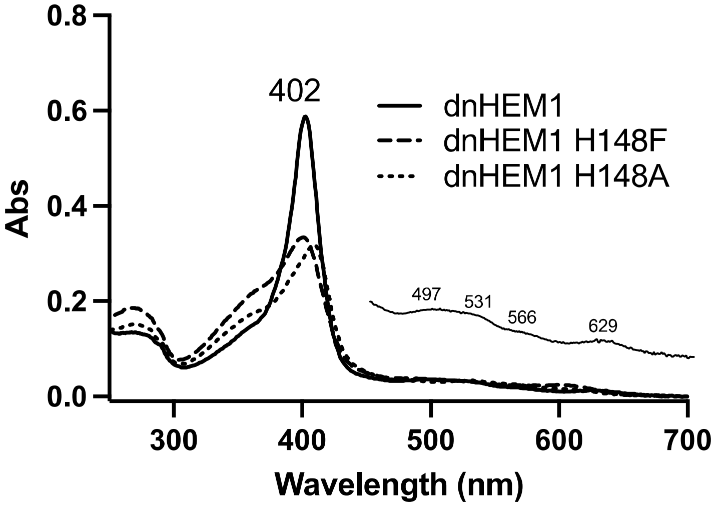

Supplementary Fig. 5. UV−Vis spectra of purified *holo* dnHEM1 (black line), dnHEM1 H148A (black dotted line) and dnHEM1 H148F (black dashed line). dnHEM1 is characterized by a Soret maximum at 402 nm with associated Q bands at 497/531/566/629 nm.

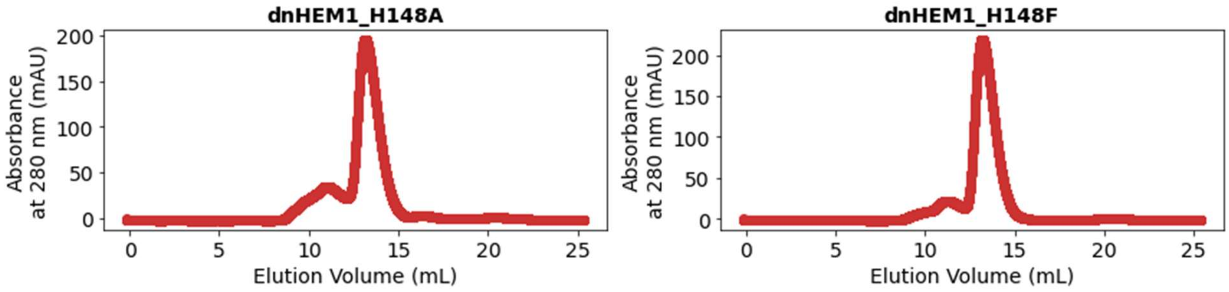

Supplementary Fig. 6. Size-exclusion chromatograms of dnHEM1 H148 mutants after SNAC cleavage reaction. Data were collected using a Superdex Increase 75 10/300 GL column (GE Healthcare) in a buffer containing 25 mM Tris-HCl and 300 mM NaCl at pH = 8.2. Void volume of the column is 8.5 mL.

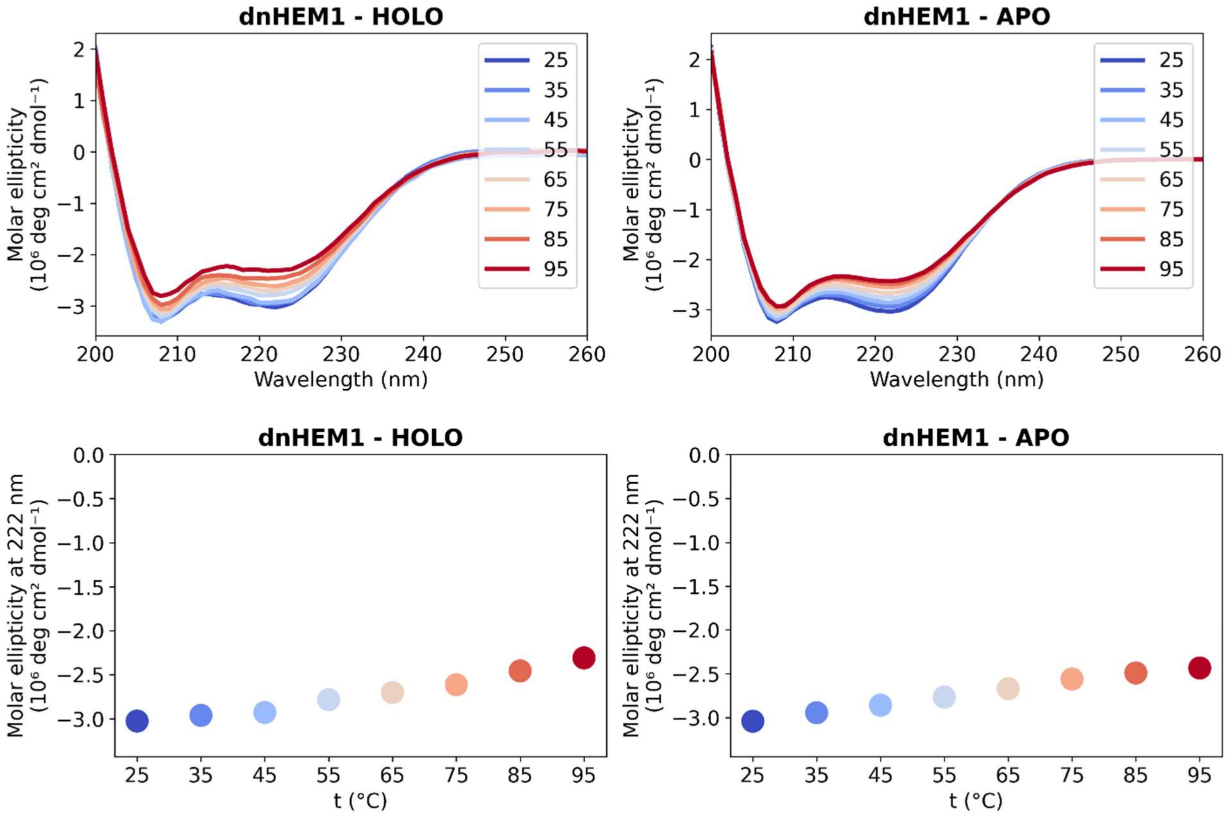

Supplementary Fig. 7. Top: circular dichroism (CD) spectra of *holo*- and *apo*-dnHEM1, measured at 15 μM protein concentration by increasing the temperature from 25 to 95 °C at 0.4 mg mL^{-1} protein concentration in 25 mM Tris-HCl, 30 mM NaCl buffer at pH = 8.2. Bottom: changes in the molar ellipticity at 222 nm while increasing the temperature.

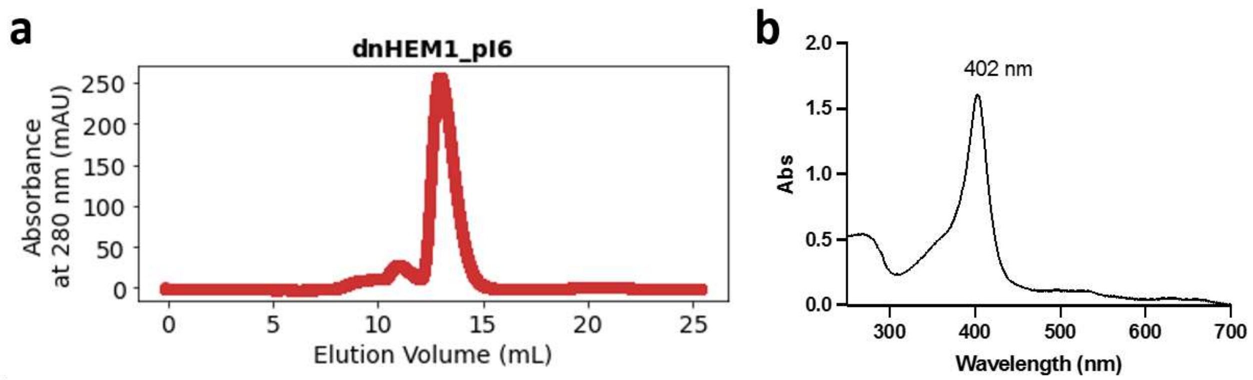

Supplementary Fig. 8. (a) Size-exclusion chromatogram of SNAC-cleaved dnHEM1 pI=6 mutant. Data were collected using a Superdex Increase 75 10/300 GL column (GE Healthcare) in a buffer containing 25 mM Tris-HCl and 300 mM NaCl at pH = 8.2. Void volume of the column is 8.5 mL. (b) UV−Vis spectra of purified *holo* dnHEM1 (pI=6) in assay buffer at room temperature.

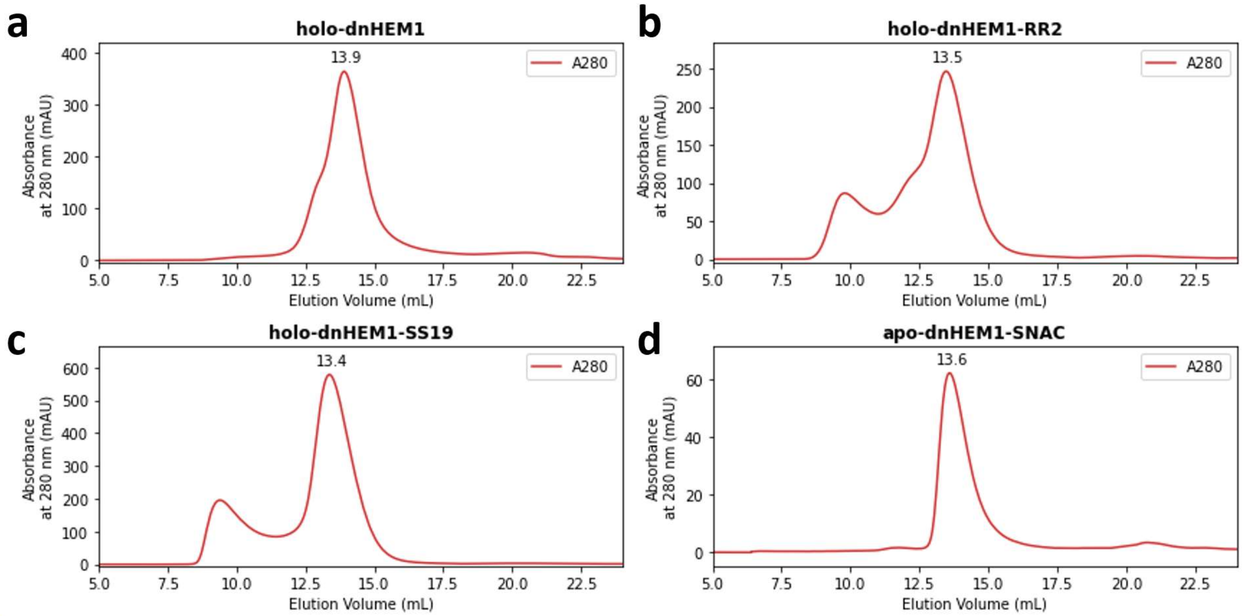

Supplementary Fig. 9. (a) Size-exclusion chromatogram of *apo*- and *holo*-dnHEM1 (a, d), *holo* dnHEM1-RR2 (b) and *holo* dnHEM1–SS19 (c), eluted in 50 mM KPi buffer containing 200 mM NaCl at pH 7.2. Data were collected using a Superdex Increase 75 10/300 GL column (GE Healthcare). Some degree of oligimerization can be observed under these buffer conditionsas indicated by the peak at 9.5 mL elution volume, and the shoulder at 12.5 mL elution volume.

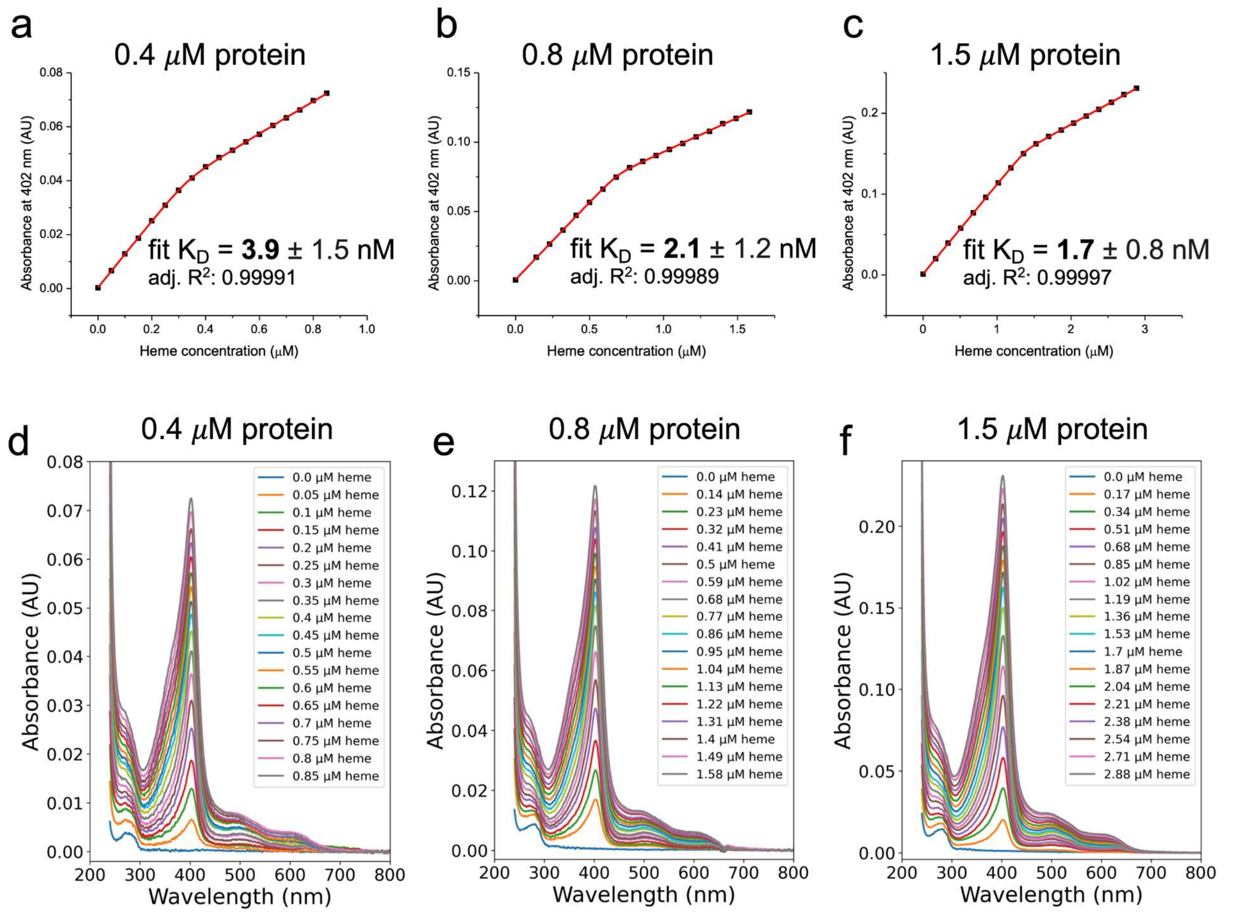

Supplementary Fig. 10. Protein-heme binding titrations using dnHEM1 protein at different concentrations. (a-c) Heme was titrated into samples with the indicated concentrations of dnHEM1 protein as described in the Supplemental Experimental Procedures. The absorbance at 402 nm was plotted against heme concentration and fitted to a one-site binding equation. The fitted dissociation constants (K_d's) are given in the plots with the standard errors of the fits, along with the adjusted R^2 values from curve fitting in Origin 8.1. For the three titrations, the mean of the fitted extinction coefficients at 402 nm was 116,000 ± 7,600 $M^{-1}cm^{-1}$ in the heme-bound state and 53,000 ± 5,500 $M^{-1}cm^{-1}$ in the unbound state. The mean K_d for the three titrations was 2.5 ± 1.2 nM. (See Supplementary Fig. 11 for spectra of bound and unbound heme). Despite the agreement in the fitted K_d's between the three titrations and the high goodness of fit values, we note that because the titrations were carried out at concentrations substantially higher than the K_d, the accuracy of the K_d measurement may be poorer than the standard errors would suggest. Significantly lower concentrations would have had low signal-to-noise ratios. Nevertheless, the sharp change in slope we observe at a stoichiometric heme:protein ratio clearly indicates high affinity binding with a K_d significantly below the protein concentration, likely <10 nM. (d-f) The full absorbance spectra that correspond to the data shown in (a-c). Buffer conditions were 200 mM NaCl, 50 mM potassium phosphate, pH 7.3, and 0.5% w/v octyl-β-glucoside.

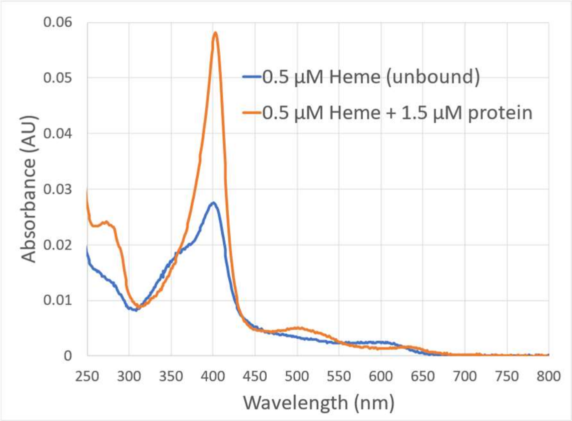

Supplementary Fig. 11. UV/vis absorbance spectra of heme with and without dnHEM1 protein present. The buffer conditions are the same as for the heme binding titration shown in Supplementary Fig. 10: 200 mM NaCl, 50 mM potassium phosphates, pH 7.3, and 0.5% w/v octyl-β-glucoside. The absorbance feature at 280 nm in the protein-containing trace (orange) is attributable to Trp and Tyr residues in the protein. The Soret band at 402 nm originates from heme. The heme extinction coefficient at 402 nm is significantly higher in the protein-bound state (orange) than in the unbound state (blue); this change in extinction coefficient allows binding to be monitored spectroscopically as in Supplementary Fig. 10.

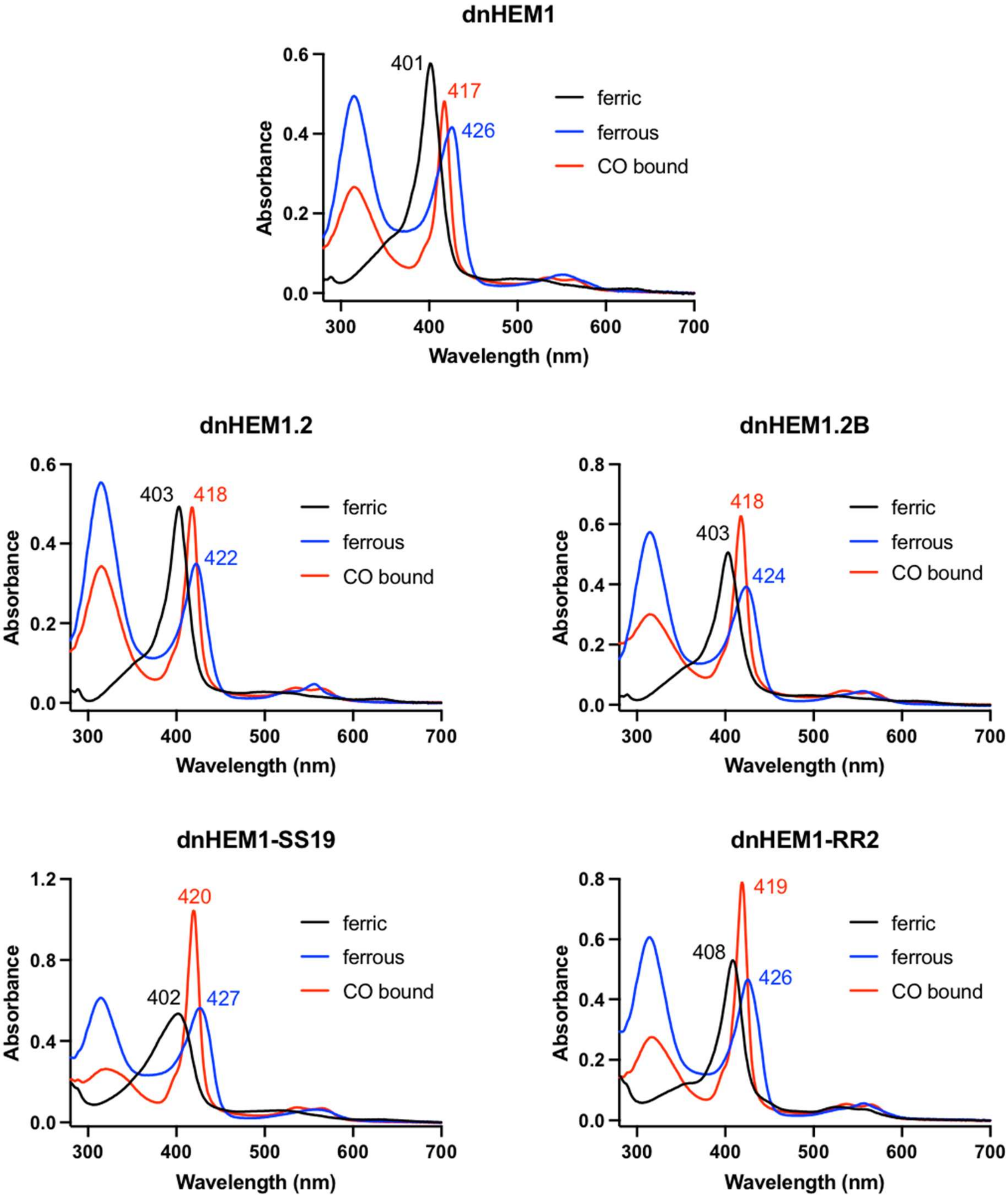

Supplementary Fig. 12. The spectra of ferric (black), ferrous (blue) and CO bound state (red) of the dnHEM1 variants. (experimental procedures: In a N_2 glovebox, 5 μM heme protein was reduced by 100 μM dithionite. The spectra of both ferric and ferrous states were recorded on a UV spectrometer inside the glovebox. The ferrous solution was transferred into an air-tight cuvette with a rubber cap. The cuvette was removed out of the glovebox and gently flushed through CO for 1 min in a fume hood. The UV spectra of the resulting solution was immediately recorded.)

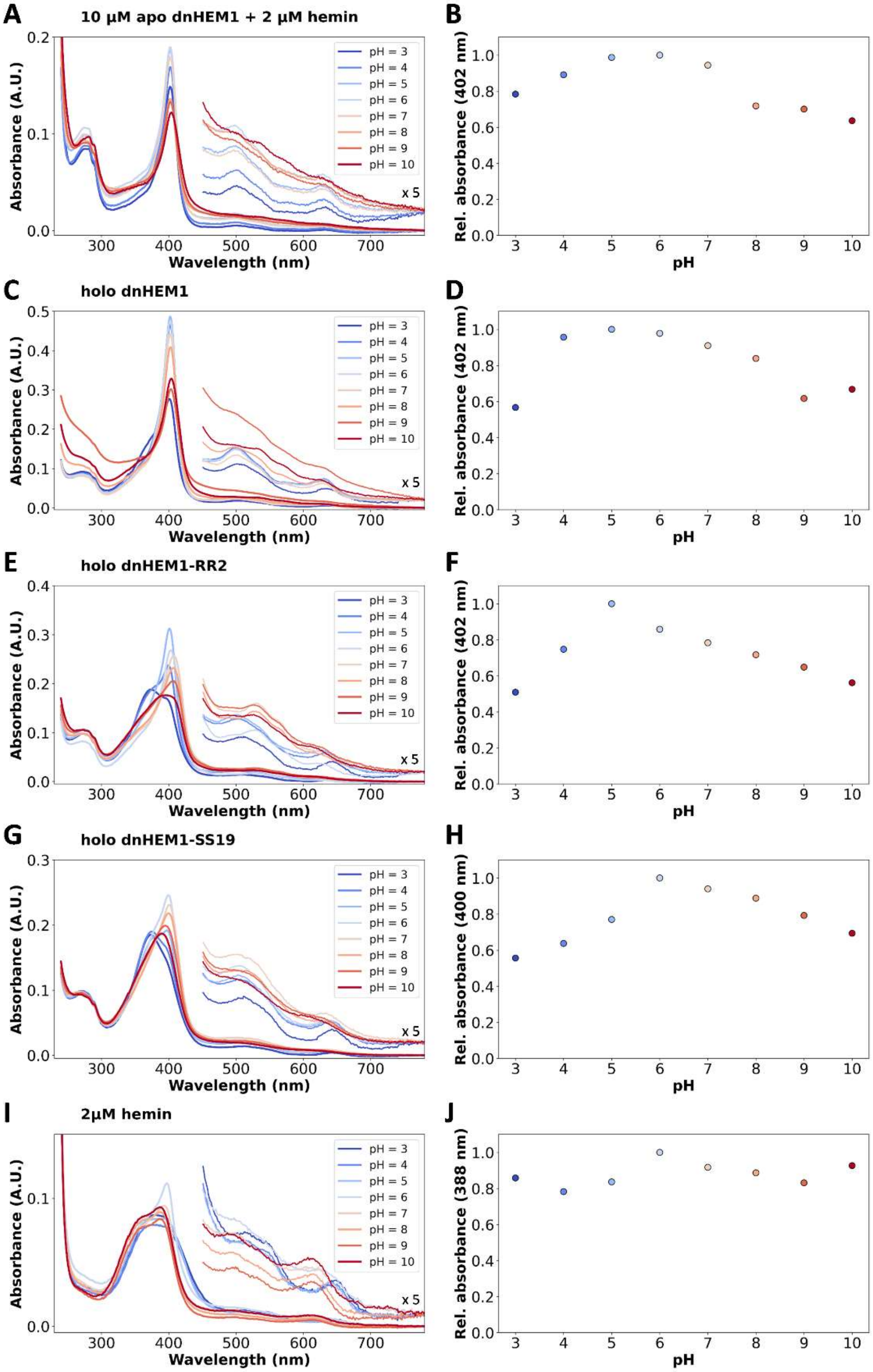

Supplementary Fig. 13. dnHEM1 retains its ability to bind heme at pH levels ranging from 3 to 10. All spectra were recorded in pH-adjusted 40 mM Britton-Robinson buffer containing 150 mM NaCl. UV/Vis spectra at different pH levels of: (a) the mixture of 10 µM dnHEM1 and 2 µM hemin; (c) 7.5 µM *holo* dnHEM1; (e) 7.5 µM holo dnhEM1-RR2; (g) 7.5 µM *holo*-dnHEM1-SS19; (i) 2 µM hemin. Insets show the changes in the Q band region at 5x magnification. Aggregation is observed at pH 9 and 10 with *holo*-dnHEM1. (b, d, f, h, j) Relative changes of the absorbance of the Soret maximum.

1.3. Peroxidase evolution

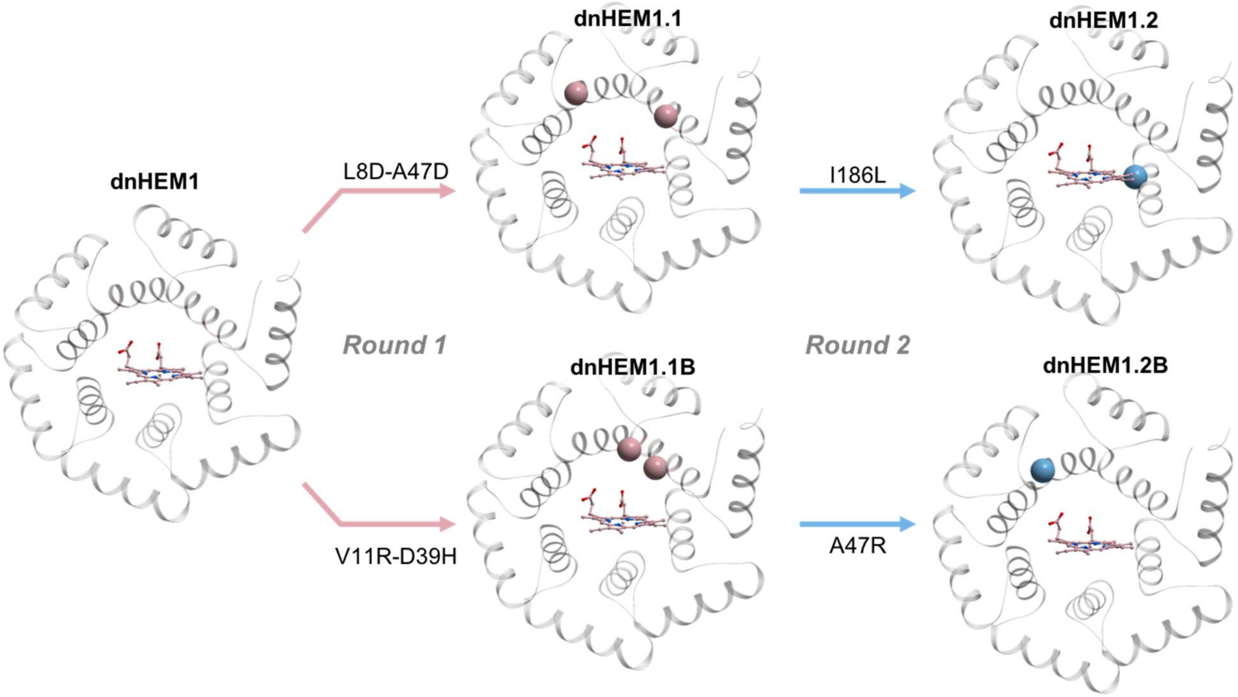

Round	Description	Clones screened	Beneficial mutations	Best Variants
1	**Saturation mutagenesis** of active site positions: D4, Q5, L8, V11, D39, L42, R43, A47, E109, L113, L116, L121, E145, L147, I149, L151, T152, I186	1584	D4F L8D V11R D39H A47D I186G	dnHEM1.1 = dnHEM1 + L8D-A47D<br>dnHEM1.1B = dnHEM1 + V11R-D39H
2	**Saturation mutagenesis** of active site positions: D4, V11, D39, L42, R43, I186	528	I186L	dnHEM1.2 = dnHEM1.1 + I186L
2	**Saturation mutagenesis** of active site positions: D4, L8, L42, R43, A47, I186	528	A47R	dnHEM1.2B = dnHEM1.1B + A47R

Supplementary Fig. 14. **Directed evolution of dnHEM1 to afford an efficient peroxidase**
Schematic showing the divergent trajectory from dnHEM1 to dnHEM1.2 and dnHEM1.2B. Mutations introduced are represented as Corey–Pauling–Koltun (CPK) spheres at the C-alpha. The first round of evolution afforded dnHEM1.1 and dnHEM1.1B, which both contain two mutations when compared to the original design dnHEM1. A second round of divergent evolution afforded dnHEM1.2 and dnHEM1.2B, which both contain three mutations compared to the original design dnHEM1. Library generation method, targeted positions, the number of clones evaluated, beneficial mutations and the most active variant for each round are given in the associated table.

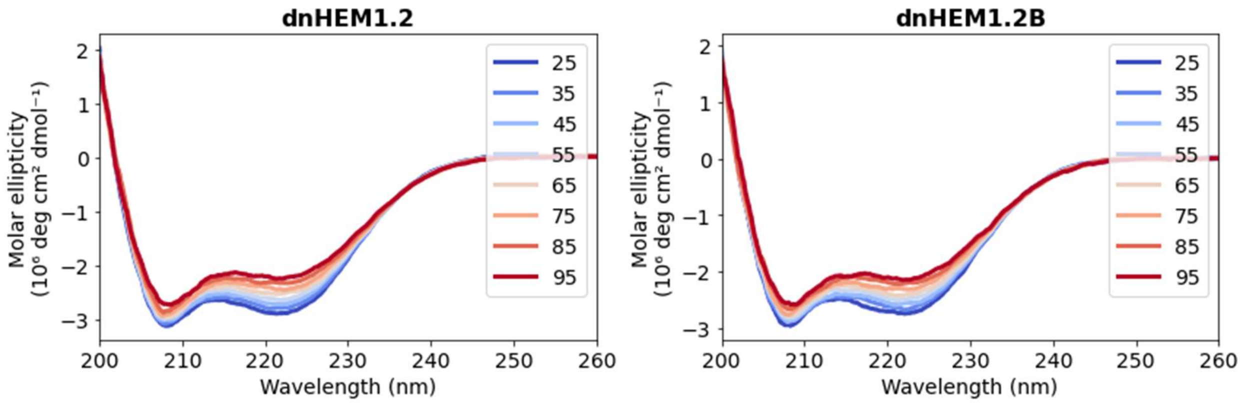

Supplementary Fig. 15. Circular dichroism (CD) spectra of *holo*-dnHEM1.2 and *holo*-dnHEM1.2B recorded at temperatures from 25 °C to 95 °C at 0.4 mg mL^{-1} protein concentration in 25 mM Tris-HCl, 30 mM NaCl buffer at pH = 8.2.

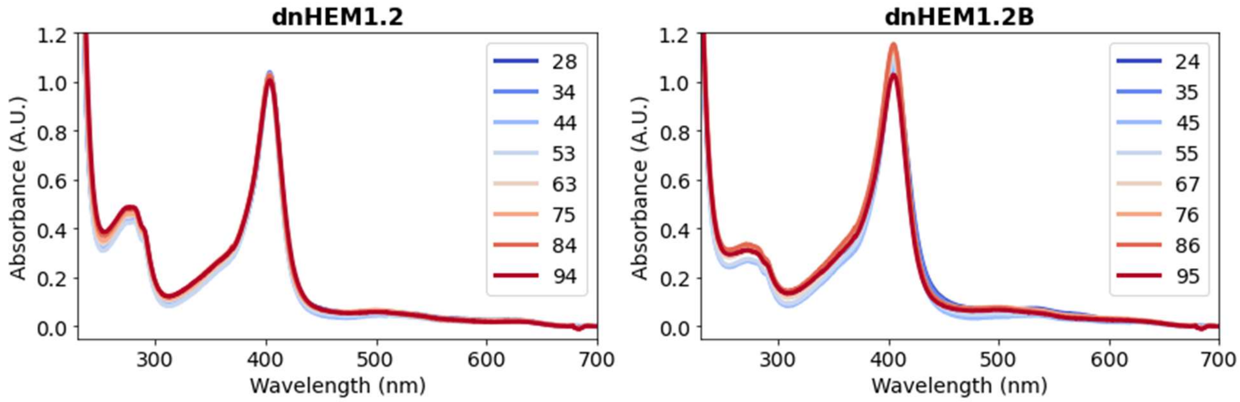

Supplementary Fig. 16. UV−Vis spectra of dnHEM1.2 and dnHEM1.2B recorded while increasing temperature from 25 °C to 95 °C at 20 µM protein concentration in 25 mM Tris-HCl, 30 mM NaCl buffer at pH = 8.2.

<u>Library construction</u>

Round 1: *saturation mutagenesis.* 18 positions were randomized independently using pET29b(+)_dnHEM1 as a template and primers with degenerate NNK codons (primer sequences shown in Supplementary Table 3). DNA libraries were constructed by overlap extension polymerase chain reaction (PCR). The linear library fragments and the pET29b(+) vector were digested using *Nde*I and *Xho*I endonucleases, gel-purified and subsequently ligated using T4 DNA ligase in a 5:1 ratio respectively.

Round 2: *divergent saturation mutagenesis.* The two most active clones from the first round of mutagenesis and screening (dnHEM1.1 and dnHEM1.1B) served as the templates for a second round of divergent evolution pathways. 6 positions were randomized independently by overlap extension PCR (primer sequences shown in Supplementary Table 3) and cloned as described above. The two most active clones of Round 2 were dnHEM1.2 and dnHEM1.2B.

Supplementary Table 3. Primer sequences

**Gibson Assembly Plasmid Amplification Primers**	
GibsonV_F	GGTGGCGGGAGCGGTGGCTCCCATCATTGGGGCAGCGGCAGCCTCGAGCACCACCACCAC
GibsonV_R	CATATGTATATCTCCTTCTTAAAGTTAAACAA
**Flanking Primers**	
*Nde*I_F	GAGATATACATATGGTGAGCCT
*Xho*I_R	AATGGTGGTGCTCGA
**Round 1**	
D4X_F	GAGATATACATATGGTGAGCCTG**NNK**CAGGCGATTCTGATTCTG
Q5X_F	GAGATATACATATGGTGAGCCTGGAT**NNK**GCGATTCTGATTCTGGTG
L8X_F	GAGATATACATATGGTGAGCCTGGATCAGGCGATT**NNK**ATTCTGGTGGTGGC
V11X_F	GAGATATACATATGGTGAGCCTGGATCAGGCGATTCTGATTCTG**NNK**GTGGCGGCGAAAC
D39X_F	TTAGGCGTGTCGTTG**NNK**CAGGCGCTGCG
D39X_R	CAACGACACGCCTAATTT
L42X_F	TCGTTGGACCAGGCG**NNK**CGTATTCTGAGCGCG
L42X_R	CGCCTGGTCCAAC
R43X_F	TTGGACCAGGCGCTG**NNK**ATTCTGAGCGCGG
R43X_R	CAGCGCCTGGTC
A47X_F	CTGCGTATTCTGAGC**NNK**GCCGCCAATACCG
A47X_R	GCTCAGAATACGCAGC
E109X_F	TTGGGCGTGGATCTG**NNK**ACCGCGGCCTTA
E109X_R	CAGATCCACGCCCA
L113X_F	CTGGAAACCGCGGCC**NNK**GCGTTGTTGACCGC
L113X_R	GGCCGCGGTTTC
L116X_F	GCGGCCTTAGCGTTG**NNK**ACCGCAGCCAAGTTA
L116X_R	CAACGCTAAGGCCG
L121X_F	TTGACCGCAGCCAAG**NNK**GGTACGACCGTTGAG
L121X_R	GGCTGCGGTCAAC
E145X_F	GGTGTGAGCTTGATT**NNK**GCACTGCATATTCTGCT
E145X_R	AATCAAGCTCACACCCA
L147X_F	AGCTTGATTGAGGCA**NNK**CATATTCTGCTGACTGCC
L147X_R	TGCCTCAATCAAGCTCA
I149X_F	ATTGAGGCACTGCAT**NNK**CTGCTGACTGCCG
I149X_R	ATGCAGTGCCTCAATC
L151X_F	GCACTGCATATTCTG**NNK**ACTGCCGCGGT
L151X_R	CAGAATATGCAGTGCCTC
T152X_F	CTGCATATTCTGCTG**NNK**GCCGCGGTGTTAG
T152X_R	CAGCAGAATATGCAGTGC
I186X_F	GCGGCTGCCATCTTG**NNK**TTAGCAGCCCGCC
I186X_R	CAAGATGGCAGCCG
**Round 2 (L8D/A47D as the template)**	
D4X_F	GAGATATACATATGGTGAGCCTG**NNK**CAGGCGATTGATATTCTGG
V11X_F	GAGATATACATATGGTGAGCCTGGATCAGGCGATTGATATTCTG**NNK**GTGGCGGCGAAAC
L42X_F	TCGTTGGACCAGGCG**NNK**CGTATTCTGAGCGATGC
R43X_F	TTGGACCAGGCGCTG**NNK**ATTCTGAGCGATGCC
**Round 2 (V11R/D39H as the template)**	
L8X_F	GAGATATACATATGGTGAGCCTGGATCAGGCGATT**NNK**ATTCTGCGGGTGGC
L42X_F	TCGTTGCACCAGGCG**NNK**CGTATTCTGAGCGCG
L42X_R	CGCCTGGTGCAAC
L43X_F	TTGCACCAGGCGCTG**NNK**ATTCTGAGCGCGG
L43X_R	CAGCGCCTGGTG

Shuffling by overlap extension PCR

After each round of evolution, beneficial diversity was combined by DNA shuffling of fragments generated by overlap extension PCR. Primers were designed to encode either the parent amino acid or the identified mutation. These primers were used to generate short fragments (up to six), which were gel-purified and mixed appropriately in overlap extension PCR to generate genes containing all possible combinations of mutations. Genes were cloned as described above.

Library screening

For protein expression and screening, all transfer and aliquoting steps were performed using Hamilton liquid-handling robots. Chemically competent *E. coli* BL21 (DE3) cells were transformed with the ligated libraries described above. Freshly transformed clones were used to inoculate 150 µL of 2×YT medium supplemented with 50 µg mL^{-1} kanamycin in Corning® Costar® 96-well microtiter round bottom plates. For reference, each plate contained six freshly transformed clones of the parent template and two clones containing an empty pET29b(+) vector. Plates were incubated overnight at 30 °C, 80% humidity in a shaking incubator (Infors) at 850 r.p.m. 20 µL of overnight culture was used to inoculate 480 µL 2×YT medium supplemented with 50 µg mL^{-1} kanamycin. The cultures were incubated for ~ 2 h at 30 °C, 80% humidity with shaking at 850 r.p.m. At approximately OD_{600} = 0.5, IPTG was added to a final concentration of 0.1 mM, and plates were incubated for 20 h at 30 °C. Subsequently, cells were collected by centrifugation at 2,900 *g* for 10 min. The supernatant was discarded and the pelleted cells were re-suspended in 400 µL lysis buffer (50 mM KPi, 200 mM NaCl, pH 7.2 buffer supplemented with 1.0 mg mL^{-1} lysozyme, 0.5 mg mL^{-1} polymyxin B and 10 µg mL^{-1} DNase I) and incubated for 1 h at 30 °C, 80% humidity with shaking at 850 r.p.m., followed by a 60 °C heat shock for 1 h at 850 r.p.m.. Precipitates were removed by centrifugation at 2,900 *g* for 20 min. 20 µL of clarified lysate were transferred to Corning® Costar® 96-well microtiter round bottom plates, followed by the addition of 20 µL hemin (final assay concentration 1 µM, from a 10 µM stock in assay buffer) and incubated at room temperature for 20 min. Subsequently, 140 µL of assay buffer containing Amplex™ Red substrate (50 µM, from a 71.5 µM stock in assay buffer) was transferred to the heme loaded lysate. Reactions were initiated by the addition of 20 µL H_2O_2 (500 µM, from a 5 mM stock in assay buffer). Resorufin formation was monitored by the absorbance change at 571 nm over 20 min using a CLARIOstar plate reader (BMG Labtech).

The most active clones from each round were rescreened in lysate in triplicate. Expression and screening were performed as described above, but cultures were inoculated from glycerol stocks prepared from the original library cultures. Following each round, the most active variants were rescreened as purified proteins. Proteins were expressed and purified as described above with the exception that starter cultures were inoculated from glycerol stocks prepared from the original library plate overnight cultures.

Steady-state kinetic assays to determine the total turnover numbers

Steady-state kinetic assays were performed on a Cary UV-50 spectrophotometer (Varian) with a 1 cm path length quartz cuvette. Amplex™ Red substrate (50 µM) and enzymes (concentration of 0.1 µM for dnHEM1.2 and dnHEM1.2 H148A) were mixed in assay buffer (50 mM KPi, 200 mM NaCl, pH 7.2, total volume 1 mL). The reaction was initiated by the addition of 20 µL H_2O_2 (final concentration 500 µM) and the UV−Vis spectra at 571 nm was recorded immediately as a function of time. The absorbance of the product was converted to concentration using the extinction coefficient (ε_{571}) of 58,000 $M^{-1}cm^{-1}$. Assays were performed in triplicate.

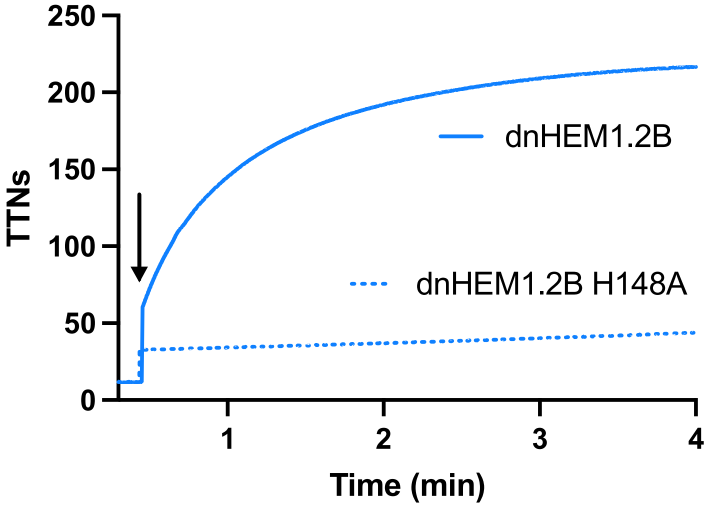

Supplementary Fig. 17. Steady-state kinetic assay of dnHEM1.2B and dnHEM1.2B H148A. The black arrow indicates the time of H_2O_2 addition. Assay conditions: enzyme (0.1 µM), Amplex^{TM} Red (50 µM), H_2O_2 (500 µM), in assay buffer at 25 °C.

<u>Enzyme kinetics</u>

Stopped-flow absorbance experiments were performed on an SX20 rapid mixing stopped-flow spectrophotometer (Applied Photophysics Ltd., Leatherhead, UK) equipped with a xenon arc lamp and a 1 cm path length in assay buffer (50 mM KPi, 200 mM NaCl, pH 7.2).

Holo dnHEM1 variants were diluted to 80 nM in assay buffer. Substrate solutions containing varying concentrations of H_2O_2 (1, 2, 4, 6, 8, 10, 15, 20, 25, 30, 40, 50, 60, 80, 100, 120, 140, 160, 200, 240 mM) and 150 µM Amplex^{TM} Red were prepared in assay buffer and used immediately after preparation. Solutions were mixed in the stopped-flow UV−Vis spectrometer equilibrated to 25 °C. Product formation was monitored by the absorbance change at 571 nm for resorufin (ε_{571} = 58,000 $M^{-1}cm^{-1}$). Three shots were taken per sample, and the averaged traces of the triplicates were fitted by linear regression (Pro-Data Viewer software). The observed initial rates were fitted to the Michaelis−Menten equation (dnHEM1 and dnHEM1.2B). The initial rates of dnHEM1.2 were fitted to the Michaelis−Menten equation with substrate inhibition (Supplementary Fig. 18a):

$$v = \frac{V_{max} \times [S]}{K_m + [S] \times \left(1 + \frac{[S]}{K_s}\right)}$$

Michaelis-Menten kinetics were measured for Amplex^{TM} Red by mixing *holo* dnHEM1 variants (final 80 nM) with H_2O_2 solution (final 5 mM) containing varying concentrations of Amplex^{TM} Red (3, 6, 12, 25, 40, 50, 75, 100, 150, 200 µM) in the stopped-flow UV−Vis spectrometer equilibrated to 25 °C. Product formation was monitored by the absorbance change at 571 nm for resorufin. Three shots were taken per sample, and the averaged traces of the triplicates were fitted by linear regression (Pro-Data Viewer software). The observed initial rates were fitted to the Michaelis−Menten equation (Supplementary Fig. 18b).

Pseudo first-order rate constants for the formation of the ferryl intermediate of dnHEM1.2B were obtained as follows. In a stopped-flow UV−Vis spectrometer equilibrated to 25 °C, one syringe containing 5 µM enzyme in assay buffer was mixed with the other syringe containing at least a 10-fold excess of H_2O_2 (50, 100, 200, and 400 µM) in the same buffer. Ferryl species formation was monitored by a decrease in absorbance at

401 nm and traces were fitted to a double exponential equation. The second-order rate constants were extracted from the slope of the plots. Reported values are the average of three individual measurements (Supplementary Fig. 19).

The decay rate of the ferryl species was determined using double mixing stopped-flow experiments according to a previous procedure.[7] To this end, 12 µM dnHEM1.2B was mixed 1:1 with 400 µM H_2O_2 in assay buffer at 25 °C. The mixture was aged until the protein reached full conversion to the ferryl state (8 s) before being mixed 1:1 with 500 nM bovine liver catalase to degrade any excess H_2O_2. Spectra were recorded with a photodiode array, the decay of the ferryl intermediate was monitored by an increase in absorbance at 401 nm, and the resulting time traces were fitted to a single exponential (Pro-Data Viewer software) to derive autoreduction rates (Supplementary Fig. 21).

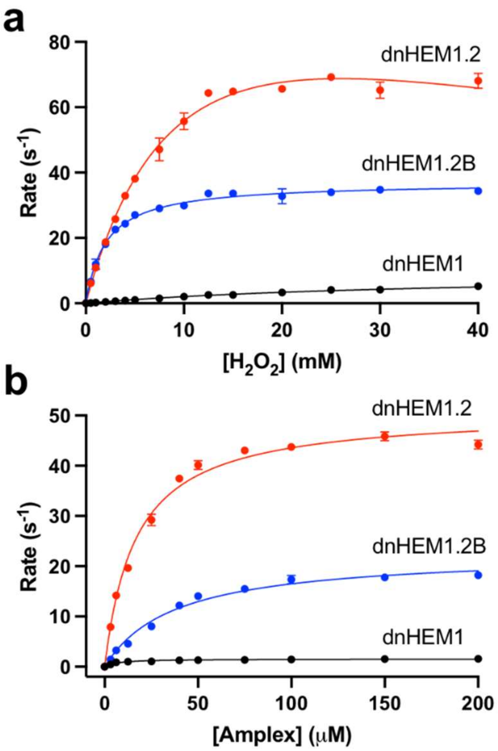

	k_{cat} (s^{-1})	K_M (mM)	k_{cat}/K_M (M^{-1}s^{-1})
dnHEM1	9.5 ± 0.2	36.7 ± 1.6	0.3 × 10^3
dnHEM1.2	129.5 ± 8.7	11.5 ± 1.2	11 × 10^3
dnHEM1.2B	37.0 ± 0.3	2.0 ± 0.1	19 × 10^3

	V_{max} / [E] at 5 mM H_2O_2 (s^{-1})	K_M (µM)	V_{max}/K_M (M^{-1}s^{-1})
dnHEM1	1.5 ± 0.04	6.9 ± 1.0	0.2 × 10^6
dnHEM1.2	50.8 ± 0.7	16.7 ± 1.0	3 × 10^6
dnHEM1.2B	22.7 ± 0.6	37.5 ± 2.9	0.6 × 10^6

Supplementary Fig. 18. (a) Michaelis-Menten analysis of the peroxidase reaction at saturating concentrations of Amplex™ Red (150 µM) for dnHEM1 and variants. Assay conditions: enzyme (80 nM), Amplex™ Red (150 µM), H_2O_2 (0-40 mM), in assay buffer (50 mM KPi, 200 mM NaCl, pH 7.2) at 25 °C. (b) Michaelis-Menten analysis of the peroxidase reaction at fixed H_2O_2 concentration (5 mM) and varying Amplex™ Red concentration for dnHEM1 variants. Assay conditions: enzyme (80 nM), Amplex™ Red (0-200 µM), H_2O_2 (5 mM), in assay buffer (50 mM KPi, 200 mM NaCl, pH 7.2) at 25 °C.

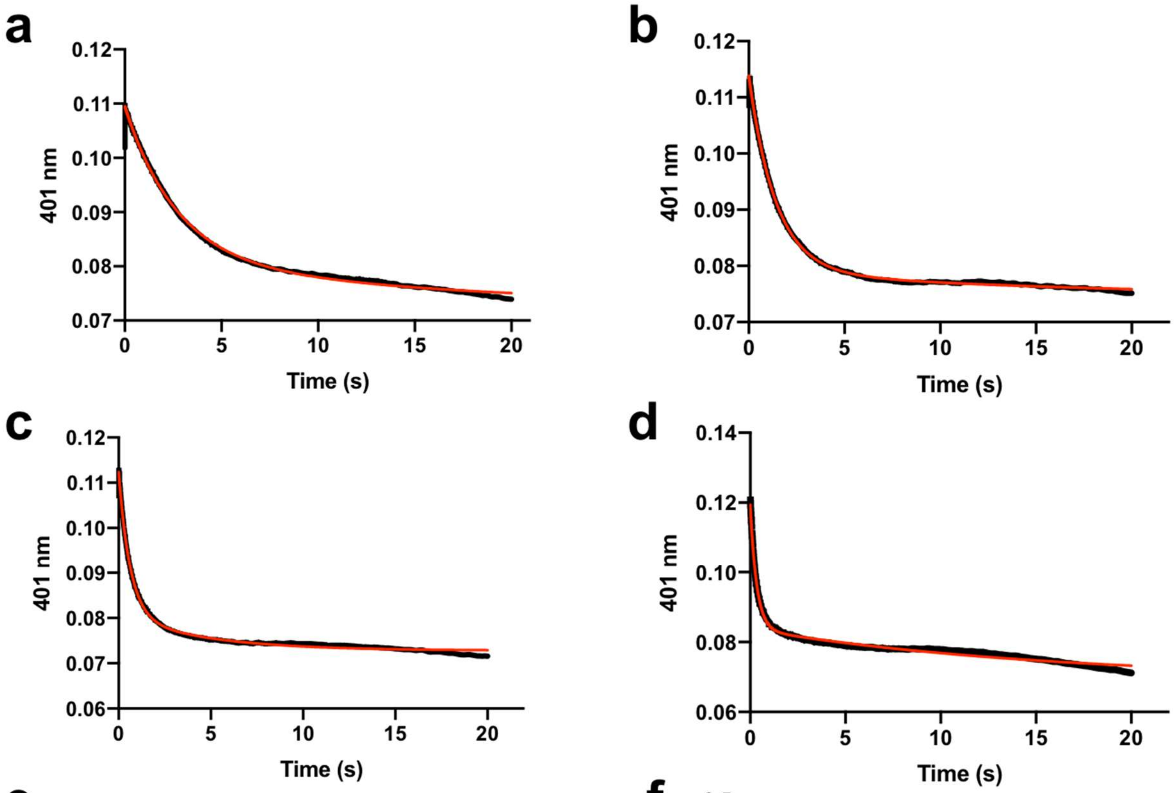

e

[H_2O_2] µM	Average (s^{-1})	SD (s^{-1})	*n*
25	0.40	0.09	3
50	0.83	0.15	3
100	1.64	0.14	3
200	3.09	0.22	3

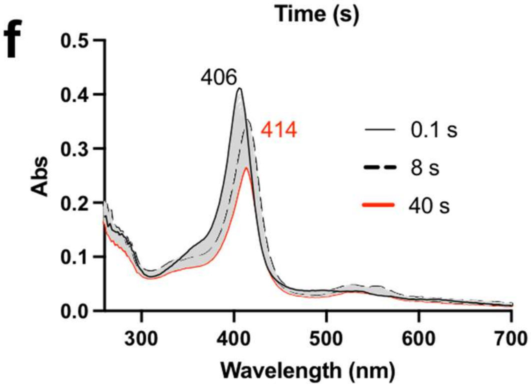

Supplementary Fig. 19. Kinetic transients for ferryl species formation at 401 nm for dnHEM1.2B. Averaged kinetic traces (*n*=3) for stopped flow reactions with (a) 25 µM, (b) 50 µM, (c) 100 µM, (d) 200 µM H_2O_2 and 2.5 µM dnHEM1.2B (black lines) (all post mixing concentrations) at pH 7.2, 25 °C. Data are fitted to a double exponential decay equation (red lines). (e) Tabulated fitted values of k_{obs} of ferryl intermediate formation at varying peroxide concentrations. Each measurement reported is an average of three repeats (*n*=3). (f) Spectra at selected time points following of mixing dnHEM1.2B (6 µM) with H_2O_2 (200 µM) in assay buffer at 25 ˚C. The Soret maximum was shifted to a longer wavelength during the first 8 s, correlating to the formation of a neutral ferryl heme species. After 8 s, the intensity of the Soret band reduces without any shift on the maximum, consistent with a slow bleaching of the heme cofactor in the presence of H_2O_2.

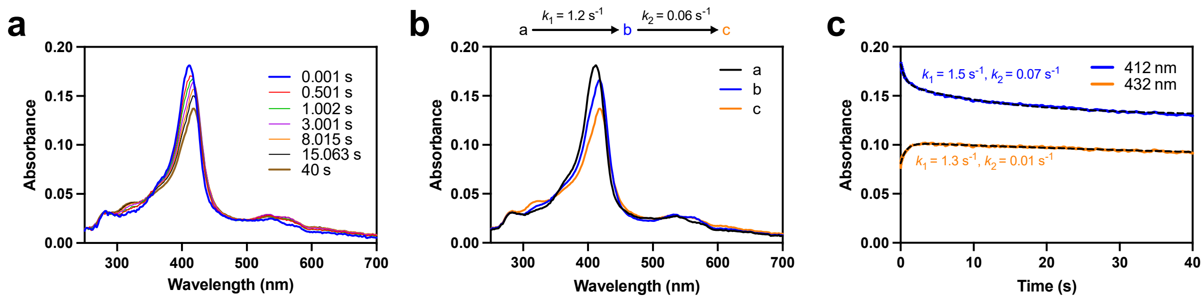

Supplementary Fig. 20. Stopped-flow kinetics for oxidation of ferric dnHEM1-V11R (2.5 μM) mixed with H_2O_2 (200 μM) in assay buffer at 25 ˚C. (a) Raw UV-visible absorbance spectra at selected time points. (b) Global fit of the raw UV-visible kinetic data using a sequential a-b-c model gives rates of $k_1 = 1.2 \pm 0.003$ s^{-1}, $k_2 = 0.06 \pm 0.0004$ s^{-1}. (c) Kinetic transients at selected wavelengths with rates, overlaid by the fits derived from the global fit (black dashed lines).

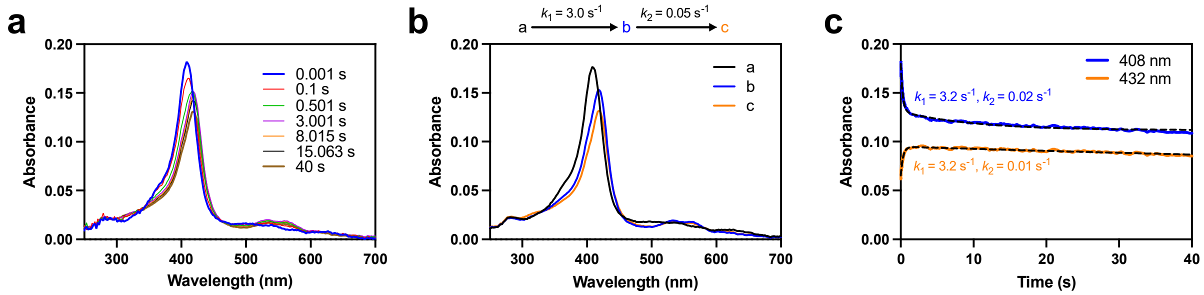

Supplementary Fig. 21. stopped-flow kinetics for oxidation of ferric dnHEM1.2B (2.5 μM) mixed with H_2O_2 (200 μM) in assay buffer at 25 ˚C. (a) Raw UV-visible absorbance spectra at selected time points. (b) Global fit of the raw UV-visible kinetic data using a sequential a-b-c model gives rates of $k_1 = 3.0 \pm 0.005$ s^{-1}, $k_2 = 0.05 \pm 0.0005$ s^{-1}. (c) Kinetic transients at selected wavelengths with rates, overlaid by the fits derived from the global fit (black dashed lines).

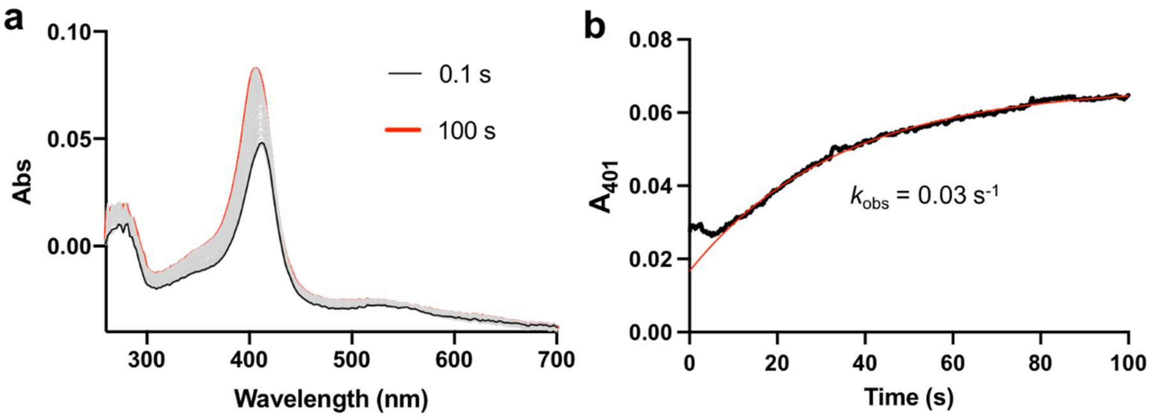

Supplementary Fig. 22. Double mixing stopped-flow experiment for the determination of ferryl species stability of dnHEM1.2B. (a) Auto-reduction of ferryl species (black) to resting state (red) over 100 s at pH 7.2, in assay buffer (50 mM KPi, 200 mM NaCl, pH 7.2) at 25 °C. (b) The absorbance change at 405 nm (black) fitted to a single exponential function (red) to give $k_{obs} = 0.03$ s^{-1}. Assay conditions: enzyme (3 μM), H_2O_2 (100 μM), catalase (250 nM).

1.4. Olefin cyclopropanation studies

Analytical scale cyclopropanation biotransformations under anaerobic conditions

Analytical scale biotransformations (400 µL) were performed in glass vials (2 mL) in a N_2 glove box. To 320 µL assay buffer (50 mM KPi, 200 mM NaCl, pH 7.2, N_2 degassed overnight), *holo* dnHEM1 (4 µL, from a 100 µM stock in assay buffer) was added, followed by styrene (10 µL, 40 mM in MeCN) and ethyl diazoacetate (EDA) (10 µL, 400 mM in MeCN). Sodium dithionite (4 µL, 10 mM stock in assay buffer) was added last. Reaction vials were sealed and incubated outside the glove box (25 °C, 200 r.p.m.) for 2 h. The final concentrations of reagents were: 1 mM styrene, 10 mM EDA, 100 µM dithionite and 1 µM heme protein.
The reactions were quenched with the addition of 30 µL HCl (3 M). 500 µL of 1 mM 1,3,5-trimethoxybenzene in ethyl acetate was added as an internal standard. Following vortexing, the top organic layer was passed through $MgSO_4$ (supported by a piece of cotton in a glass Pasteur pipette) and was analyzed by chiral and achiral GC as described below.

Chiral GC analysis

To determine the reaction enantioselectivity, chiral GC analysis was carried out using an Agilent 7890A GC system, an FID detector, and an Agilent J&W GC column (CP-Chirasil-Dex CB, 25 m x 0.25 mm, 0.25 µm film). A 1 µL sample was injected with a detector temperature 200 °C. The temperature gradient started from 80 °C, then increased to 200 °C (5 °C per min) and held for 2 min. The total run time was 30 min. The absolute configuration of the main product enantiomer was determined by comparing the main (*S*, *S*) enantiomer generated from a Mb (H64V-V68A) catalyzed biotransformation as previously reported.[8] The results of all dnHEM1 redesigns are reported in Supplementary Table 4.

Achiral GC analysis

To determine the reaction yield, achiral GC analysis was carried out using an Agilent 7890A GC system, equipped with an Agilent GC column (Vf5, 25 m x 0.25 mm, 0.25 µm film). 1 µL sample was injected with a detector temperature 250 °C. The temperature gradient started from 50 °C for 2 min, then increased to 320 °C (20 °C per min) and held for 2 min. The total run was 20 min. The product yield is calculated based on the product calibration curve (Supplementary Fig. 23b).

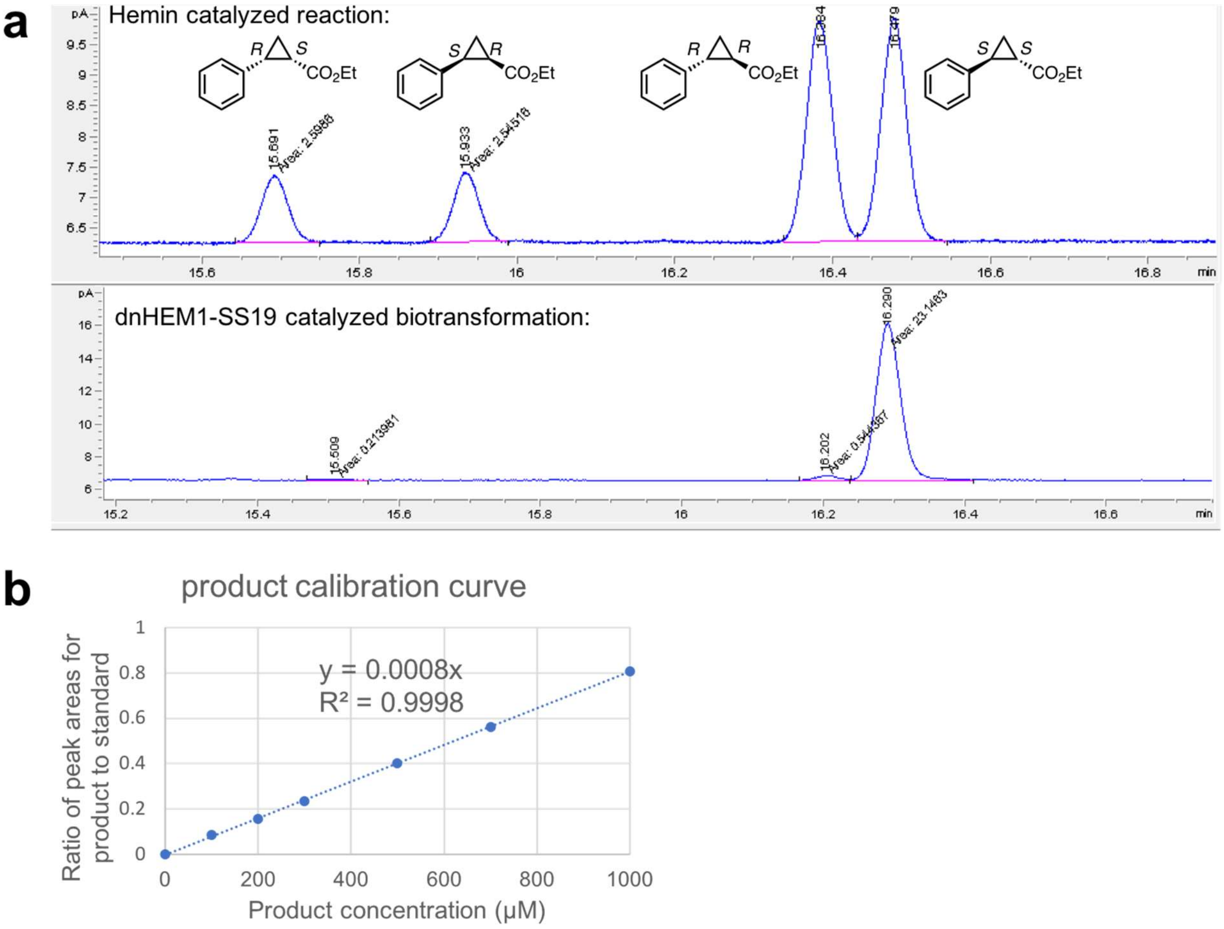

Supplementary Fig. 23. (a) Top: Chiral GC chromatogram of the cyclopropanation reaction catalyzed by hemin yields the four product enantiomers. Bottom: Chiral GC chromatogram of the cyclopropanation reaction catalyzed by dnHEM1-SS19. Standard reaction condition: 1 µM catalyst, 1 mM styrene, 10 mM EDA, 100 µM dithionite, under N_2 in aqueous potassium phosphate buffer (50 mM, NaCl 200 mM, pH 7.2) and 5% MeCN cosolvent for 2 h at 25 ˚C. (b) GC calibration curve with known commercial trans product standard using an achiral Vf5 GC column.

Supplementary Table 4. Enantioselectivity and diastereoselectivity of *holo* dnHEM1 designs for asymmetric cyclopropanation. Reaction conditions: 1 mM styrene, 10 mM EDA, 1 µM *holo* enzyme and 100 µM dithionite in assay buffer (KPi 50 mM, NaCl 200 mM, pH 7.2) at 25 °C for 2 h under anaerobic conditions. The designed mutations from the most selective *R*,*R*- and *S*,*S*-designs were transferred into the dnHEM1(pI10) scaffold with minimal changes in selectivity.

	**e.r. (trans) *R*,*R*:*S*,*S***	**d.r.**	**Designed for stereoisomer**	**Pocket mutations**
**hemin**	50:50	76:24	-	
**dnHEM1**	69:31	99.5:0.5	-	
**dnHEM1-1**	78.5:21.5	98:2	*R*,*R*	Q5L, L8V, V11I, R43I, S46A, D39F, E109L
**dnHEM1-RR2**	86:14	99:1	*R*,*R*	L8I, V11I, R43V, S46A, D39Y, E109L
**dnHEM1-RR2 (pI10)**	85:15	99:1	*R*,*R*	L8I, V11I, R43V, S46A, D39Y, E109L
**dnHEM1-3**	81.5:18.5	98.5:1.5	*R*,*R*	L8V, V11I, R43V, S46A, D39F, E109L
**dnHEM1-4**	70:30	100:0	*R*,*R*	L8I, V11I, R43V, S46A, D39F, E109L
**dnHEM1-5**	18.5:81.5	100:0	*R*,*R*	Q5F, L8V, V11I, V12L, L42A, R43V, S46L, D39E, A75T, E109L
**dnHEM1-6**	76.5:23.5	100:0	*R*,*R*	L8I, V11I, R43V, S46A, D39L, A75T, E109I
**dnHEM1-7**	11:89	98.5:1.5	*R*,*R*	Q5E, L8I, V11I, R43F, S46L, D39V, E109L
**dnHEM1-8**	66:34	97.5:2.5	*R*,*R*	L8I, V11I, R43L, S46A, D39F, A75T, E109T
**dnHEM1-9**	10:90	98:2	*R*,*R*	Q5L, L8V, V11I, R43F, S46L, D39L, E109L
**dnHEM1-10**	16:84	98.5:1.5	*R*,*R*	Q5E, L8I, V11I, R43F, S46L, D39L, E109L
**dnHEM1-11**	13.5:87.5	99.5:0.5	*R*,*R*	Q5E, L8I, V11I, R43F, S46L, D39I, E109L
**dnHEM1-12**	34.5:65.5	100:0	*R*,*R*	Q5F, L8V, V11I, L42A, R43F, S46L, D39F, E109L
**dnHEM1-13**	8.5:91.5	98:2	*S*,*S*	Q5L, V11I, V12L, L42A, R43I, S46F, D39E, E109L
**dnHEM1-14**	8.5:91.5	100:0	*S*,*S*	V11I, R43T, S46A, D39E, E109L
**dnHEM1-15**	42.5:57.5	100:0	*S*,*S*	L8I, V11I, R43T, S46A, D39L, E109L
**dnHEM1-16**	8:92	99.5:0.5	*S*,*S*	V11I, R43F, S46V, D39E, E109L
**dnHEM1-17**	4.5:95.5	99.5:0.5	*S*,*S*	Q5F, V11I, V12L, L42A, R43I, S46F, D39E, E109L
**dnHEM1-18**	40:60	100:0	*S*,*S*	Q5E, L8I, V11I, R43F, S46A, D39L, E109L
**dnHEM1-SS19**	3:97	99.5:0.5	*S*,*S*	Q5E, V11I, V12L, R43I, S46F, D39L, E109L
**dnHEM1-SS19 (pI10)**	3:97	99:1	*S*,*S*	Q5E, V11I, V12L, R43I, S46F, D39L, E109L
**dnHEM1-22**	41.5:58.5	98.5:1.5	*S*,*S*	Q5E, L8I, V11L, R43I, S46L, D39L, E109L
**dnHEM1-23**	48.5:51.3	98:2	*S*,*S*	Q5E, I7L, L8V, V11L, R43I, S46L, D39L, E109L, A183L
**dnHEM1-24**	40.5:59.5	98.5:1.5	*S*,*S*	Q5E, L8I, V11I, R43I, S46L, D39L, A75I, E109A
**dnHEM1-25**	78.5:21.5	97.5:2.5	*R*,*R*	Q5I, L8V, V11L, R43L, S46A, D39L, A75E, E109L
**dnHEM1-26**	64.5:35.5	93.5:6.5	*R*,*R*	Q5I, L8A, V11L, R43L, S46A, D39I, A75E, E109A
**dnHEM1-27**	75:25	97:3	*R*,*R*	Q5L, L8I, V11I, L42F, R43L, S46A, D39K, E109L
**dnHEM1-28**	63:37	95:5	*R*,*R*	Q5I, L8A, R43L, S46A, D39I, A75E, E109A
**dnHEM1-29**	30:70	98:2	*R*,*R*	L8V, V11I, R43L, S46L, D39L, E109V
**dnHEM1-30**	15:85	99.5:0.5	*S*,*S*	Q5I, R43L, S46A, D39I, E109L
**dnHEM1-31**	26.5:73.5	100:0	*S*,*S*	Q5E, R43L, S46A, D39L, E109A
**dnHEM1-32**	25.5:74.5	99.5:0.5	*S*,*S*	Q5I, R43V, S46A, D39L, E74F, A75E, E109A
**dnHEM1-33**	45:55	98.5:1.5	*S*,*S*	Q5I, L8I, V12A, R43I, S46L, D39I, E74F, A75E, E109A
**dnHEM1-34**	20:80	100:0	*S*,*S*	V11I, R43L, S46A, D39L, E109V
**dnHEM1-35**	13:87	99:1	*S*,*S*	Q5E, R43A, S46A, D39L, E74L, E109A
**dnHEM1-36**	9.5:90.5	100:0	*S*,*S*	Q5E, V11I, R43L, S46A, D39L, A78V, E109V

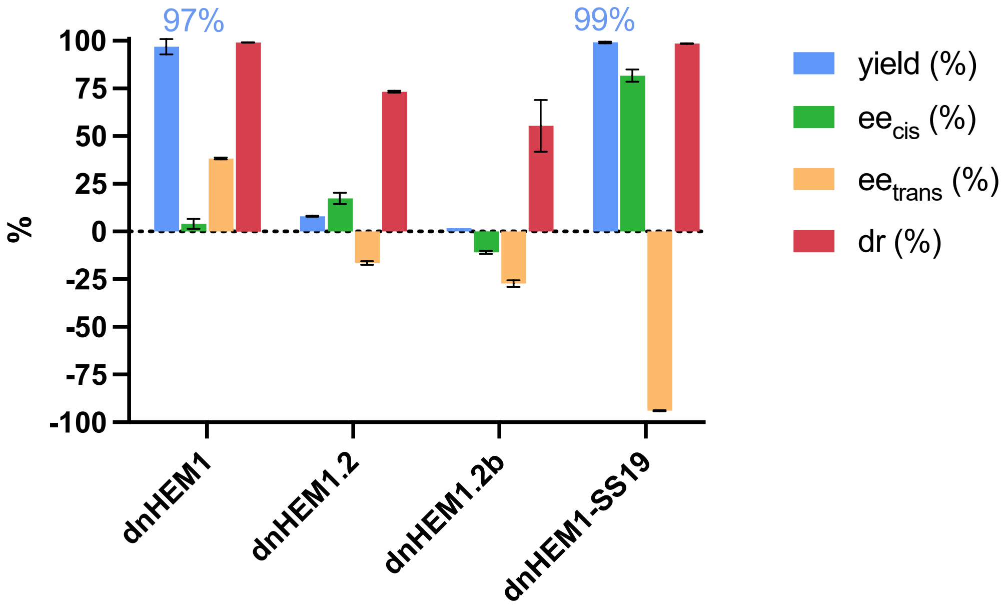

Supplementary Fig. 24. The initial design (dnHEM1) and the engineered peroxidases (dnHEM1.2 and dnHEM1.2b) are inefficient at promoting selective cyclopropanations. Reaction conditions: styrene (1 mM), EDA (10 mM), heme proteins (1 µM), dithionite (100 µM), reaction buffer KPi (50 mM, NaCl 200 mM, pH 7.2), 25 °C for 2 h.

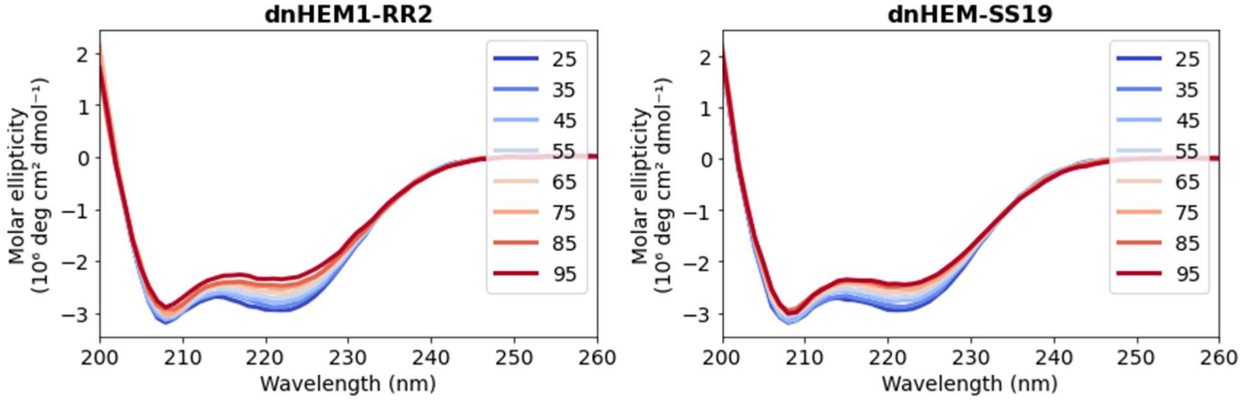

Supplementary Fig. 25. Circular dichroism (CD) spectra of *holo*-dnHEM1-RR2 and *holo*-dnHEM1-SS19 recorded at temperatures from 25 °C to 95 °C at 0.4 mg mL^{-1} protein concentration in 25 mM Tris-HCl, 30 mM NaCl buffer at pH 8.2.

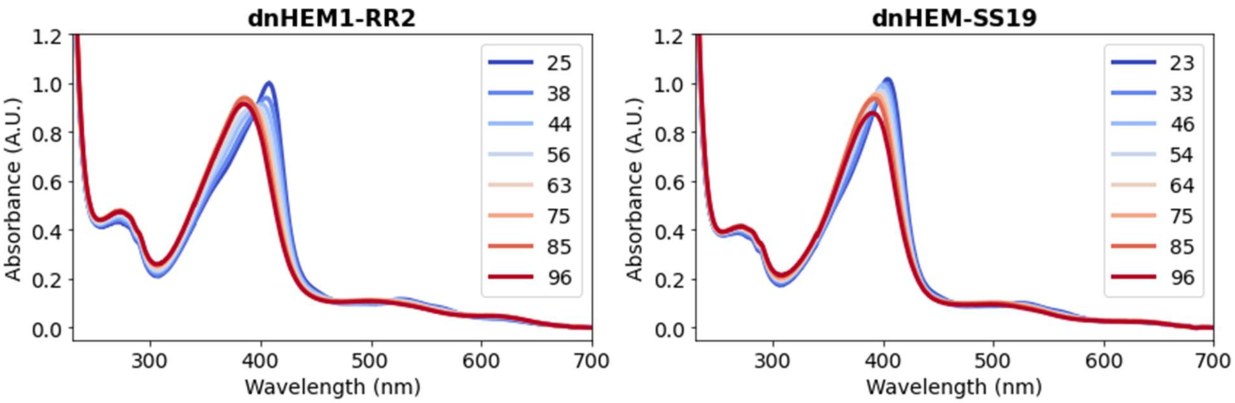

Supplementary Fig. 26. UV−Vis spectra of *holo*-dnHEM1-RR2 and *holo*-dnHEM1-SS19 recorded while increasing temperature from 25 °C to 95 °C.

<u>Preparative scale cyclopropanation under anaerobic conditions</u>

In an anaerobic N_2 glove box, *holo* dnHEM1-SS19 (167.6 µL, from a 238.7 µM stock in degassed assay buffer) was added to 37.5 mL degassed assay buffer in a 100 mL round bottom flask equipped with a stirrer bar. Styrene (1 mL, from a 200 mM stock in MeCN) was added, followed by slow addition of EDA (1 mL, from a 400 mM stock in MeCN) over 1 h by a syringe pump. Dithionite (400 µL, from a 10 mM stock in degassed assay buffer) was added, and the reaction mixture was stirred for 2 h at 25 °C. The final concentrations of reagents were: 5 mM styrene, 10 mM EDA, 100 µM dithionite and 1 µM heme protein.

The reaction was quenched inside the glove box by HCl (3 M), extracted with ethyl acetate (3 x 30 mL) in a separating funnel under ambient conditions. The organic layers were combined, dried by $MgSO_4$ and concentrated in vacuo. The resulting crude mixture was analyzed by ^{1}H NMR, then further purified by silica chromatography (diethyl ether: cyclohexane= 1: 10), resulting an isolated product yield of 93% with 93% *e.e.* for the *trans* (*S*, *S*) enantiomer.

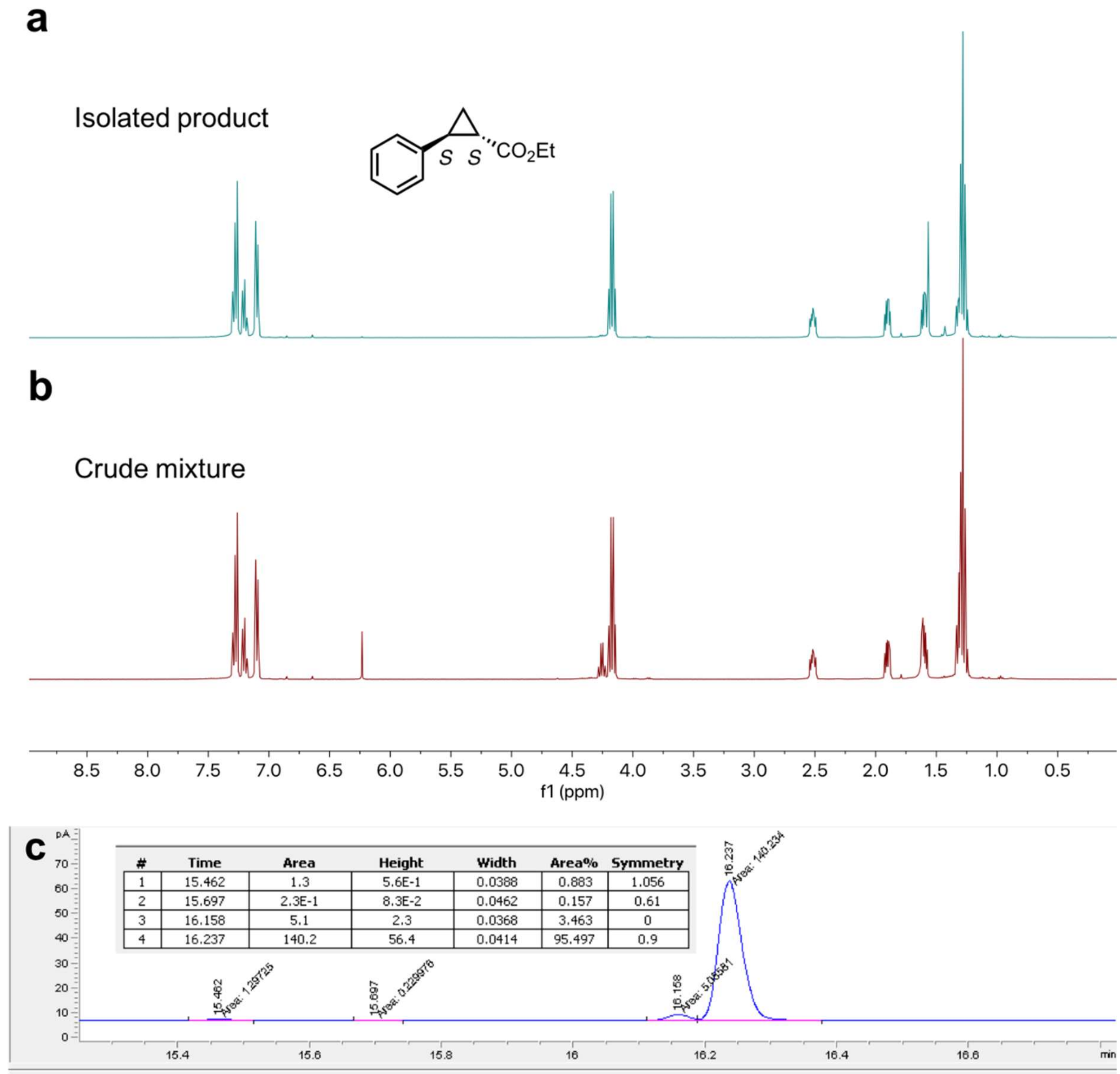

Supplementary Fig. 27. Preparative scale biotransformation for Cyclopropanation by dnHEM1-SS19. (a) ^{1}H NMR of the pure product after flash column chromatography purification. (b) Crude ^{1}H NMR of the reaction mixture after extraction. (c) Chiral GC analysis of the purified cyclopropane product (93% isolated yield, 97.5:3.5 e.r., 99:1 d.r.). Reaction condition: 1 µM catalyst, 5 mM styrene, 10 mM EDA, 100 µM dithionite, under N_2 in aqueous potassium phosphate buffer (50 mM, NaCl 200 mM, pH 7.2) and 5% MeCN cosolvent for 2 h at 25 ˚C.

Ethyl (1S,2S)-2-phenylcyclopropane-1-carboxylate

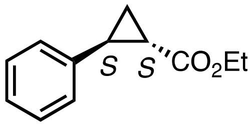

^{1}H NMR (400 MHz, $CDCl_3$) δ 7.30 – 7.26 (m, 1H, Ar*H*), 7.22 – 7.18 (m, 1H, Ar*H*), 7.11 – 7.09 (m, 2H, Ar*H*), 4.17 (q, *J* = 8 Hz, 2H, $CH_2$$CH_3$), 2.52 (ddd, *J* = 9.5, 6.5, 4.2 Hz, 1H, Ph*CH*), 1.92 – 1.88 (m, 1H, C*H*CO_2Et), 1.62 – 1.59 (m, 1H, CH*H*), 1.33 – 1.26 (m, 4H, C*H*H and $CH_2$$CH_3$).

1.5. Amino acid and DNA sequences of de novo designed heme binding proteins

Supplementary Table 5. Sequences of de novo heme binding proteins.

**dnHEM1**	
AA sequence:	MVSLDQAILILVVAAKLGTTVEEAVKRALWLKTKLGVSLDQALRILSAAANTGTTVEEAVKRALKLKTKLGVSLEAALAILSAAAQLGTT<br>VEEAVKRALKLKTKLGVDLETAALALLTAAKLGTTVEEAVKRALKLKTKLGVSLIEAL**H**ILLTAAVLGTTVEEAVYRALKLKTKLGVSLL<br>QAAAILILAARLGTTVEEAVKRALKLKTKLGGGSGG*SHHWGSGSHHHHHH*
DNA sequence:	GTGAGCCTGGATCAGGCGATTCTGATTCTGGTGGTGGCGGCGAAACTGGGCACCACCGTGGAAGAAGCGGTGAAACGCGCGCTGTGGCTG<br>AAAACCAAATTAGGCGTGTCGTTGGACCAGGCGCTGCGTATTCTGAGCGCGGCCGCCAATACCGGCACGACGGTTGAAGAGGCCGTTAAA<br>CGTGCACTGAAACTGAAGACGAAGTTGGGTGTTTCGCTGGAAGCGGCGCTGGCGATTTTAAGCGCCGCGGCGCAGCTGGGTACGACCGTT<br>GAGGAGGCGGTTAAGCGCGCGTTGAAATTGAAAACGAAATTGGGGGTGGATCTGGAAACCGCGGCCCTGGCGTTGTTGACCGCAGCCAAG<br>CTCGGTACCACTGTGGAGGAAGCAGTCAAGCGTGCCCTGAAGTTAAAGACCAAGCTGGGGGTCAGCTTGATTGAGGCACTGCATATTCTG<br>CTGACCGCTGCGGTGTTGGGCACTACCGTAGAGGAAGCAGTGTATCGCGCCTTGAAGCTCAAGACTAAGTTAGGTGTTAGTCTGCTGCAG<br>GCGGCAGCCATCTTGATTTTAGCCGCGCGCCTGGGGACGACTGTCGAAGAGGCTGTGAAGCGCGCGCTCAAGTTGAAGACCAAACTCGGT<br>GGCGGGAGCGGTGGCTCTCATCATTGGGGCAGTGGCTCGCATCATCACCACCATCAT
**dnHEM1_H148A**	
AA sequence:	MVSLDQAILILVVAAKLGTTVEEAVKRALWLKTKLGVSLDQALRILSAAANTGTTVEEAVKRALKLKTKLGVSLEAALAILSAAAQLGTT<br>VEEAVKRALKLKTKLGVDLETAALALLTAAKLGTTVEEAVKRALKLKTKLGVSLIEAL**A**ILLTAAVLGTTVEEAVYRALKLKTKLGVSLL<br>QAAAILILAARLGTTVEEAVKRALKLKTKLGGGSGG*SHHWGSGSLEHHHHHH*
DNA sequence:	ATGGTGAGCCTGGATCAGGCGATTCTGATTCTGGTGGTGGCGGCGAAACTGGGCACCACCGTGGAAGAAGCGGTGAAACGCGCGCTGTGG<br>CTGAAAACCAAATTAGGCGTGTCGTTGGACCAGGCGCTGCGTATTCTGAGCGCGGCCGCCAATACCGGCACGACGGTTGAAGAGGCCGTT<br>AAACGTGCGCTGAAACTGAAGACGAAGTTGGGTGTTTCGCTGGAAGCGGCGCTGGCGATTTTAAGCGCCGCGGCGCAGCTGGGTACGACC<br>GTTGAGGAGGCTGTGAAGCGCGCGTTGAAATTGAAAACGAAATTGGGGGTGGATCTGGAAACCGCGGCCCTGGCGTTGTTGACCGCAGCC<br>AAGCTCGGTACCACGGTCGAGGAAGCAGTGAAGCGCGCCCTTAAGTTGAAGACTAAACTGGGCGTGAGCTTGATTGAGGCGTTGGCCATT<br>CTGCTGACTGCTGCGGTGCTGGGGACCACTGTAGAAGAGGCAGTGTATCGCGCCTTGAAGTTAAAGACCAAGTTAGGTGTTAGTCTGCTG<br>CAGGCGGCAGCCATCTTGATTTTAGCCGCGCGCCTGGGCACTACTGTGGAGGAGGCTGTCAAGCGTGCACTGAAATTAAAAACGAAGCTG<br>GGTGGCGGGAGCGGTGGCTCCCATCATTGGGGCAGCGGCAGCCTCGAGCACCACCACCACCACCAC
**dnHEM1_H148F**	
AA sequence:	MVSLDQAILILVVAAKLGTTVEEAVKRALWLKTKLGVSLDQALRILSAAANTGTTVEEAVKRALKLKTKLGVSLEAALAILSAAAQLGTT<br>VEEAVKRALKLKTKLGVDLETAALALLTAAKLGTTVEEAVKRALKLKTKLGVSLIEAL**F**ILLTAAVLGTTVEEAVYRALKLKTKLGVSLL<br>QAAAILILAARLGTTVEEAVKRALKLKTKLGGGSGG*SHHWGSGSLEHHHHHH*
DNA sequence:	ATGGTGAGCCTGGATCAGGCGATTCTGATTCTGGTGGTGGCGGCGAAACTGGGCACCACCGTGGAAGAAGCGGTGAAACGCGCGCTGTGG<br>CTGAAAACCAAATTAGGCGTGTCGTTGGACCAGGCGCTGCGTATTCTGAGCGCGGCCGCCAATACCGGCACGACGGTTGAAGAGGCCGTT<br>AAACGTGCACTGAAACTGAAGACCAAGCTCGGCGTTTCGCTGGAAGCGGCGCTGGCGATTTTAAGCGCTGCAGCGCAGCTGGGTACTACT<br>GTCGAGGAGGCGGTTAAGCGCGCGTTGAAATTGAAAACGAAGTTGGGTGTGGATCTGGAAACCGCGGCCCTGGCGTTGTTGACCGCAGCC<br>AAGTTAGGGACGACCGTTGAGGAAGCTGTGAAGCGTGCCTTAAAGTTAAAGACTAAGCTTGGTGTCAGCTTGATTGAGGCCCTGTTTATT<br>CTGCTGACCGCCGCGGTGCTGGGGACCACTGTGGAGGAGGCAGTGTATCGCGCCCTCAAGCTCAAAACTAAACTGGGTGTTAGCCTGCTG<br>CAGGCGGCGGCCATCTTGATTTTAGCCGCGCGTCTGGGGACGACGGTCGAAGAGGCCGTGAAGCGCGCATTGAAGCTTAAGACGAAGCTG<br>GGTGGCGGGAGCGGTGGCTCCCATCATTGGGGCAGCGGCAGCCTCGAGCACCACCACCACCACCAC
**dnHEM1_pI6**	
AA sequence:	MVSLDQAILILVVAAKLGTTVEEAV**Q**RALWLKTKLGVSLDQALRILSAAANTGTTVEEAV**QE**AL**Q**LKTKLGVSLEAALAILSAAAQLGTT<br>VEEAV**N**RAL**N**LKTKLGVDLETAALALLTAAKLGTTVEEAV**QE**AL**Q**LKTKLGVSLIEAL**H**ILLTAAVLGTTVEEAVYRALKLKTKLGVSLL<br>QAAAILILAARLGTTVEEAV**QE**AL**Q**LKTKLGGGSGG*SHHWGSGSLEHHHHHH*
DNA sequence:	ATGGTGAGCCTGGATCAGGCGATTCTGATTCTGGTGGTGGCGGCGAAACTGGGCACCACCGTGGAAGAAGCGGTGCAGCGCGCGCTGTGG<br>CTGAAAACCAAATTAGGCGTGTCTTTGGACCAGGCGTTGCGTATTTTGAGCGCGGCCGCCAATACCGGTACCACGGTTGAAGAGGCCGTT<br>CAAGAGGCGCTGCAATTGAAAACGAAGTTGGGCGTGAGCTTGGAAGCGGCGTTGGCGATTTTAAGCGCCGCGGCGCAACTGGGTACGACC<br>GTCGAGGAGGCAGTTAATCGCGCGTTGAATTTGAAGACGAAACTCGGGGTGGATCTGGAAACCGCAGCGCTGGCGTTGTTGACCGCCGCA<br>AAGCTGGGTACTACCGTTGAGGAAGCCGTCCAGGAAGCATTGCAATTAAAGACTAAATTGGGTGTTAGCTTGATTGAAGCCTTGCATATT<br>CTGCTGACTGCCGCGGTGCTGGGGACTACTGTCGAAGAGGCGGTGTATCGTGCGCTGAAACTGAAGACCAAGTTAGGTGTAAGCCTGCTG<br>CAGGCGGCGGCCATCTTGATTTTAGCGGCGCGCCTGGGTACGACGGTTGAGGAAGCCGTCCAAGAAGCGTTACAGTTGAAGACTAAGCTG<br>GGTGGCGGGAGCGGTGGCTCCCATCATTGGGGCAGCGGCAGCCTCGAGCACCACCACCACCACCAC
**dnHEM1.2**	
AA sequence:	MVSLDQAI**D**ILVVAAKLGTTVEEAVKRALWLKTKLGVSLDQALRILS**D**AANTGTTVEEAVKRALKLKTKLGVSLEAALAILSAAAQLGTT<br>VEEAVKRALKLKTKLGVDLETAALALLTAAKLGTTVEEAVKRALKLKTKLGVSLIEAL**H**ILLTAAVLGTTVEEAVYRALKLKTKLGVSLL<br>QAAAIL**L**LAARLGTTVEEAVKRALKLKTKLGGGSGGSHHWGSGSHHHHHH
DNA sequence:	ATGGTGAGCCTGGATCAGGCGATTGATATTCTGGTGGTGGCGGCGAAACTGGGCACCACCGTGGAAGAAGCGGTGAAACGCGCGCTGTGG<br>CTGAAAACCAAATTAGGCGTGTCGTTGGACCAGGCGCTGCGTATTCTGAGCGATGCCGCCAATACCGGCACGACGGTTGAAGAGGCCGTT<br>AAACGTGCACTGAAACTGAAGACGAAGCTCGGTGTTTCGTTAGAGGCGGCGCTGGCGATTTTAAGCGCAGCCGCGCAGCTGGGTACTACT<br>GTGGAGGAGGCGGTTAAGCGCGCGTTGAAATTGAAAACGAAGTTGGGCGTGGATCTGGAAACCGCGGCCTTAGCGTTGTTGACCGCAGCC<br>AAGTTAGGTACGACCGTTGAGGAAGCAGTTAAGCGCGCCCTGAAGTTAAAGACCAAGTTGGGTGTGAGCTTGATTGAGGCACTGCATATT<br>CTGCTGACTGCCGCGGTGTTAGGCACTACCGTCGAAGAGGCGGTGTATCGCGCCTTGAAGTTGAAAACTAAATTGGGGGTTAGTCTGCTG<br>CAGGCGGCTGCCATCTTGCTTTTAGCAGCCCGCCTGGGGACTACGGTGGAGGAGGCCGTAAAGCGTGCCTTAAAATTAAAAACCAAATTG<br>GGTGGGGGCAGCGGTGGCAGCCATCATTGGGGCTCGGGCTCGCATCATCACCACCATCAT
**dnHEM1.2B**	
AA sequence:	MVSLDQAILIL**R**VAAKLGTTVEEAVKRALWLKTKLGVSL**H**QALRILS**R**AANTGTTVEEAVKRALKLKTKLGVSLEAALAILSAAAQLGTT<br>VEEAVKRALKLKTKLGVDLETAALALLTAAKLGTTVEEAVKRALKLKTKLGVSLIEAL**H**ILLTAAVLGTTVEEAVYRALKLKTKLGVSLL<br>QAAAILILAARLGTTVEEAVKRALKLKTKLGGGSGGSHHWGSGSHHHHHH

DNA sequence:	ATGGTGAGCCTGGATCAGGCGATTCTGATTCTGCGGGTGGCGGCGAAACTGGGCACCACCGTGGAAGAAGCGGTGAAACGCGCGCTGTGG<br>CTGAAAACCAAATTAGGCGTGTCGTTGCACCAGGCGCTGCGTATTCTGAGCCGGGCCGCCAATACCGGCACGACGGTTGAAGAGGCCGTT<br>AAACGTGCACTGAAACTGAAGACGAAGCTCGGTGTTTCGTTAGAGGCGGCGCTGGCGATTTTAAGCGCAGCCGCGCAGCTGGGTACTACT<br>GTGGAGGAGGCGGTTAAGCGCGCGTTGAAATTGAAAACGAAGTTGGGCGTGGATCTGGAAACCGCGGCCTTAGCGTTGTTGACCGCAGCC<br>AAGTTAGGTACGACCGTTGAGGAAGCAGTTAAGCGCGCCCTGAAGTTAAAGACCAAGTTGGGTGTGAGCTTGATTGAGGCACTGCATATT<br>CTGCTGACTGCCGCGGTGTTAGGCACTACCGTCGAAGAGGCGGTGTATCGCGCCTTGAAGTTGAAAACTAAATTGGGGGTTAGTCTGCTG<br>CAGGCGGCTGCCATCTTGATTTTAGCAGCCCGCCTGGGGACTACGGTGGAGGAGGCCGTAAAGCGTGCCTTAAAATTAAAAACCAAATTG<br>GGTGGGGGCAGCGGTGGCAGCCATCATTGGGGCTCGGGCTCGCATCATCACCACCATCAT
**dnHEM1.2_H148A**	
AA sequence:	MVSLDQAI**D**ILVVAAKLGTTVEEAVKRALWLKTKLGVSLDQALRILS**D**AANTGTTVEEAVKRALKLKTKLGVSLEAALAILSAAAQLGTT<br>VEEAVKRALKLKTKLGVDLETAALALLTAAKLGTTVEEAVKRALKLKTKLGVSLIEAL**A**ILLTAAVLGTTVEEAVYRALKLKTKLGVSLL<br>QAAAIL**L**LAARLGTTVEEAVKRALKLKTKLGGGSGGSHHWGSGSHHHHHH
DNA sequence:	ATGGTGAGCCTGGATCAGGCGATTGATATTCTGGTGGTGGCGGCGAAACTGGGCACCACCGTGGAAGAAGCGGTGAAACGCGCGCTGTGG<br>CTGAAAACCAAATTAGGCGTGTCGTTGGACCAGGCGCTGCGTATTCTGAGCGATGCCGCCAATACCGGCACGACGGTTGAAGAGGCCGTT<br>AAACGTGCACTGAAACTGAAGACGAAGCTCGGTGTTTCGTTAGAGGCGGCGCTGGCGATTTTAAGCGCAGCCGCGCAGCTGGGTACTACT<br>GTGGAGGAGGCGGTTAAGCGCGCGTTGAAATTGAAAACGAAGTTGGGCGTGGATCTGGAAACCGCGGCCTTAGCGTTGTTGACCGCAGCC<br>AAGTTAGGTACGACCGTTGAGGAAGCAGTTAAGCGCGCCCTGAAGTTAAAGACCAAGTTGGGTGTGAGCTTGATTGAGGCACTGGCTATT<br>CTGCTGACTGCCGCGGTGTTAGGCACTACCGTCGAAGAGGCGGTGTATCGCGCCTTGAAGTTGAAAACTAAATTGGGGGTTAGTCTGCTG<br>CAGGCGGCTGCCATCTTGCTTTTAGCAGCCCGCCTGGGGACTACGGTGGAGGAGGCCGTAAAGCGTGCCTTAAAATTAAAAACCAAATTG<br>GGTGGGGGCAGCGGTGGCAGCCATCATTGGGGCTCGGGCTCGCATCATCACCACCATCAT
**dnHEM1-RR1**	
AA sequence:	MVSLDLAIVILIVAAKLGTTVEEAVEQALWLKTKLGVSLFQALIILAAAANTGTTVEEAVQEALQLKTKLGVSLEAALAILSAAAQLGTT<br>VEEAVNRALNLKTKLGVDLLTAALALLTAAKLGTTVEEAVQEALQLKTKLGVSLIEAL**H**ILLTAAVLGTTVEEAVYRALKLKTKLGVSLL<br>QAAAILILAARLGTTVEQAVERALQLKTKLGGGSGG*SHHWGSGSLEHHHHHH*
DNA sequence:	ATGGTGAGCCTGGATCTGGCGATTGTGATTCTGATTGTGGCGGCGAAACTGGGCACCACCGTGGAAGAAGCGGTGGAACAGGCGCTGTGG<br>CTGAAAACCAAATTAGGCGTGAGTCTGTTTCAGGCACTGATTATTCTGGCCGCCGCTGCCAATACCGGCACGACGGTAGAGGAGGCGGTG<br>CAGGAAGCGCTGCAGTTGAAGACGAAGTTGGGTGTTTCGTTGGAAGCGGCGTTAGCGATTTTAAGCGCGGCAGCGCAACTGGGTACCACG<br>GTTGAGGAAGCCGTTAATCGCGCGTTGAATTTGAAAACGAAATTGGGCGTGGATTTGTTAACCGCGGCTTTAGCCCTGCTGACCGCAGCT<br>AAACTAGGCACTACAGTCGAGGAAGCTGTACAGGAGGCTTTGCAGCTTAAAACTAAACTAGGCGTTTCACTGATAGAAGCATTGCATATC<br>CTGTTGACGGCCGCAGTACTGGGGACAACTGTAGAAGAGGCTGTATATCGAGCGCTTAAACTCAAGACAAAGCTAGGGGTTAGTTTGCTC<br>CAGGCAGCGGCCATATTGATTTTAGCAGCGCGCCTGGGGACTACAGTAGAGCAGGCTGTAGAACGCGCATTGCAACTAAAGACTAAGCTT<br>GGTGGCGGGAGCGGTGGCTCCCATCATTGGGGCAGCGGCAGCCTCGAGCACCACCACCACCACCAC
**dnHEM1-RR2**	
AA sequence:	MVSLDQAIIIILIVAAKLGTTVEEAVEQALWLKTKLGVSLYQALVILAAAANTGTTVEEAVQEALQLKTKLGVSLEAALAILSAAAQLGTT<br>VEEAVNRALNLKTKLGVDLLTAALALLTAAKLGTTVEEAVQEALQLKTKLGVSLIEAL**H**ILLTAAVLGTTVEEAVYRALKLKTKLGVSLL<br>QAAAILILAARLGTTVEEAVERALQLKTKLGGGSGG*SHHWGSGSLEHHHHHH*
DNA sequence:	ATGGTGAGCCTGGATCAGGCGATTATTATTCTGATTGTGGCGGCGAAACTGGGCACCACCGTGGAAGAAGCGGTGGAACAGGCGCTGTGG<br>CTGAAAACCAAATTAGGCGTGAGTCTGTATCAAGCACTGGTGATTCTGGCCGCCGCTGCCAATACCGGCACGACGGTTGAGGAGGCGGTG<br>CAGGAAGCGCTGCAGTTGAAAACGAAGTTGGGTGTGAGCTTGGAAGCGGCGTTGGCGATTTTAAGCGCGGCGGCGCAACTGGGTACCACG<br>GTCGAAGAGGCCGTTAATCGCGCGTTGAATTTGAAGACGAAACTCGGCGTGGATTTGTTAACCGCGGCTTTAGCCCTGCTGACCGCCGCC<br>AAGCTCGGTACGACCGTAGAAGAGGCTGTTCAAGAAGCTCTGCAATTAAAAACTAAATTAGGCGTAAGCTTGATTGAAGCCTTGCATATT<br>CTGTTGACGGCCGCGGTGCTGGGGACCACTGTCGAAGAAGCAGTGTATCGTGCGCTGAAACTGAAGACCAAGCTGGGGGTCAGTCTGCTG<br>CAGGCAGCGGCGATCTTGATTTTAGCGGCGCGCCTGGGCACCACTGTGGAGGAGGCTGTTGAACGCGCATTGCAATTGAAGACTAAACTC<br>GGTGGCGGGAGCGGTGGCTCCCATCATTGGGGCAGCGGCAGCCTCGAGCACCACCACCACCACCAC
**dnHEM1-RR2_pI10**	
AA sequence:	MSGVSLDQAIIIILIVAAKLGTTVEEAVKRALWLKTKLGVSLYQALVILAAAANTGTTVEEAVKRALKLKTKLGVSLEAALAILSAAAQLG<br>TTVEEAVKRALKLKTKLGVDLLTAALALLTAAKLGTTVEEAVKRALKLKTKLGVSLIEAL**H**ILLTAAVLGTTVEEAVYRALKLKTKLGVS<br>LLQAAAILILAARLGTTVEEAVKRALKLKTKLGGGSG*SHHWGSTHHHHHH*
DNA sequence:	ATGTCAGGAGTGAGCCTGGATCAGGCGATTATTATTCTGATTGTGGCGGCGAAACTGGGCACCACCGTGGAAGAAGCGGTGAAACGCGCG<br>CTGTGGCTGAAAACCAAATTAGGCGTGTCGTTGTATCAGGCGCTGGTGATTCTGGCGGCGGCCGCCAATACCGGCACGACGGTTGAAGAG<br>GCCGTTAAACGTGCACTGAAACTGAAGACGAAGTTGGGTGTTTCGCTGGAAGCGGCGCTGGCGATTTTAAGCGCCGCGGCGCAGCTGGGT<br>ACGACCGTTGAGGAGGCGGTTAAGCGCGCGTTGAAATTGAAAACGAAATTGGGGGTGGATCTGCTGACCGCGGCCCTGGCGTTGTTGACC<br>GCAGCCAAGCTCGGTACCACTGTGGAGGAAGCAGTCAAGCGTGCCCTGAAGTTAAAGACCAAGCTGGGGGTCAGCTTGATTGAGGCACTG<br>CATATTCTGCTGACCGCTGCGGTGTTGGGCACTACCGTAGAGGAAGCAGTGTATCGCGCCTTGAAGCTCAAGACTAAGTTAGGTGTTAGT<br>CTGCTGCAGGCGGCAGCCATCTTGATTTTAGCCGCGCGCCTGGGGACGACTGTCGAAGAGGCTGTGAAGCGCGCGCTCAAGTTGAAGACC<br>AAACTCGGTGGCGGTTCCGGCAGCCATCATTGGGGCAGCACCCACCACCACCACCACCAC
**dnHEM1-RR3**	
AA sequence:	MVSLDQAIVILIVAAKLGTTVEEAVEQALWLKTKLGVSLFQALVILAAAANTGTTVEEAVQEALQLKTKLGVSLEAALAILSAAAQLGTT<br>VEEAVNRALNLKTKLGVDLLTAALALLTAAKLGTTVEEAVQEALQLKTKLGVSLIEAL**H**ILLTAAVLGTTVEEAVYRALKLKTKLGVSLL<br>QAAAILILAARLGTTVEEAVERALQLKTKLGGGSGG*SHHWGSGSLEHHHHHH*
DNA sequence:	ATGGTGAGCCTGGATCAGGCGATTGTGATTTTAATTGTTGCGGCGAAATTAGGTACCACCGTTGAAGAAGCGGTTGAACAAGCGTTATGG<br>TTAAAAACCAAATTAGGTGTTAGCTTATTTCAAGCATTAGTTATTTTAGCAGCAGCTGCAAATACCGGTACTACTGTTGAGGAGGCAGTT<br>CAAGAGGCGTTGCAATTAAAGACTAAACTCGGCGTAAGCTTAGAGGCGGCGTTGGCGATCTTAAGCGCGGCGGCGCAACTTGGGACGACT<br>GTCGAAGAGGCCGTTAATCGTGCGTTGAATTTGAAGACCAAGCTGGGGGTGGATTTGTTAACCGCGGCCCTGGCGCTGCTGACCGCAGCT<br>AAGTTAGGCACTACCGTAGAGGAAGCCGTTCAGGAGGCCTTGCAACTGAAGACGAAACTGGGCGTTTCGTTGATTGAAGCTTTGCATATT<br>CTGTTGACGGCCGCGGTGCTGGGCACGACCGTGGAGGAGGCAGTGTATCGCGCGCTGAAACTTAAAACGAAGTTAGGTGTGAGTCTGCTG<br>CAGGCGGCCGCCATCTTGATCTTGGCCGCGCGCCTGGGGACCACGGTGGAAGAGGCTGTAGAACGCGCATTGCAGTTGAAAACTAAACTT<br>GGTGGTGGCAGCGGCGGGCTCGAGCACCACCACCACCACCAC

**dnHEM1-RR4**	
AA sequence:	MVSLDQAIIILIVAAKLGTTVEEAVEQALWLKTKLGVSLFQALVILAAAANTGTTVEEAVQEALQLKTKLGVSLEAALAILSAAAQLGTT<br>VEEAVNRALNLKTKLGVDLLTAALALLTAAKLGTTVEEAVQEALQLKTKLGVSLIEAL**H**ILLTAAVLGTTVEEAVYRALKLKTKLGVSLL<br>QAAAILILAARLGTTVEEAVERALQLKTKLGGGSGG*SHHWGSGSLEHHHHHH*
DNA sequence:	ATGGTGAGCCTGGATCAGGCGATTATTATTCTGATTGTGGCGGCGAAATTAGGTACCACCGTTGAAGAAGCGGTTGAACAAGCGTTATGG<br>TTAAAAACCAAATTAGGTGTTAGTTTATTTCAAGCATTAGTTATTTTAGCAGCAGCTGCAAATACCGGTACTACTGTTGAGGAGGCAGTT<br>CAAGAGGCGTTGCAATTAAAGACTAAACTCGGCGTCAGCTTAGAGGCGGCGTTGGCGATTTTAAGCGCGGCAGCGCAACTGGGCACGACT<br>GTAGAAGAGGCGGTTAATCGTGCGTTGAATTTAAAGACGAAGTTGGGGGTGGATTTATTAACCGCGGCCCTGGCGCTGCTGACCGCAGCC<br>AAGCTGGGTACGACCGTCGAAGAGGCTGTTCAGGAGGCCTTGCAACTGAAGACCAAGTTAGGCGTTAGCTTGATTGAAGCCCTGCATATT<br>CTGTTGACGGCCGCGGTGCTGGGGACGACGGTAGAGGAGGCCGTGTATCGCGCGCTGAAATTAAAGACGAAGCTTGGTGTAAGCTTATTA<br>CAAGCGGCTGCAATTTTAATTCTCGCGGCGCGTCTGGGGACGACGGTAGAGGAAGCCGTAGAACGCGCATTGCAGTTGAAAACTAAACTG<br>GGTGGTGGCAGCGGGGGCCTCGAGCACCACCACCACCACCAC
**dnHEM1-RR5**	
AA sequence:	MVSLDFAIVILILAAKLGTTVEEAVEQALWLKTKLGVSLEQAAVILLAAANTGTTVEEAVQEALQLKTKLGVSLETALAILSAAAQLGTT<br>VEEAVNRALNLKTKLGVDLLTAALALLTAAKLGTTVEEAVQEALQLKTKLGVSLIEAL**H**ILLTAAVLGTTVEEAVYRALKLKTKLGVSLL<br>QAAAILILAARLGTTVEQAVERALQLKTKLGGGSGG*SHHWGSGSLEHHHHHH*
DNA sequence:	ATGGTGAGCCTGGATTTTGCGATTGTGATTCTGATCCTGGCGGCGAAACTGGGCACCACCGTGGAAGAAGCGGTGGAACAGGCGCTGTGG<br>CTGAAAACCAAATTAGGCGTGTCTTTGGAACAGGCGGCGGTGATTCTGCTGGCTGCCGCCAATACCGGCACGACGGTTGAAGAGGCCGTT<br>CAAGAAGCCCTGCAATTGAAAACGAAGTTGGGTGTCAGCTTGGAAACCGCGCTGGCGATTTTAAGCGCGGCGGCGCAGCTGGGTACGACC<br>GTCGAGGAGGCAGTGAATCGCGCGCTGAATCTGAAGACCAAGCTGGGGGTGGATTTGTTAACCGCGGCTTTAGCCCTGCTGACCGCAGCT<br>AAACTAGGCACTACAGTCGAGGAAGCTGTACAGGAGGCTTTGCAGCTTAAAACTAAACTAGGCGTTTCACTGATAGAAGCATTGCATATC<br>CTGTTGACGGCCGCAGTACTGGGGACAACTGTAGAAGAGGCTGTATATCGAGCGCTTAAACTCAAGACAAAGCTAGGGGTTAGTTTGCTC<br>CAGGCAGCGGCCATATTGATTTTAGCAGCGCGCCTGGGGACTACAGTAGAGCAGGCTGTAGAACGCGCATTGCAACTAAAGACTAAGCTT<br>GGTGGCGGGAGCGGTGGCTCCCATCATTGGGGCAGCGGCAGCCTCGAGCACCACCACCACCACCAC
**dnHEM1-RR6**	
AA sequence:	MVSLDQAIIILIVAAKLGTTVEEAVEQALWLKTKLGVSLLQALVILAAAANTGTTVEEAVQEALQLKTKLGVSLETALAILSAAAQLGTT<br>VEEAVNRALNLKTKLGVDLITAALALLTAAKLGTTVEEAVQEALQLKTKLGVSLIEAL**H**ILLTAAVLGTTVEEAVYRALKLKTKLGVSLL<br>QAAAILILAARLGTTVEQAVERALQLKTKLGGGSGG*SHHWGSGSLEHHHHHH*
DNA sequence:	ATGGTGAGCCTGGATCAGGCGATTATTATTCTGATTGTGGCGGCGAAACTGGGCACCACCGTGGAAGAAGCGGTGGAACAGGCGCTGTGG<br>CTGAAAACCAAATTAGGCGTGAGTCTGCTGCAGGCACTGGTGATTCTGGCCGCCGCTGCCAATACCGGTACCACGGTTGAAGAGGCCGTT<br>CAAGAGGCGTTGCAATTGAAAACGAAGTTGGGTGTTAGCTTGGAAACCGCGTTGGCGATTTTAAGCGCGGCGGCGCAGCTGGGTACGACC<br>GTCGAGGAGGCGGTGAATCGCGCGCTGAATCTGAAGACGAAGCTGGGCGTGGATCTGATTACCGCGGCTTTAGCCCTGCTGACCGCAGCT<br>AAACTAGGCACTACAGTCGAGGAAGCTGTACAGGAGGCTTTGCAGCTTAAAACTAAACTAGGCGTTTCACTGATAGAAGCATTGCATATC<br>CTGTTGACGGCCGCAGTACTGGGGACAACTGTAGAAGAGGCTGTATATCGAGCGCTTAAACTCAAGACAAAGCTAGGGGTTAGTTTGCTC<br>CAGGCAGCGGCCATATTGATTTTAGCAGCGCGCCTGGGGACTACAGTAGAGCAGGCTGTAGAACGCGCATTGCAACTAAAGACTAAGCTT<br>GGTGGCGGGAGCGGTGGCTCCCATCATTGGGGCAGCGGCAGCCTCGAGCACCACCACCACCACCAC
**dnHEM1-RR7**	
AA sequence:	MVSLDEAIIILIVAAKLGTTVEEAVEQALWLKTKLGVSLVQALFILLAAANTGTTVEEAVQEALQLKTKLGVSLEAALAILSAAAQLGTT<br>VEEAVNRALNLKTKLGVDLLTAALALLTAAKLGTTVEEAVQEALQLKTKLGVSLIEAL**H**ILLTAAVLGTTVEEAVYRALKLKTKLGVSLL<br>QAAAILILAARLGTTVEQAVERALQLKTKLGGGSGG*SHHWGSGSLEHHHHHH*
DNA sequence:	ATGGTGAGCCTGGATGAAGCGATTATTATTCTGATTGTGGCGGCGAAACTGGGCACCACCGTGGAAGAAGCGGTGGAACAGGCGCTGTGG<br>CTGAAAACCAAATTAGGCGTGTCGCTGGTGCAGGCCCTGTTTATCCTGCTGGCCGCCGCCAATACCGGCACGACGGTTGAGGAGGCGGTT<br>CAGGAAGCGCTGCAGTTGAAGACGAAGTTGGGCGTATCGTTGGAAGCGGCGTTGGCGATTTTAAGCGCGGCAGCGCAACTGGGTACCACG<br>GTGGAGGAAGCCGTTAATCGCGCGTTGAATTTGAAAACGAAATTGGGGGTGGATTTGTTAACCGCGGCTTTAGCCCTGCTGACCGCAGCT<br>AAACTAGGCACTACAGTCGAGGAAGCTGTACAGGAGGCTTTGCAGCTTAAAACTAAACTAGGCGTTTCACTGATAGAAGCATTGCATATC<br>CTGTTGACGGCCGCAGTACTGGGGACAACTGTAGAAGAGGCTGTATATCGAGCGCTTAAACTCAAGACAAAGCTAGGGGTTAGTTTGCTC<br>CAGGCAGCGGCCATATTGATTTTAGCAGCGCGCCTGGGGACTACAGTAGAGCAGGCTGTAGAACGCGCATTGCAACTAAAGACTAAGCTT<br>GGTGGCGGGAGCGGTGGCTCCCATCATTGGGGCAGCGGCAGCCTCGAGCACCACCACCACCACCAC
**dnHEM1-RR8**	
AA sequence:	MVSLDQAIIILIVAAKLGTTVEEAVEQALWLKTKLGVSLFQALLILAAAANTGTTVEEAVQEALQLKTKLGVSLETALAILSAAAQLGTT<br>VEEAVNRALNLKTKLGVDLTTAALALLTAAKLGTTVEEAVQEALQLKTKLGVSLIEAL**H**ILLTAAVLGTTVEEAVYRALKLKTKLGVSLL<br>QAAAILILAARLGTTVEQAVERALQLKTKLGGGSGG*SHHWGSGSLEHHHHHH*
DNA sequence:	ATGGTGAGCCTGGATCAGGCGATTATTATTCTGATTGTGGCGGCGAAACTGGGCACCACCGTGGAAGAAGCGGTGGAACAGGCGCTGTGG<br>CTGAAAACCAAATTAGGCGTGAGTCTGTTTCAGGCACTGCTGATTCTGGCCGCCGCTGCCAATACCGGCACGACGGTTGAGGAGGCAGTG<br>CAGGAAGCGCTGCAGTTGAAAACGAAGTTGGGTGTGAGCTTGGAAACCGCGTTGGCGATTTTAAGCGCGGCAGCGCAGCTGGGTACGACT<br>GTGGAGGAGGCAGTGAATCGCGCGCTGAATCTGAAGACGAAGCTCGGCGTGGATTTAACCACCGCGGCTTTAGCCCTGCTGACCGCAGCT<br>AAACTAGGCACTACAGTCGAGGAAGCTGTACAGGAGGCTTTGCAGCTTAAAACTAAACTAGGCGTTTCACTGATAGAAGCATTGCATATC<br>CTGTTGACGGCCGCAGTACTGGGGACAACTGTAGAAGAGGCTGTATATCGAGCGCTTAAACTCAAGACAAAGCTAGGGGTTAGTTTGCTC<br>CAGGCAGCGGCCATATTGATTTTAGCAGCGCGCCTGGGGACTACAGTAGAGCAGGCTGTAGAACGCGCATTGCAACTAAAGACTAAGCTT<br>GGTGGCGGGAGCGGTGGCTCCCATCATTGGGGCAGCGGCAGCCTCGAGCACCACCACCACCACCAC
**dnHEM1-RR9**	
AA sequence:	MVSLDLAIVILIVAAKLGTTVEEAVQQALWLKTKLGVSLLQALFILLAAANTGTTVEEAVQEALQLKTKLGVSLEAALAILSAAAQLGTT<br>VEEAVNRALNLKTKLGVDLLTAALALLTAAKLGTTVEEAVQEALQLKTKLGVSLIEAL**H**ILLTAAVLGTTVEEAVYRALKLKTKLGVSLL<br>QAAAILILAARLGTTVEQAVERALQLKTKLGGGSGG*SHHWGSGSLEHHHHHH*
DNA sequence:	ATGGTGAGCCTGGATCTGGCGATTGTGATTCTGATTGTGGCGGCGAAACTGGGCACCACCGTGGAAGAAGCGGTGCAGCAGGCGCTGTGG<br>CTGAAAACCAAATTAGGCGTGAGTCTGCTGCAGGCCCTGTTTATTCTGCTGGCCGCCGCCAATACCGGCACGACGGTTGAGGAGGCGGTG<br>CAGGAAGCGCTGCAGTTGAAGACCAAGTTGGGTGTTTCTTTGGAAGCGGCGTTGGCCATTTTAAGCGCGGCAGCGCAACTGGGTACCACG<br>GTCGAGGAAGCCGTTAATCGCGCGTTGAATTTGAAAACGAAGCTGGGGGTGGATTTGTTAACCGCGGCTTTAGCCCTGCTGACCGCAGCT<br>AAACTAGGCACTACAGTCGAGGAAGCTGTACAGGAGGCTTTGCAGCTTAAAACTAAACTAGGCGTTTCACTGATAGAAGCATTGCATATC

	CTGTTGACGGCCGCAGTACTGGGGACAACTGTAGAAGAGGCTGTATATCGAGCGCTTAAACTCAAGACAAAGCTAGGGGTTAGTTTGCTC<br>CAGGCAGCGGCCATATTGATTTTAGCAGCGCGCCTGGGGACTACAGTAGAGCAGGCTGTAGAACGCGCATTGCAACTAAAGACTAAGCTT<br>GGTGGCGGGAGCGGTGGCTCCCATCATTGGGGCAGCGGCAGCCTCGAGCACCACCACCACCACCAC
**dnHEM1-RR10**	
AA sequence:	MVSLDEAIIILIVAAKLGTTVEEAVEQALWLKTKLGVSLLQALFILLAAANTGTTVEEAVQEALQLKTKLGVSLEAALAILSAAAQLGTT<br>VEEAVNRALNLKTKLGVDLLTAALALLTAAKLGTTVEEAVQEALQLKTKLGVSLIEAL**H**ILLTAAVLGTTVEEAVYRALKLKTKLGVSLL<br>QAAAILILAARLGTTVEEAVERALQLKTKLGGGSGG*SHHWGSGSLEHHHHHH*
DNA sequence:	ATGGTGAGCCTGGATGAAGCGATTATTATTTTAATTGTTGCGGCGAAATTAGGTACCACCGTTGAAGAAGCGGTTGAACAAGCGTTATGG<br>TTAAAAACCAAACTGGGTGTTAGTTTATTACAAGCATTATTTATCTTATTAGCAGCCGCCAATACCGGTACTACTGTTGAGGAGGCAGTT<br>CAAGAGGCGTTGCAATTAAAGACGAAGTTGGGTGTTTCGTTGGAAGCGGCGTTGGCGATTTTAAGCGCGGCGGCGCAACTGGGTACGACT<br>GTGGAGGAAGCCGTTAATCGTGCGTTGAATTTGAAAACTAAATTGGGGGTGGATTTGTTAACCGCGGCGCTGGCGCTGCTGACCGCCGCA<br>AAGCTCGGGACGACGGTAGAGGAGGCCGTTCAGGAGGCCTTACAGCTCAAGACCAAACTCGGCGTTAGCTTGATTGAAGCTTTACATATC<br>CTTTTAACTGCAGCGGTTTTAGGCACCACGGTGGAAGAGGCTGTTTATCGTGCCTTAAAACTGAAGACCAAGCTGGGCGTCTCGTTGTTA<br>CAGGCGGCAGCAATTCTGATTTTAGCGGCGCGTCTGGGGACGACGGTGGAAGAAGCGGTTGAACGCGCATTGCAGCTGAAAACCAAGCTG<br>GGTGGTGGCTCGGGTGGGTCGCATCATTGGGGTAGCGGTAGCCTCGAGCACCACCACCACCACCAC
**dnHEM1-RR11**	
AA sequence:	MVSLDEAIIILIVAAKLGTTVEEAVEQALWLKTKLGVSLIQALFILLAAANTGTTVEEAVQEALQLKTKLGVSLEAALAILSAAAQLGTT<br>VEEAVNRALNLKTKLGVDLLTAALALLTAAKLGTTVEEAVQEALQLKTKLGVSLIEAL**H**ILLTAAVLGTTVEEAVYRALKLKTKLGVSLL<br>QAAAILILAARLGTTVEQAVERALQLKTKLGGGSGG*SHHWGSGSLEHHHHHH*
DNA sequence:	ATGGTGAGCCTGGATGAAGCGATTATTATTCTGATTGTGGCGGCGAAACTGGGCACCACCGTGGAAGAAGCGGTGGAACAGGCGCTGTGG<br>CTGAAAACCAAATTAGGCGTGAGTCTGATTCAGGCCCTGTTTATCCTGCTGGCCGCCGCCAATACCGGCACGACGGTTGAGGAGGCAGTG<br>CAGGAAGCGCTGCAGTTGAAGACGAAGTTGGGTGTTTCGTTGGAAGCGGCGTTGGCGATTTTAAGCGCGGCGGCGCAACTGGGTACCACG<br>GTGGAGGAAGCCGTTAATCGCGCGTTGAATTTGAAAACGAAACTCGGCGTGGATTTGTTAACCGCGGCTTTAGCCCTGCTGACCGCAGCT<br>AAACTAGGCACTACAGTCGAGGAAGCTGTACAGGAGGCTTTGCAGCTTAAAACTAAACTAGGCGTTTCACTGATAGAAGCATTGCATATC<br>CTGTTGACGGCCGCAGTACTGGGGACAACTGTAGAAGAGGCTGTATATCGAGCGCTTAAACTCAAGACAAAGCTAGGGGTTAGTTTGCTC<br>CAGGCAGCGGCCATATTGATTTTAGCAGCGCGCCTGGGGACTACAGTAGAGCAGGCTGTAGAACGCGCATTGCAACTAAAGACTAAGCTT<br>GGTGGCGGGAGCGGTGGCTCCCATCATTGGGGCAGCGGCAGCCTCGAGCACCACCACCACCACCAC
**dnHEM1-RR12**	
AA sequence:	MVSLDFAIVILIVAAKLGTTVEEAVEQALWLKTKLGVSLFQAAFILLAAANTGTTVEEAVQEALQLKTKLGVSLEAALAILSAAAQLGTT<br>VEEAVNRALNLKTKLGVDLLTAALALLTAAKLGTTVEEAVQEALQLKTKLGVSLIEAL**H**ILLTAAVLGTTVEEAVYRALKLKTKLGVSLL<br>QAAAILILAARLGTTVEQAVERALQLKTKLGGGSGG*SHHWGSGSLEHHHHHH*
DNA sequence:	ATGGTGAGCCTGGATTTTGCGATTGTGATTCTGATTGTGGCGGCGAAACTGGGCACCACCGTGGAAGAAGCGGTGGAACAGGCGCTGTGG<br>CTGAAAACCAAATTAGGCGTGAGTCTGTTTCAGGCGGCGTTTATTCTGCTGGCCGCCGCCAATACCGGCACGACCGTAGAGGAGGCAGTG<br>CAGGAAGCGCTGCAGTTGAAGACCAAGCTCGGTGTTTCGTTGGAAGCGGCCTTGGCGATTTTAAGCGCGGCGGCGCAACTGGGTACCACG<br>GTTGAGGAAGCCGTTAATCGCGCGTTGAATTTGAAAACGAAGTTGGGCGTGGATTTGTTAACCGCGGCTTTAGCCCTGCTGACCGCAGCT<br>AAACTAGGCACTACAGTCGAGGAAGCTGTACAGGAGGCTTTGCAGCTTAAAACTAAACTAGGCGTTTCACTGATAGAAGCATTGCATATC<br>CTGTTGACGGCCGCAGTACTGGGGACAACTGTAGAAGAGGCTGTATATCGAGCGCTTAAACTCAAGACAAAGCTAGGGGTTAGTTTGCTC<br>CAGGCAGCGGCCATATTGATTTTAGCAGCGCGCCTGGGGACTACAGTAGAGCAGGCTGTAGAACGCGCATTGCAACTAAAGACTAAGCTT<br>GGTGGCGGGAGCGGTGGCTCCCATCATTGGGGCAGCGGCAGCCTCGAGCACCACCACCACCACCAC
**dnHEM1-SS13**	
AA sequence:	MVSLDLAILILILAAKLGTTVEEAVEQALWLKTKLGVSLEQAAIILFAAANTGTTVEEAVQEALQLKTKLGVSLEAALAILSAAAQLGTT<br>VEEAVNRALNLKTKLGVDLLTAALALLTAAKLGTTVEEAVQEALQLKTKLGVSLIEAL**H**ILLTAAVLGTTVEEAVYRALKLKTKLGVSLL<br>QAAAILILAARLGTTVEQAVERALQLKTKLGGGSGG*SHHWGSGSLEHHHHHH*
DNA sequence:	ATGGTGAGCCTGGATCTGGCGATTCTGATCTTGATTCTGGCGGCGAAACTGGGCACCACCGTGGAAGAAGCGGTGGAACAGGCGCTGTGG<br>CTGAAAACCAAATTAGGCGTGTCGTTGGAACAAGCCGCGATTATTCTGTTTGCGGCCGCCAATACCGGCACGACGGTAGAGGAGGCAGTG<br>CAGGAAGCGCTGCAGTTGAAGACGAAGTTGGGTGTTTCGCTGGAAGCGGCGCTGGCAATTTTAAGCGCGGCAGCGCAACTGGGTACCACT<br>GTTGAGGAAGCCGTTAATCGCGCGTTGAATTTGAAAACGAAGCTCGGGGTGGATTTGTTAACCGCGGCTTTAGCCCTGCTGACCGCAGCT<br>AAACTAGGCACTACAGTCGAGGAAGCTGTACAGGAGGCTTTGCAGCTTAAAACTAAACTAGGCGTTTCACTGATAGAAGCATTGCATATC<br>CTGTTGACGGCCGCAGTACTGGGGACAACTGTAGAAGAGGCTGTATATCGAGCGCTTAAACTCAAGACAAAGCTAGGGGTTAGTTTGCTC<br>CAGGCAGCGGCCATATTGATTTTAGCAGCGCGCCTGGGGACTACAGTAGAGCAGGCTGTAGAACGCGCATTGCAACTAAAGACTAAGCTT<br>GGTGGCGGGAGCGGTGGCTCCCATCATTGGGGCAGCGGCAGCCTCGAGCACCACCACCACCACCAC
**dnHEM1-SS14**	
AA sequence:	MVSLDQAILILIVAAKLGTTVEEAVEQALWLKTKLGVSLEQALTILAAAANTGTTVEEAVQEALQLKTKLGVSLEAALAILSAAAQLGTT<br>VEEAVNRALNLKTKLGVDLLTAALALLTAAKLGTTVEEAVQEALQLKTKLGVSLIEAL**H**ILLTAAVLGTTVEEAVYRALKLKTKLGVSLL<br>QAAAILILAARLGTTVEEAVERALQLKTKLGGGSGG*SHHWGSGSLEHHHHHH*
DNA sequence:	ATGGTGAGCCTGGATCAGGCGATTCTGATCCTGATTGTGGCGGCGAAACTGGGCACCACCGTGGAAGAAGCGGTGGAACAGGCGCTGTGG<br>CTGAAAACCAAATTAGGCGTGTCGTTGGAACAAGCACTGACCATTCTGGCCGCCGCTGCCAATACCGGCACGACGGTTGAAGAGGCCGTT<br>CAAGAAGCCCTGCAATTGAAAACGAAGTTAGGTGTGAGCTTGGAAGCGGCGTTGGCGATTTTAAGCGCGGCAGCGCAACTGGGTACCACG<br>GTGGAGGAGGCGGTTAATCGCGCGTTGAATTTGAAGACGAAGCTCGGCGTGGATTTGTTAACCGCGGCTTTAGCCCTGCTGACCGCCGCC<br>AAGCTCGGTACGACCGTAGAAGAGGCTGTTCAAGAAGCTCTGCAATTAAAAACTAAATTAGGCGTAAGCTTGATTGAAGCCTTGCATATT<br>CTGTTGACGGCCGCGGTGCTGGGGACCACTGTCGAAGAAGCAGTGTATCGTGCGCTGAAACTGAAGACCAAGCTGGGGGTCAGTCTGCTG<br>CAGGCAGCGGCGATCTTGATTTTAGCGGCGCGCCTGGGCACCACTGTGGAGGAGGCTGTTGAACGCGCATTGCAATTGAAGACTAAACTC<br>GGTGGCGGGAGCGGTGGCTCCCATCATTGGGGCAGCGGCAGCCTCGAGCACCACCACCACCACCAC
**dnHEM1-SS15**	
AA sequence:	MVSLDQAIIILIVAAKLGTTVEEAVEQALWLKTKLGVSLLQALTILAAAANTGTTVEEAVQEALQLKTKLGVSLEAALAILSAAAQLGTT<br>VEEAVNRALNLKTKLGVDLLTAALALLTAAKLGTTVEEAVQEALQLKTKLGVSLIEAL**H**ILLTAAVLGTTVEEAVYRALKLKTKLGVSLL<br>QAAAILILAARLGTTVEQAVERALQLKTKLGGGSGG*SHHWGSGSLEHHHHHH*
DNA sequence:	ATGGTGAGCCTGGATCAGGCGATTATTATTCTGATTGTGGCGGCGAAACTGGGCACCACCGTGGAAGAAGCGGTGGAACAGGCGCTGTGG<br>CTGAAAACCAAATTAGGCGTGAGTCTGCTGCAGGCACTGACCATTCTGGCCGCCGCTGCCAATACCGGTACCACGGTTGAAGAGGCCGTT

	CAAGAGGCGTTGCAATTGAAAACGAAGTTGGGTGTTAGCTTGGAAGCGGCGTTGGCGATTTTAAGCGCGGCAGCGCAATTGGGTACGACG<br>GTCGAGGAGGCGGTTAATCGCGCGTTGAATTTGAAGACGAAGCTGGGGGTGGATTTGTTAACCGCGGCTTTAGCCCTGCTGACCGCAGCT<br>AAACTAGGCACTACAGTCGAGGAAGCTGTACAGGAGGCTTTGCAGCTTAAAACTAAACTAGGCGTTTCACTGATAGAAGCATTGCATATC<br>CTGTTGACGGCCGCAGTACTGGGGACAACTGTAGAAGAGGCTGTATATCGAGCGCTTAAACTCAAGACAAAGCTAGGGGTTAGTTTGCTC<br>CAGGCAGCGGCCATATTGATTTTAGCAGCGCGCCTGGGGACTACAGTAGAGCAGGCTGTAGAACGCGCATTGCAACTAAAGACTAAGCTT<br>GGTGGCGGGAGCGGTGGCTCCCATCATTGGGGCAGCGGCAGCCTCGAGCACCACCACCACCACCAC
**dnHEM1-SS16**	
AA sequence:	MVSLDQAILILIVAAKLGTTVEEAVEQALWLKTKLGVSLEQALFILVAAANTGTTVEEAVQEALQLKTKLGVSLEAALAILSAAAQLGTT<br>VEEAVNRALNLKTKLGVDLLTAALALLTAAKLGTTVEEAVQEALQLKTKLGVSLIEAL**H**ILLTAAVLGTTVEEAVYRALKLKTKLGVSLL<br>QAAAILILAARLGTTVEEAVERALQLKTKLGGGSGG*SHHWGSGSLEHHHHHH*
DNA sequence:	ATGGTGAGCCTGGATCAGGCGATTCTGATCCTGATTGTGGCGGCGAAACTGGGCACCACCGTGGAAGAAGCGGTGGAACAGGCGCTGTGG<br>CTGAAAACCAAATTAGGTGTGAGTTTGGAACAAGCTTTGTTTATTTTAGTTGCAGCAGCAAATACCGGTACTACTGTTGAAGAGGCCGTT<br>CAAGAGGCGTTGCAATTAAAAACGAAGTTGGGTGTTTCTTTAGAGGCGGCGTTGGCGATTTTAAGCGCGGCGGCGCAACTCGGTACCACT<br>GTGGAGGAAGCCGTTAATCGCGCGTTGAATTTGAAAACTAAACTCGGGGTGGATTTGTTAACCGCGGCGCTGGCGCTGCTGACCGCAGCC<br>AAGCTCGGCACGACTGTGGAGGAAGCTGTTCAGGAGGCCTTACAGTTAAAGACTAAGCTGGGCGTTAGCTTGATTGAAGCATTGCATATT<br>TTATTAACGGCTGCGGTGCTGGGGACGACCGTTGAGGAGGCAGTTTATCGTGCGCTGAAACTGAAGACCAAGTTGGGTGTTAGTTTGTTA<br>CAAGCAGCTGCAATTTTAATTTTAGCGGCGCGTCTGGGTACGACGGTTGAGGAGGCTGTGGAACGCGCACTGCAGTTGAAAACGAAATTA<br>GGTGGGGGGAGCGGCGGTTCCCATCATTGGGGCAGCGGCAGCCTCGAGCACCACCACCACCACCAC
**dnHEM1-SS16_pI10**	
AA sequence:	MSGVSLDQAILILIVAAKLGTTVEEAVKRALWLKTKLGVSLEQALFILVAAANTGTTVEEAVKRALKLKTKLGVSLEAALAILSAAAQLG<br>TTVEEAVKRALKLKTKLGVDLLTAALALLTAAKLGTTVEEAVKRALKLKTKLGVSLIEAL**H**ILLTAAVLGTTVEEAVYRALKLKTKLGVS<br>LLQAAAILILAARLGTTVEEAVKRALKLKTKLGGGSG*SHHWGSTHHHHHH*
DNA sequence:	ATGTCAGGAGTGAGCCTGGATCAGGCGATTCTGATTCTGATTGTGGCGGCGAAACTGGGCACCACCGTGGAAGAAGCGGTGAAACGCGCG<br>CTGTGGCTGAAAACCAAATTAGGCGTGTCGTTGGAACAGGCGCTGTTTATTCTGGTGGCGGCCGCCAATACCGGCACGACGGTTGAAGAG<br>GCCGTTAAACGTGCACTGAAACTGAAGACGAAGTTGGGTGTTTCGCTGGAAGCGGCGCTGGCGATTTTAAGCGCCGCGGCGCAGCTGGGT<br>ACGACCGTTGAGGAGGCGGTTAAGCGCGCGTTGAAATTGAAAACGAAATTGGGGGTGGATCTGCTGACCGCGGCCCTGGCGTTGTTGACC<br>GCAGCCAAGCTCGGTACCACTGTGGAGGAAGCAGTCAAGCGTGCCCTGAAGTTAAAGACCAAGCTGGGGGTCAGCTTGATTGAGGCACTG<br>CATATTCTGCTGACCGCTGCGGTGTTGGGCACTACCGTAGAGGAAGCAGTGTATCGCGCCTTGAAGCTCAAGACTAAGTTAGGTGTTAGT<br>CTGCTGCAGGCGGCAGCCATCTTGATTTTAGCCGCGCGCCTGGGGACGACTGTCGAAGAGGCTGTGAAGCGCGCGCTCAAGTTGAAGACC<br>AAACTCGGTGGCGGTTCCGGCAGCCATCATTGGGGCAGCACCCACCACCACCACCACCAC
**dnHEM1-SS17**	
AA sequence:	MVSLDFAILILILAAKLGTTVEEAVEQALWLKTKLGVSLEQAAIILFAAANTGTTVEEAVQEALQLKTKLGVSLEAALAILSAAAQLGTT<br>VEEAVNRALNLKTKLGVDLLTAALALLTAAKLGTTVEEAVQEALQLKTKLGVSLIEAL**H**ILLTAAVLGTTVEEAVYRALKLKTKLGVSLL<br>QAAAILILAARLGTTVEQAVERALQLKTKLGGGSGG*SHHWGSGSLEHHHHHH*
DNA sequence:	ATGGTGAGCCTGGATTTTGCGATTCTGATCTTGATTCTGGCGGCGAAACTGGGCACCACCGTGGAAGAAGCGGTGGAACAGGCGCTGTGG<br>CTGAAAACCAAATTAGGCGTGTCGTTGGAACAAGCCGCGATTATTCTGTTTGCGGCCGCCAATACCGGCACGACGGTAGAGGAGGCAGTG<br>CAGGAAGCGCTGCAGTTGAAGACGAAGTTGGGCGTTTCGTTAGAGGCGGCGCTGGCGATTTTAAGCGCGGCAGCGCAACTGGGTACCACT<br>GTTGAGGAAGCCGTTAATCGCGCGTTGAATTTGAAAACGAAATTGGGGGTGGATTTGTTAACCGCGGCTTTAGCCCTGCTGACCGCAGCT<br>AAACTAGGCACTACAGTCGAGGAAGCTGTACAGGAGGCTTTGCAGCTTAAAACTAAACTAGGCGTTTCACTGATAGAAGCATTGCATATC<br>CTGTTGACGGCCGCAGTACTGGGGACAACTGTAGAAGAGGCTGTATATCGAGCGCTTAAACTCAAGACAAAGCTAGGGGTTAGTTTGCTC<br>CAGGCAGCGGCCATATTGATTTTAGCAGCGCGCCTGGGGACTACAGTAGAGCAGGCTGTAGAACGCGCATTGCAACTAAAGACTAAGCTT<br>GGTGGCGGGAGCGGTGGCTCCCATCATTGGGGCAGCGGCAGCCTCGAGCACCACCACCACCACCAC
**dnHEM1-SS17_pI10**	
AA sequence:	MSGVSLDFAILILILAAKLGTTVEEAVKRALWLKTKLGVSLEQAAIILFAAANTGTTVEEAVKRALKLKTKLGVSLEAALAILSAAAQLG<br>TTVEEAVKRALKLKTKLGVDLLTAALALLTAAKLGTTVEEAVKRALKLKTKLGVSLIEAL**H**ILLTAAVLGTTVEEAVYRALKLKTKLGVS<br>LLQAAAILILAARLGTTVEEAVKRALKLKTKLGGGSG*SHHWGSTHHHHHH*
DNA sequence:	ATGTCAGGAGTGAGCCTGGATTTTGCGATTCTGATTCTGATTCTGGCGGCGAAACTGGGCACCACCGTGGAAGAAGCGGTGAAACGCGCG<br>CTGTGGCTGAAAACCAAATTAGGCGTGTCGTTGGAACAGGCGGCGATTATTCTGTTTGCGGCCGCCAATACCGGCACGACGGTTGAAGAG<br>GCCGTTAAACGTGCACTGAAACTGAAGACGAAGTTGGGTGTTTCGCTGGAAGCGGCGCTGGCGATTTTAAGCGCCGCGGCGCAGCTGGGT<br>ACGACCGTTGAGGAGGCGGTTAAGCGCGCGTTGAAATTGAAAACGAAATTGGGGGTGGATCTGCTGACCGCGGCCCTGGCGTTGTTGACC<br>GCAGCCAAGCTCGGTACCACTGTGGAGGAAGCAGTCAAGCGTGCCCTGAAGTTAAAGACCAAGCTGGGGGTCAGCTTGATTGAGGCACTG<br>CATATTCTGCTGACCGCTGCGGTGTTGGGCACTACCGTAGAGGAAGCAGTGTATCGCGCCTTGAAGCTCAAGACTAAGTTAGGTGTTAGT<br>CTGCTGCAGGCGGCAGCCATCTTGATTTTAGCCGCGCGCCTGGGGACGACTGTCGAAGAGGCTGTGAAGCGCGCGCTCAAGTTGAAGACC<br>AAACTCGGTGGCGGTTCCGGCAGCCATCATTGGGGCAGCACCCACCACCACCACCACCAC
**dnHEM1-SS18**	
AA sequence:	MVSLDEAIIILIVAAKLGTTVEEAVEQALWLKTKLGVSLLQALFILAAAANTGTTVEEAVQEALQLKTKLGVSLEAALAILSAAAQLGTT<br>VEEAVNRALNLKTKLGVDLLTAALALLTAAKLGTTVEEAVQEALQLKTKLGVSLIEAL**H**ILLTAAVLGTTVEEAVYRALKLKTKLGVSLL<br>QAAAILILAARLGTTVEQAVERALQLKTKLGGGSGG*SHHWGSGSLEHHHHHH*
DNA sequence:	ATGGTGAGCCTGGATGAAGCGATTATTATTCTGATTGTGGCGGCGAAACTGGGCACCACCGTGGAAGAAGCGGTGGAACAGGCGCTGTGG<br>CTGAAAACCAAATTAGGCGTGAGTCTGCTGCAGGCCCTGTTTATCCTGGCCGCCGCTGCCAATACCGGCACGACGGTTGAGGAGGCAGTG<br>CAGGAAGCGCTGCAGTTGAAAACGAAGTTGGGTGTTTCGTTGGAAGCGGCGTTGGCGATTTTAAGCGCGGCAGCGCAACTGGGTACCACG<br>GTCGAAGAGGCCGTTAATCGCGCGTTGAATTTGAAGACGAAGCTCGGCGTGGATTTGTTAACCGCGGCTTTAGCCCTGCTGACCGCAGCT<br>AAACTAGGCACTACAGTCGAGGAAGCTGTACAGGAGGCTTTGCAGCTTAAAACTAAACTAGGCGTTTCACTGATAGAAGCATTGCATATC<br>CTGTTGACGGCCGCAGTACTGGGGACAACTGTAGAAGAGGCTGTATATCGAGCGCTTAAACTCAAGACAAAGCTAGGGGTTAGTTTGCTC<br>CAGGCAGCGGCCATATTGATTTTAGCAGCGCGCCTGGGGACTACAGTAGAGCAGGCTGTAGAACGCGCATTGCAACTAAAGACTAAGCTT<br>GGTGGCGGGAGCGGTGGCTCCCATCATTGGGGCAGCGGCAGCCTCGAGCACCACCACCACCACCAC

**dnHEM1-SS19**	
AA sequence:	MVSLDEAILILILAAKLGTTVEEAVEQALWLKTKLGVSLLQALIIILFAAANTGTTVEEAVQEALQLKTKLGVSLEAALAILSAAAQLGTT<br>VEEAVNRALNLKTKLGVDLLTAALALLTAAKLGTTVEEAVQEALQLKTKLGVSLIEAL**H**ILLTAAVLGTTVEEAVYRALKLKTKLGVSLL<br>QAAAILILAARLGTTVEQAVERALQLKTKLGGGSGG*SHHWGSGSLEHHHHHH*
DNA sequence:	ATGGTGAGCCTGGATGAAGCGATTCTGATCTTGATTCTGGCGGCGAAACTGGGCACCACCGTGGAAGAAGCGGTGGAACAGGCGCTGTGG<br>CTGAAAACCAAATTAGGCGTGAGTCTGCTGCAGGCACTGATTATTCTGTTTGCGGCCGCCAATACCGGCACGACGGTAGAGGAGGCAGTG<br>CAGGAAGCGCTGCAGTTGAAGACGAAGTTGGGCGTTTCGTTGGAAGCGGCGCTGGCGATTTTAAGCGCGGCGGCGCAACTGGGTACCACG<br>GTTGAGGAAGCCGTTAATCGCGCGTTGAATTTGAAAACGAAACTCGGGGTGGATTTGTTAACCGCGGCTTTAGCCCTGCTGACCGCAGCT<br>AAACTAGGCACTACAGTCGAGGAAGCTGTACAGGAGGCTTTGCAGCTTAAAACTAAACTAGGCGTTTCACTGATAGAAGCATTGCATATC<br>CTGTTGACGGCCGCAGTACTGGGGACAACTGTAGAAGAGGCTGTATATCGAGCGCTTAAACTCAAGACAAAGCTAGGGGTTAGTTTGCTC<br>CAGGCAGCGGCCATATTGATTTTAGCAGCGCGCCTGGGGACTACAGTAGAGCAGGCTGTAGAACGCGCATTGCAACTAAAGACTAAGCTT<br>GGTGGCGGGAGCGGTGGCTCCCATCATTGGGGCAGCGGCAGCCTCGAGCACCACCACCACCACCAC
**dnHEM1-SS19_pI10**	
AA sequence:	MSGVSLDEAILILILAAKLGTTVEEAVKRALWLKTKLGVSLLQALIIILFAAANTGTTVEEAVKRALKLKTKLGVSLEAALAILSAAAQLG<br>TTVEEAVKRALKLKTKLGVDLLTAALALLTAAKLGTTVEEAVKRALKLKTKLGVSLIEAL**H**ILLTAAVLGTTVEEAVYRALKLKTKLGVS<br>LLQAAAILILAARLGTTVEEAVKRALKLKTKLGGGSG*SHHWGSTHHHHHH*
DNA sequence:	ATGTCAGGAGTGAGCCTGGATGAAGCGATTCTGATTCTGATTCTGGCGGCGAAACTGGGCACCACCGTGGAAGAAGCGGTGAAACGCGCG<br>CTGTGGCTGAAAACCAAATTAGGCGTGTCGTTGCTGCAGGCGCTGATTATTCTGTTTGCGGCCGCCAATACCGGCACGACGGTTGAAGAG<br>GCCGTTAAACGTGCACTGAAACTGAAGACGAAGTTGGGTGTTTCGCTGGAAGCGGCGCTGGCGATTTTAAGCGCCGCGGCGCAGCTGGGT<br>ACGACCGTTGAGGAGGCGGTTAAGCGCGCGTTGAAATTGAAAACGAAATTGGGGGTGGATCTGCTGACCGCGGCCCTGGCGTTGTTGACC<br>GCAGCCAAGCTCGGTACCACTGTGGAGGAAGCAGTCAAGCGTGCCCTGAAGTTAAAGACCAAGCTGGGGGTCAGCTTGATTGAGGCACTG<br>CATATTCTGCTGACCGCTGCGGTGTTGGGCACTACCGTAGAGGAAGCAGTGTATCGCGCCTTGAAGCTCAAGACTAAGTTAGGTGTTAGT<br>CTGCTGCAGGCGGCAGCCATCTTGATTTTAGCCGCGCGCCTGGGGACGACTGTCGAAGAGGCTGTGAAGCGCGCGCTCAAGTTGAAGACC<br>AAACTCGGTGGCGGTTCCGGCAGCCATCATTGGGGCAGCACCCACCACCACCACCACCAC
**dnHEM1-SS21**	
AA sequence:	MVSLDQAILILIVAAKLGTTVEEAVEQALWLKTKLGVSLIQALLILAAAANTGTTVEEAVQEALQLKTKLGVSLEAALAILSAAAQLGTT<br>VEEAVNRALNLKTKLGVDLLTAALALLTAAKLGTTVEEAVQEALQLKTKLGVSLIEAL**H**ILLTAAVLGTTVEEAVYRALKLKTKLGVSLL<br>QAAAILILAARLGTTVEEAVERALQLKTKLGGGSGG*SHHWGSGSLEHHHHHH*
DNA sequence:	ATGGTGAGCCTGGATCAGGCGATTCTGATCTTAATTGTGGCGGCGAAATTAGGTACCACCGTTGAAGAAGCGGTTGAACAAGCGTTATGG<br>TTAAAAACCAAACTTGGTGTTAGTTTAATTCAAGCATTATTAATTTTAGCAGCAGCTGCAAATACCGGTACTACCGTCGAGGAGGCAGTT<br>CAAGAGGCGTTGCAATTAAAGACTAAACTCGGGGTCAGCTTAGAGGCGGCGTTGGCGATTTTAAGCGCGGCGGCGCAATTAGGCACGACT<br>GTTGAGGAAGCAGTTAATCGTGCGTTGAATTTGAAGACGAAGCTCGGCGTGGATTTGTTAACCGCGGCGCTGGCCTTGCTGACCGCCGCA<br>AAGCTGGGTACGACCGTAGAAGAGGCTGTTCAGGAGGCATTGCAGCTCAAGACCAAGTTAGGCGTCTCGTTGATTGAAGCTTTGCATATT<br>TTATTAACTGCCGCGGTGCTGGGGACGACGGTCGAAGAGGCCGTGTATCGCGCGCTGAAATTAAAGACGAAGTTGGGGGTAAGCCTGTTG<br>CAAGCAGCGGCAATTTTAATCCTCGCCGCGCGTCTGGGCACTACGGTGGAAGAGGCCGTGGAACGCGCACTGCAATTGAAAACCAAACTT<br>GGTGGTGGCTCGGGGGGCAGCCATCATTGGGGTAGCGGTAGCCTCGAGCACCACCACCACCACCAC
**dnHEM1-SS22**	
AA sequence:	MVSLDEAIIILLVAAKLGTTVEEAVEQALWLKTKLGVSLLQALIIILLAAANTGTTVEEAVQEALQLKTKLGVSLEAALAILSAAAQLGTT<br>VEEAVNRALNLKTKLGVDLLTAALALLTAAKLGTTVEEAVQEALQLKTKLGVSLIEAL**H**ILLTAAVLGTTVEEAVYRALKLKTKLGVSLL<br>QAAAILILAARLGTTVEEAVERALQLKTKLGGGSGG*SHHWGSGSLEHHHHHH*
DNA sequence:	ATGGTGAGCCTGGATGAAGCGATTATTATTCTGCTGGTGGCGGCGAAACTGGGCACCACCGTGGAAGAAGCGGTGGAACAGGCGCTGTGG<br>CTGAAAACCAAATTAGGCGTGAGTCTGCTGCAGGCACTGATTATCTTGCTGGCCGCCGCCAATACCGGCACGACGGTTGAGGAGGCAGTG<br>CAGGAAGCGCTGCAGTTGAAGACGAAGTTGGGTGTATCGTTGGAAGCGGCGTTGGCGATTTTAAGCGCGGCGGCGCAACTGGGTACCACT<br>GTGGAGGAAGCCGTTAATCGCGCGTTGAATTTGAAAACGAAACTCGGGGTGGATTTGTTAACCGCGGCTTTAGCCCTGCTGACCGCCGCC<br>AAGCTCGGTACGACCGTAGAAGAGGCTGTTCAAGAAGCTCTGCAATTAAAAACTAAATTAGGCGTAAGCTTGATTGAAGCCTTGCATATT<br>CTGTTGACGGCCGCGGTGCTGGGGACCACTGTCGAAGAAGCAGTGTATCGTGCGCTGAAACTGAAGACCAAGCTGGGGGTCAGTCTGCTG<br>CAGGCAGCGGCGATCTTGATTTTAGCGGCGCGCCTGGGCACCACTGTGGAGGAGGCTGTTGAACGCGCATTGCAATTGAAGACTAAACTC<br>GGTGGCGGGAGCGGTGGCTCCCATCATTGGGGCAGCGGCAGCCTCGAGCACCACCACCACCACCAC
**dnHEM1-SS23**	
AA sequence:	MVSLDEALVILLVAAKLGTTVEEAVEQALWLKTKLGVSLLQALIIILLAAANTGTTVEEAVQEALQLKTKLGVSLEAALAILSAAAQLGTT<br>VEEAVNRALNLKTKLGVDLLTAALALLTAAKLGTTVEEAVQEALQLKTKLGVSLIEAL**H**ILLTAAVLGTTVEEAVYRALKLKTKLGVSLL<br>QAAAILILAARLGTTVEQAVERALQLKTKLGGGSGG*SHHWGSGSLEHHHHHH*
DNA sequence:	ATGGTGAGCCTGGATGAAGCGCTGGTGATTCTGCTGGTGGCGGCGAAACTGGGCACCACCGTGGAAGAAGCGGTGGAACAGGCGCTGTGG<br>CTGAAAACCAAATTAGGCGTGAGTCTGCTGCAGGCACTGATTATTTTGCTGGCCGCCGCCAATACCGGCACGACGGTTGAGGAGGCGGTG<br>CAGGAAGCACTGCAGTTGAAGACGAAGTTGGGTGTATCGTTGGAAGCGGCGTTGGCGATTTTAAGCGCGGCGGCGCAACTGGGTACCACG<br>GTCGAAGAGGCCGTTAATCGCGCGTTGAATTTGAAAACGAAGCTCGGCGTGGATTTGTTAACCGCGGCTTTAGCCCTGCTGACCGCAGCT<br>AAACTAGGCACTACAGTCGAGGAAGCTGTACAGGAGGCTTTGCAGCTTAAAACTAAACTAGGCGTTTCACTGATAGAAGCATTGCATATC<br>CTGTTGACGGCCGCAGTACTGGGGACAACTGTAGAAGAGGCTGTATATCGAGCGCTTAAACTCAAGACAAAGCTAGGGGTTAGTTTGCTC<br>CAGGCAGCGGCCATATTGATTTTAGCAGCGCGCCTGGGGACTACAGTAGAGCAGGCTGTAGAACGCGCATTGCAACTAAAGACTAAGCTT<br>GGTGGCGGGAGCGGTGGCTCCCATCATTGGGGCAGCGGCAGCCTCGAGCACCACCACCACCACCAC
**dnHEM1-SS24**	
AA sequence:	MVSLDEAIIILIVAAKLGTTVEEAVEQALWLKTKLGVSLLQALIIILLAAANTGTTVEEAVQEALQLKTKLGVSLEIALAILSAAAQLGTT<br>VEEAVNRALNLKTKLGVDLATAALALLTAAKLGTTVEEAVQEALQLKTKLGVSLIEAL**H**ILLTAAVLGTTVEEAVYRALKLKTKLGVSLL<br>QAAAILILAARLGTTVEQAVERALQLKTKLGGGSGG*SHHWGSGSLEHHHHHH*
DNA sequence:	ATGGTGAGCCTGGATGAAGCGATTATTATTCTGATTGTGGCGGCGAAACTGGGCACCACCGTGGAAGAAGCGGTGGAACAGGCGCTGTGG<br>CTGAAAACCAAATTAGGCGTGAGTCTGCTGCAGGCACTGATTATTCTGCTGGCCGCCGCCAATACCGGCACGACGGTAGAGGAGGCAGTG<br>CAGGAAGCGCTGCAGTTGAAGACGAAGTTGGGTGTTTCGTTGGAAATTGCGCTGGCGATTTTAAGCGCGGCAGCGCAGCTGGGTACGACT<br>GTTGAGGAAGCAGTGAATCGCGCGCTGAATCTGAAGACCAAGCTGGGCGTGGATTTAGCGACCGCGGCTTTAGCCCTGCTGACCGCAGCT<br>AAACTAGGCACTACAGTCGAGGAAGCTGTACAGGAGGCTTTGCAGCTTAAAACTAAACTAGGCGTTTCACTGATAGAAGCATTGCATATC

	CTGTTGACGGCCGCAGTACTGGGGACAACTGTAGAAGAGGCTGTATATCGAGCGCTTAAACTCAAGACAAAGCTAGGGGTTAGTTTGCTC<br>CAGGCAGCGGCCATATTGATTTTAGCAGCGCGCCTGGGGACTACAGTAGAGCAGGCTGTAGAACGCGCATTGCAACTAAAGACTAAGCTT<br>GGTGGCGGGAGCGGTGGCTCCCATCATTGGGGCAGCGGCAGCCTCGAGCACCACCACCACCACCAC
**dnHEM1-RR25**	
AA sequence:	MVSLDIAIVILLVAAKLGTTVEEAVQQALWLKTKLGVSLLQALLILAAAANTGTTVEEAVQEALQLKTKLGVSLEEALAILSAAAQLGTT<br>VEEAVNRALNLKTKLGVDLLTAALALLTAAKLGTTVEEAVQEALQLKTKLGVSLIEAL**H**ILLTAAVLGTTVEEAVYRALKLKTKLGVSLL<br>QAAAILILAARLGTTVEEAVERALQLKTKLGGGSGG*SHHWGSGSLEHHHHHH*
DNA sequence:	ATGGTGAGCCTGGATATTGCGATTGTGATTCTGCTGGTGGCGGCGAAACTGGGCACCACCGTGGAAGAAGCGGTGCAGCAGGCGCTGTGG<br>CTGAAAACCAAATTAGGCGTGAGTCTGCTGCAGGCGTTGTTGATCCTGGCCGCCGCTGCCAATACCGGTACCACGGTTGAAGAGGCCGTT<br>CAAGAAGCCTTGCAATTGAAAACGAAGTTGGGTGTTAGCTTGGAAGAAGCGCTGGCGATTTTAAGCGCGGCAGCGCAGCTGGGTACGACC<br>GTTGAGGAGGCAGTGAATCGCGCGCTGAATCTGAAGACGAAGCTCGGCGTGGATTTGTTAACCGCGGCTTTAGCCCTGCTGACCGCCGCC<br>AAGCTCGGTACGACCGTAGAAGAGGCTGTTCAAGAAGCTCTGCAATTAAAAACTAAATTAGGCGTAAGCTTGATTGAAGCCTTGCATATT<br>CTGTTGACGGCCGCGGTGCTGGGGACCACTGTCGAAGAAGCAGTGTATCGTGCGCTGAAACTGAAGACCAAGCTGGGGGTCAGTCTGCTG<br>CAGGCAGCGGCGATCTTGATTTTAGCGGCGCGCCTGGGCACCACTGTGGAGGAGGCTGTTGAACGCGCATTGCAATTGAAGACTAAACTC<br>GGTGGCGGGAGCGGTGGCTCCCATCATTGGGGCAGCGGCAGCCTCGAGCACCACCACCACCACCAC
**dnHEM1-RR25_pI10**	
AA sequence:	MSGVSLDIAIVILLVAAKLGTTVEEAVKRALWLKTKLGVSLLQALLILAAAANTGTTVEEAVKRALKLKTKLGVSLEEALAILSAAAQLG<br>TTVEEAVKRALKLKTKLGVDLLTAALALLTAAKLGTTVEEAVKRALKLKTKLGVSLIEAL**H**ILLTAAVLGTTVEEAVYRALKLKTKLGVS<br>LLQAAAILILAARLGTTVEEAVKRALKLKTKLGGGSG*SHHWGSTHHHHHH*
DNA sequence:	ATGTCAGGAGTGAGCCTGGATATTGCGATTGTGATTCTGCTGGTGGCGGCGAAACTGGGCACCACCGTGGAAGAAGCGGTGAAACGCGCG<br>CTGTGGCTGAAAACCAAATTAGGCGTGTCGTTGCTGCAGGCGCTGCTGATTCTGGCGGCGGCCGCCAATACCGGCACGACGGTTGAAGAG<br>GCCGTTAAACGTGCACTGAAACTGAAGACGAAGTTGGGTGTTTCGCTGGAAGAAGCGCTGGCGATTTTAAGCGCCGCGGCGCAGCTGGGT<br>ACGACCGTTGAGGAGGCGGTTAAGCGCGCGTTGAAATTGAAAACGAAATTGGGGGTGGATCTGCTGACCGCGGCCCTGGCGTTGTTGACC<br>GCAGCCAAGCTCGGTACCACTGTGGAGGAAGCAGTCAAGCGTGCCCTGAAGTTAAAGACCAAGCTGGGGGTCAGCTTGATTGAGGCACTG<br>CATATTCTGCTGACCGCTGCGGTGTTGGGCACTACCGTAGAGGAAGCAGTGTATCGCGCCTTGAAGCTCAAGACTAAGTTAGGTGTTAGT<br>CTGCTGCAGGCGGCAGCCATCTTGATTTTAGCCGCGCGCCTGGGGACGACTGTCGAAGAGGCTGTGAAGCGCGCGCTCAAGTTGAAGACC<br>AAACTCGGTGGCGGTTCCGGCAGCCATCATTGGGGCAGCACCCACCACCACCACCACCAC
**dnHEM1-RR26**	
AA sequence:	MVSLDIAIAILLVAAKLGTTVEEAVQQALWLKTKLGVSLIQALLILAAAANTGTTVEEAVQEALQLKTKLGVSLEEALAILSAAAQLGTT<br>VEEAVNRALNLKTKLGVDLATAALALLTAAKLGTTVEEAVQEALQLKTKLGVSLIEAL**H**ILLTAAVLGTTVEEAVYRALKLKTKLGVSLL<br>QAAAILILAARLGTTVEEAVQEALQLKTKLGGGSGG*SHHWGSGSLEHHHHHH*
DNA sequence:	ATGGTGAGCCTGGATATTGCGATTGCAATTTTATTAGTTGCGGCGAAATTAGGTACCACCGTTGAAGAAGCGGTTCAACAAGCGTTGTGG<br>TTAAAAACCAAACTTGGTGTTAGTTTAATTCAAGCATTATTAATTTTAGCAGCAGCTGCCAATACCGGTACTACTGTTGAGGAGGCAGTA<br>CAAGAGGCGTTACAATTAAAGACTAAATTGGGCGTGAGCTTAGAAGAGGCCTTAGCGATTTTAAGCGCGGCGGCGCAACTGGGGACGACT<br>GTCGAGGAAGCAGTTAATCGTGCGCTGAATTTAAAGACGAAGTTGGGTGTTGATTTAGCGACCGCGGCGTTAGCGTTATTGACGGCGGCC<br>AAGCTCGGTACGACCGTAGAGGAGGCCGTTCAGGAGGCCTTGCAGCTCAAGACCAAGCTGGGGGTTTCGTTGATTGAAGCATTGCATATT<br>TTATTAACTGCAGCGGTGTTGGGTACGACGGTCGAAGAGGCTGTTTATCGTGCCTTGAAACTGAAAACTAAACTGGGCGTTAGCCTGCTG<br>CAGGCCGCAGCCATCTTGATTTTGGCCGCGCGCCTGGGCACCACCGTTGAGGAAGCTGTTCAAGAAGCATTACAGTTGAAAACGAAGCTT<br>GGTGGTGGCAGTGGTGGGAGCCATCATTGGGGTAGCGGTAGCCTCGAGCACCACCACCACCACCAC
**dnHEM1-RR27**	
AA sequence:	MVSLDLAIIILIVAAKLGTTVEEAVQQALWLKTKLGVSLKQAFLILAAAANTGTTVEEAVQEALQLKTKLGVSLEAALAILSAAAQLGTT<br>VEEAVNRALNLKTKLGVDLLTAALALLTAAKLGTTVEEAVQEALQLKTKLGVSLIEAL**H**ILLTAAVLGTTVEEAVYRALKLKTKLGVSLL<br>QAAAILILAARLGTTVEEAVQEALQLKTKLGGGSGG*SHHWGSGSLEHHHHHH*
DNA sequence:	ATGGTGAGCCTGGATCTGGCGATTATTATTCTGATTGTGGCGGCGAAATTAGGTACCACCGTGGAAGAAGCGGTGCAACAAGCGCTGTGG<br>CTGAAAACCAAACTTGGTGTTAGTTTAAAACAGGCGTTTTTAATTTTAGCAGCAGCTGCAAATACCGGTACTACTGTTGAAGAGGCAGTT<br>CAAGAGGCCCTGCAGTTAAAGACGAAGCTCGGTGTTTCGTTGGAAGCGGCGTTAGCGATTTTAAGCGCGGCAGCGCAACTGGGTACGACG<br>GTGGAGGAGGCCGTTAATCGCGCGTTGAATTTGAAAACGAAACTCGGCGTGGATTTGTTAACCGCGGCGCTGGCGCTGCTGACCGCAGCT<br>AAGTTGGGCACTACCGTTGAGGAGGCTGTTCAGGAAGCCTTACAGCTCAAAACTAAATTGGGTGTCAGCTTGATTGAAGCATTACATATT<br>TTATTAACTGCAGCGGTGTTGGGTACGACGGTCGAGGAGGCCGTGTATCGTGCGCTGAAATTGAAGACGAAGTTAGGCGTGTCCCTGCTG<br>CAGGCAGCCGCGATCTTGATTTTGGCCGCGCGCCTGGGGACTACGGTTGAGGAGGCTGTGCAGGAGGCGTTACAATTGAAGACGAAGTTA<br>GGTGGTGGCAGTGGTGGGAGCCATCATTGGGGTAGCGGTAGCCTCGAGCACCACCACCACCACCAC
**dnHEM1-RR28**	
AA sequence:	MVSLDIAIAILVVAAKLGTTVEEAVQQALWLKTKLGVSLIQALLILAAAANTGTTVEEAVQEALQLKTKLGVSLEEALAILSAAAQLGTT<br>VEEAVNRALNLKTKLGVDLATAALALLTAAKLGTTVEEAVQEALQLKTKLGVSLIEAL**H**ILLTAAVLGTTVEEAVYRALKLKTKLGVSLL<br>QAAAILILAARLGTTVEQAVERALQLKTKLGGGSGG*SHHWGSGSLEHHHHHH*
DNA sequence:	ATGGTGAGCCTGGATATTGCGATTGCAATTCTGGTGGTGGCGGCGAAACTGGGCACCACCGTGGAAGAAGCGGTGCAGCAGGCGCTGTGG<br>CTGAAAACCAAATTAGGCGTGAGTCTGATTCAGGCACTGCTGATCCTGGCCGCCGCTGCCAATACCGGCACGACGGTTGAAGAGGCTGTG<br>CAGGAAGCGCTGCAGTTGAAAACGAAGCTCGGTGTGAGCTTGGAAGAAGCCCTGGCGATTTTAAGCGCGGCGGCGCAGCTGGGTACGACC<br>GTAGAGGAGGCAGTGAATCGCGCGCTGAATCTGAAGACGAAACTTGGCGTGGATTTAGCGACCGCGGCTTTAGCCCTGCTGACCGCAGCT<br>AAACTAGGCACTACAGTCGAGGAAGCTGTACAGGAGGCTTTGCAGCTTAAAACTAAACTAGGCGTTTCACTGATAGAAGCATTGCATATC<br>CTGTTGACGGCCGCAGTACTGGGGACAACTGTAGAAGAGGCTGTATATCGAGCGCTTAAACTCAAGACAAAGCTAGGGGTTAGTTTGCTC<br>CAGGCAGCGGCCATATTGATTTTAGCAGCGCGCCTGGGGACTACAGTAGAGCAGGCTGTAGAACGCGCATTGCAACTAAAGACTAAGCTT<br>GGTGGCGGGAGCGGTGGCTCCCATCATTGGGGCAGCGGCAGCCTCGAGCACCACCACCACCACCAC
**dnHEM1-RR29**	
AA sequence:	MVSLDQAIVILIVAAKLGTTVEEAVQQALWLKTKLGVSLLQALLILLAAANTGTTVEEAVQEALQLKTKLGVSLEAALAILSAAAQLGTT<br>VEEAVNRALNLKTKLGVDLVTAALALLTAAKLGTTVEEAVQEALQLKTKLGVSLIEAL**H**ILLTAAVLGTTVEEAVYRALKLKTKLGVSLL<br>QAAAILILAARLGTTVEQAVERALQLKTKLGGGSGG*SHHWGSGSLEHHHHHH*
DNA sequence:	ATGGTGAGCCTGGATCAGGCGATTGTGATTCTGATTGTGGCGGCGAAACTGGGCACCACCGTGGAAGAAGCGGTGCAGCAGGCGCTGTGG<br>CTGAAAACCAAATTAGGCGTGAGTCTGCTGCAAGCGCTGCTGATCCTGCTGGCCGCCGCCAATACCGGTACCACGGTTGAAGAGGCCGTT

	CAAGAAGCCCTGCAATTGAAAACGAAGCTCGGCGTTTCGTTGGAAGCGGCGTTGGCGATTTTAAGCGCGGCAGCGCAACTGGGTACGACG<br>GTGGAGGAGGCGGTTAATCGCGCGTTGAATTTGAAGACGAAGTTGGGGGTGGATTTGGTTACCGCGGCTTTAGCCCTGCTGACCGCAGCT<br>AAACTAGGCACTACAGTCGAGGAAGCTGTACAGGAGGCTTTGCAGCTTAAAACTAAACTAGGCGTTTCACTGATAGAAGCATTGCATATC<br>CTGTTGACGGCCGCAGTACTGGGGACAACTGTAGAAGAGGCTGTATATCGAGCGCTTAAACTCAAGACAAAGCTAGGGGTTAGTTTGCTC<br>CAGGCAGCGGCCATATTGATTTTAGCAGCGCGCCTGGGGACTACAGTAGAGCAGGCTGTAGAACGCGCATTGCAACTAAAGACTAAGCTT<br>GGTGGCGGGAGCGGTGGCTCCCATCATTGGGGCAGCGGCAGCCTCGAGCACCACCACCACCACCAC
**dnHEM1-SS30**	
AA sequence:	MVSLDIAILILVVAAKLGTTVEEAVQQALWLKTKLGVSLIQALLILAAAANTGTTVEEAVQEALQLKTKLGVSLEAALAILSAAAQLGTT<br>VEEAVNRALNLKTKLGVDLLTAALALLTAAKLGTTVEEAVQEALQLKTKLGVSLIEAL**H**ILLTAAVLGTTVEEAVYRALKLKTKLGVSLL<br>QAAAILILAARLGTTVEQAVERALQLKTKLGGGSGG*SHHWGSGSLEHHHHHH*
DNA sequence:	ATGGTGAGCCTGGATATTGCGATTCTGATTCTGGTGGTGGCGGCGAAACTGGGCACCACCGTGGAAGAAGCGGTGCAGCAGGCGCTGTGG<br>CTGAAAACCAAATTAGGCGTGAGTTTGATTCAGGCACTGCTGATCCTGGCCGCCGCTGCCAATACCGGCACGACGGTTGAAGAGGCCGTC<br>CAGGAAGCGCTGCAGTTGAAAACGAAGTTGGGTGTGAGCTTGGAAGCGGCGTTGGCGATTTTAAGCGCGGCAGCGCAACTGGGTACCACG<br>GTGGAGGAGGCAGTTAATCGCGCGTTGAATTTGAAGACGAAATTGGGGGTGGATTTGTTAACCGCGGCTTTAGCCCTGCTGACCGCAGCT<br>AAACTAGGCACTACAGTCGAGGAAGCTGTACAGGAGGCTTTGCAGCTTAAAACTAAACTAGGCGTTTCACTGATAGAAGCATTGCATATC<br>CTGTTGACGGCCGCAGTACTGGGGACAACTGTAGAAGAGGCTGTATATCGAGCGCTTAAACTCAAGACAAAGCTAGGGGTTAGTTTGCTC<br>CAGGCAGCGGCCATATTGATTTTAGCAGCGCGCCTGGGGACTACAGTAGAGCAGGCTGTAGAACGCGCATTGCAACTAAAGACTAAGCTT<br>GGTGGCGGGAGCGGTGGCTCCCATCATTGGGGCAGCGGCAGCCTCGAGCACCACCACCACCACCAC
**dnHEM1-SS31**	
AA sequence:	MVSLDEAILILVVAAKLGTTVEEAVQQALWLKTKLGVSLLQALLILAAAANTGTTVEEAVQEALQLKTKLGVSLEAALAILSAAAQLGTT<br>VEEAVNRALNLKTKLGVDLATAALALLTAAKLGTTVEEAVQEALQLKTKLGVSLIEAL**H**ILLTAAVLGTTVEEAVYRALKLKTKLGVSLL<br>QAAAILILAARLGTTVEQAVERALQLKTKLGGGSGG*SHHWGSGSLEHHHHHH*
DNA sequence:	ATGGTGAGCCTGGATGAAGCGATTCTGATTCTGGTGGTGGCGGCGAAACTGGGCACCACCGTGGAAGAAGCGGTGCAGCAGGCGCTGTGG<br>CTGAAAACCAAATTAGGCGTGAGTCTGCTGCAGGCGTTGTTGATTCTGGCCGCCGCCGCCAATACCGGTACCACGGTTGAAGAGGCCGTT<br>CAAGAAGCCTTGCAATTGAAAACGAAGTTGGGTGTTAGCTTGGAAGCGGCGTTGGCGATTTTAAGCGCGGCGGCGCAATTGGGTACGACC<br>GTCGAGGAGGCAGTTAATCGCGCGTTGAATTTGAAGACCAAGCTGGGCGTGGATTTAGCGACCGCGGCTTTAGCCCTGCTGACCGCAGCT<br>AAACTAGGCACTACAGTCGAGGAAGCTGTACAGGAGGCTTTGCAGCTTAAAACTAAACTAGGCGTTTCACTGATAGAAGCATTGCATATC<br>CTGTTGACGGCCGCAGTACTGGGGACAACTGTAGAAGAGGCTGTATATCGAGCGCTTAAACTCAAGACAAAGCTAGGGGTTAGTTTGCTC<br>CAGGCAGCGGCCATATTGATTTTAGCAGCGCGCCTGGGGACTACAGTAGAGCAGGCTGTAGAACGCGCATTGCAACTAAAGACTAAGCTT<br>GGTGGCGGGAGCGGTGGCTCCCATCATTGGGGCAGCGGCAGCCTCGAGCACCACCACCACCACCAC
**dnHEM1-SS32**	
AA sequence:	MVSLDIAILILVVAAKLGTTVEEAVQQALWLKTKLGVSLLQALVILAAAANTGTTVEEAVQEALQLKTKLGVSLFEALAILSAAAQLGTT<br>VEEAVNRALNLKTKLGVDLATAALALLTAAKLGTTVEEAVQEALQLKTKLGVSLIEAL**H**ILLTAAVLGTTVEEAVYRALKLKTKLGVSLL<br>QAAAILILAARLGTTVEEAVQEALQLKTKLGGGSGG*SHHWGSGSLEHHHHHH*
DNA sequence:	ATGGTGAGCCTGGATATTGCGATTTTAATTTTAGTTGTTGCGGCGAAATTAGGTACCACCGTTGAAGAAGCGGTTCAACAAGCGCTGTGG<br>CTGAAAACCAAATTGGGCGTGAGTCTGCTGCAGGCGTTGGTTATTCTGGCCGCCGCTGCCAATACCGGTACGACGGTTGAGGAGGCCGTT<br>CAAGAAGCCTTGCAATTAAAAACTAAACTGGGTGTTAGCTTATTTGAAGCGTTAGCGATCTTAAGCGCGGCGGCGCAATTAGGCACTACT<br>GTAGAAGAGGCGGTTAATCGTGCGTTGAATTTAAAGACGAAGCTCGGGGTGGATTTAGCGACCGCGGCGCTGGCGTTATTGACGGCGGCC<br>AAGCTTGGTACGACGGTCGAGGAAGCAGTCCAGGAAGCCCTGCAGTTAAAGACGAAGCTGGGGGTGAGCTTGATTGAGGCCCTGCATATT<br>TTGCTGACTGCCGCGGTGTTGGGCACGACTGTCGAAGAGGCCGTGTATCGCGCCTTGAAACTGAAGACTAAGCTTGGTGTCAGTTTACTT<br>CAAGCAGCGGCAATTCTCATCTTAGCGGCGCGTCTGGGGACCACGGTGGAGGAGGCTGTGCAGGAGGCATTGCAGCTCAAAACCAAGTTA<br>GGTGGGGGCTCGGGTGGGAGCCATCATTGGGGTAGCGGTAGCCTCGAGCACCACCACCACCACCAC
**dnHEM1-SS33**	
AA sequence:	MVSLDIAIIILVAAAKLGTTVEEAVQQALWLKTKLGVSLIQALIILLAAANTGTTVEEAVQEALQLKTKLGVSLFEALAILSAAAQLGTT<br>VEEAVNRALNLKTKLGVDLATAALALLTAAKLGTTVEEAVQEALQLKTKLGVSLIEAL**H**ILLTAAVLGTTVEEAVYRALKLKTKLGVSLL<br>QAAAILILAARLGTTVEEAVQEALQLKTKLGGGSGG*SHHWGSGSLEHHHHHH*
DNA sequence:	ATGGTGAGCCTGGATATTGCGATTATTATTTTAGTTGCGGCGGCGAAATTGGGCACCACCGTGGAAGAAGCGGTGCAGCAGGCGCTGTGG<br>CTGAAAACCAAATTAGGTGTGAGTTTGATTCAAGCACTGATTATTTTATTAGCAGCCGCCAATACCGGTACGACTGTTGAAGAGGCCGTG<br>CAGGAAGCGCTGCAGCTGAAGACCAAGTTGGGTGTAAGTCTGTTTGAAGCCCTGGCGATTTTAAGCGCGGCAGCGCAGCTGGGTACCACG<br>GTAGAGGAAGCAGTGAATCGTGCGCTGAATCTGAAGACGAAATTGGGCGTGGATTTAGCGACCGCGGCGTTAGCGTTATTGACGGCGGCT<br>AAGCTCGGCACGACGGTTGAGGAGGCTGTTCAGGAAGCGTTGCAATTGAAAACCAAGCTGGGGGTTAGCTTGATTGAAGCTTTGCATATC<br>TTGCTGACCGCCGCGGTGTTGGGTACTACCGTGGAAGAAGCAGTGTATCGTGCCTTGAAATTAAAAACTAAACTTGGTGTTAGTCTGCTG<br>CAGGCCGCTGCCATCTTGATTTTAGCGGCGCGCCTGGGGACTACGGTGGAGGAGGCTGTCCAAGAAGCCTTGCAGTTAAAGACTAAGTTA<br>GGTGGTGGCAGCGGTGGTAGCCATCATTGGGGTAGCGGTAGCCTCGAGCACCACCACCACCACCAC
**dnHEM1-SS34**	
AA sequence:	MVSLDQAILILIVAAKLGTTVEEAVQQALWLKTKLGVSLLQALLILAAAANTGTTVEEAVQEALQLKTKLGVSLEAALAILSAAAQLGTT<br>VEEAVNRALNLKTKLGVDLVTAALALLTAAKLGTTVEEAVQEALQLKTKLGVSLIEAL**H**ILLTAAVLGTTVEEAVYRALKLKTKLGVSLL<br>QAAAILILAARLGTTVEQAVERALQLKTKLGGGSGG*SHHWGSGSLEHHHHH*
DNA sequence:	ATGGTGAGCCTGGATCAGGCGATTCTGATCCTGATTGTGGCGGCGAAACTGGGCACCACCGTGGAAGAAGCGGTGCAGCAGGCGCTGTGG<br>CTGAAAACCAAATTAGGCGTGAGTCTGCTGCAAGCGCTGCTGATTCTGGCCGCCGCTGCCAATACCGGTACCACGGTTGAAGAGGCCGTT<br>CAAGAGGCGTTGCAATTGAAAACGAAGTTGGGTGTTAGCTTGGAAGCGGCGTTGGCGATTTTAAGCGCGGCGGCGCAATTGGGTACGACC<br>GTTGAGGAGGCAGTTAATCGCGCGTTGAATTTGAAGACGAAGCTCGGCGTGGATTTGGTTACCGCGGCTTTAGCCCTGCTGACCGCAGCT<br>AAACTAGGCACTACAGTCGAGGAAGCTGTACAGGAGGCTTTGCAGCTTAAAACTAAACTAGGCGTTTCACTGATAGAAGCATTGCATATC<br>CTGTTGACGGCCGCAGTACTGGGGACAACTGTAGAAGAGGCTGTATATCGAGCGCTTAAACTCAAGACAAAGCTAGGGGTTAGTTTGCTC<br>CAGGCAGCGGCCATATTGATTTTAGCAGCGCGCCTGGGGACTACAGTAGAGCAGGCTGTAGAACGCGCATTGCAACTAAAGACTAAGCTT<br>GGTGGCGGGAGCGGTGGCTCCCATCATTGGGGCAGCGGCAGCCTCGAGCACCACCACCACCAC

**dnHEM1-SS35**	
AA sequence:	MVSLDEAILILVVAAKLGTTVEEAVQQALWLKTKLGVSLLQALAILAAAANTGTTVEEAVQEALQLKTKLGVSLLAALAILSAAAQLGTT<br>VEEAVNRALNLKTKLGVDLATAALALLTAAKLGTTVEEAVQEALQLKTKLGVSLIEAL**H**ILLTAAVLGTTVEEAVYRALKLKTKLGVSLL<br>QAAAILILAARLGTTVEEAVERALQLKTKLGGGSGG*SHHWGSGSLEHHHHHH*
DNA sequence:	ATGGTGAGCCTGGATGAAGCGATTCTGATTCTGGTGGTGGCGGCGAAACTGGGCACCACCGTGGAAGAAGCGGTGCAGCAGGCGCTGTGG<br>CTGAAAACCAAATTAGGCGTGAGTCTGCTGCAGGCGTTGGCGATTTTGGCCGCCGCTGCCAATACCGGTACCACGGTTGAAGAGGCCGTT<br>CAAGAAGCCTTGCAATTGAAAACGAAGTTGGGTGTTAGCTTGTTGGCGGCCCTGGCCATCTTAAGCGCGGCGGCGCAACTGGGTACGACT<br>GTCGAGGAGGCGGTTAATCGTGCGTTGAATTTGAAGACCAAGCTGGGGGTGGATTTAGCGACCGCGGCTTTAGCCCTGCTGACCGCCGCC<br>AAGCTCGGTACGACCGTAGAAGAGGCTGTTCAAGAAGCTCTGCAATTAAAAACTAAATTAGGCGTAAGCTTGATTGAAGCCTTGCATATT<br>CTGTTGACGGCCGCGGTGCTGGGGACCACTGTCGAAGAAGCAGTGTATCGTGCGCTGAAACTGAAGACCAAGCTGGGGGTCAGTCTGCTG<br>CAGGCAGCGGCGATCTTGATTTTAGCGGCGCGCCTGGGCACCACTGTGGAGGAGGCTGTTGAACGCGCATTGCAATTGAAGACTAAACTC<br>GGTGGCGGGAGCGGTGGCTCCCATCATTGGGGCAGCGGCAGCCTCGAGCACCACCACCACCACCAC
**dnHEM1-SS36**	
AA sequence:	MVSLDEAILILIVAAKLGTTVEEAVQQALWLKTKLGVSLLQALLILAAAANTGTTVEEAVQEALQLKTKLGVSLEAALVILSAAAQLGTT<br>VEEAVNRALNLKTKLGVDLVTAALALLTAAKLGTTVEEAVQEALQLKTKLGVSLIEAL**H**ILLTAAVLGTTVEEAVYRALKLKTKLGVSLL<br>QAAAILILAARLGTTVEQAVERALQLKTKLGGGSGG*SHHWGSGSLEHHHHHH*
DNA sequence:	ATGGTGAGCCTGGATGAAGCGATTCTGATCTTAATTGTGGCGGCGAAACTGGGCACCACCGTGGAAGAAGCGGTGCAGCAGGCGCTGTGG<br>CTGAAAACCAAATTAGGCGTGAGTCTGCTGCAAGCACTGCTGATTCTGGCCGCCGCTGCCAATACCGGTACCACGGTTGAAGAGGCCGTT<br>CAAGAGGCGTTGCAATTGAAAACGAAGTTGGGTGTTAGCTTGGAAGCGGCGTTGGTTATTTTAAGCGCGGCGGCGCAGCTGGGTACGACG<br>GTCGAGGAGGCAGTGAATCGCGCGCTGAATCTGAAGACGAAACTCGGGGTGGATTTGGTTACCGCGGCTTTAGCCCTGCTGACCGCAGCT<br>AAACTAGGCACTACAGTCGAGGAAGCTGTACAGGAGGCTTTGCAGCTTAAAACTAAACTAGGCGTTTCACTGATAGAAGCATTGCATATC<br>CTGTTGACGGCCGCAGTACTGGGGACAACTGTAGAAGAGGCTGTATATCGAGCGCTTAAACTCAAGACAAAGCTAGGGGTTAGTTTGCTC<br>CAGGCAGCGGCCATATTGATTTTAGCAGCGCGCCTGGGGACTACAGTAGAGCAGGCTGTAGAACGCGCATTGCAACTAAAGACTAAGCTT<br>GGTGGCGGGAGCGGTGGCTCCCATCATTGGGGCAGCGGCAGCCTCGAGCACCACCACCACCACCAC
**IK_HC015_1**	
AA sequence:	MVSLDQALTILSAAA**E**LGTTVEEAVKRALWLKTKLGVSLEQALKLL**H**AAAVLGTTVEEAVKRALKLKTKLGVSLEQALTILATAAALGTT<br>VEEAVKRALKLKTKLGVSLEQALWILAVAAALGTTVEEAVKRALKLKTKLGVSLEQALKILLVAALLGTTVEEAVYRALKLKTKLGVSLE<br>QALRILATAAVLGTTVEEAVKRALKLKTKLGGGSGG*SHHWGSGSHHHHHH*
DNA sequence:	GTGAGCCTGGATCAGGCGCTGACCATTCTGAGCGCGGCGGCGGAACTGGGCACCACCGTGGAAGAAGCGGTGAAACGCGCGCTGTGGCTG<br>AAAACCAAATTGGGCGTGAGCTTGGAACAGGCGTTGAAACTGCTGCATGCGGCCGCGGTGCTGGGTACGACGGTTGAAGAGGCCGTTAAA<br>CGTGCACTGAAGCTGAAGACGAAGCTCGGTGTCTCGTTGGAGCAAGCCTTGACGATTTTAGCGACCGCCGCGGCGTTGGGTACCACGGTA<br>GAGGAGGCTGTAAAGCGTGCTTTGAAGCTCAAGACGAAGTTGGGTGTTTCGTTAGAGCAGGCCTTATGGATTCTTGCGGTTGCCGCCGCC<br>TTGGGGACGACCGTTGAGGAAGCAGTGAAGCGCGCCTTGAAATTAAAAACTAAACTGGGGGTTAGCTTAGAACAAGCTCTGAAAATTCTG<br>CTGGTGGCCGCACTGTTAGGGACCACTGTGGAGGAGGCAGTGTATCGCGCATTGAAGTTGAAAACGAAATTAGGCGTGTCGCTGGAGCAA<br>GCCCTGCGTATTCTCGCCACGGCCGCCGTTTTGGGCACTACCGTCGAAGAGGCTGTCAAACGTGCCCTCAAACTGAAGACGAAATTGGGT<br>GGTGGCAGCGGTGGGAGCCATCATTGGGGCTCGGGGTCGCATCATCACCACCATCAT
**IK_HC015_3**	
AA sequence:	MVSLEQAAKFLATAAVLGTTVEEAVKRALWLKTKLGVSLEQALLLL**H**IAAALGTTVEEAVKRALKLKTKLGVSLEQALWILATAYALGTT<br>VEEAVKRALKLKTKLGVSLDQALTILLAAALLGTTVEEAVKRALKLKTKLGVSLKQALLFLQLAAQLGTTVEEAVYRALKLKTKLGVSLE<br>QAAIILAAASALGTTVEEAVKRALKLKTKLGGGSGG*SHHWGSGSHHHHHH*
DNA sequence:	GTGAGCCTGGAACAGGCGGCGAAATTTTTAGCGACCGCGGCGGTGCTGGGCACCACCGTGGAAGAAGCGGTGAAACGCGCGCTGTGGCTG<br>AAAACCAAACTGGGCGTGTCGTTGGAGCAGGCGCTGTTATTATTGCATATTGCGGCGGCGTTGGGCACGACGGTTGAAGAGGCCGTTAAA<br>CGTGCACTGAAACTGAAGACGAAGTTGGGTGTTTCTTTGGAACAAGCTCTGTGGATTCTGGCGACTGCGTATGCGTTAGGTACGACCGTA<br>GAGGAGGCTGTAAAGCGTGCCTTGAAGTTAAAGACCAAATTAGGCGTATCGCTGGATCAGGCACTGACCATTTTGTTAGCGGCTGCACTG<br>CTGGGTACCACGGTCGAAGAGGCAGTCAAGCGCGCATTGAAACTCAAGACGAAGCTGGGGGTTAGCCTGAAACAGGCCCTGCTGTTTCTG<br>CAGCTGGCCGCGCAATTGGGTACTACCGTCGAGGAAGCTGTGTATCGCGCCCTGAAATTAAAAACGAAACTTGGGGTGTCCTTGGAGCAG<br>GCCGCGATTATTTTGGCCGCCGCGAGCGCACTGGGGACTACTGTGGAGGAGGCCGTAAAACGTGCTTTGAAATTGAAAACTAAACTCGGT<br>GGTGGCAGCGGTGGGAGCCATCATTGGGGCAGTGGCTCGCATCATCACCACCATCAT
**IK_HC015_4**	
AA sequence:	MVSLDQAVIILAVARLLGTTVEEAVKRALWLKTKLGVSLDQALFIL**H**AAAITGTTVEEAVKRALKLKTKLGVSLEAALAILAAAAKLGTT<br>VEEAVKRALKLKTKLGVSLFVAALALATAAKLGTTVEEAVKRALKLKTKLGVSLKEALLILLTAARLGTTVEEAVYRALKLKTKLGVSLE<br>QALLILLLAAALGTTVEEAVKRALKLKTKLGGGSGG*SHHWGSGSHHHHHH*
DNA sequence:	GTGAGCCTGGATCAGGCGGTGATTATTTTAGCGGTGGCGCGCCTGCTGGGCACCACCGTGGAAGAAGCGGTGAAACGCGCGCTGTGGCTG<br>AAAACCAAACTGGGCGTGTCTTTGGACCAGGCGCTGTTTATTCTGCATGCGGCGGCGATTACCGGCACGACGGTTGAGGAGGCCGTTAAG<br>CGTGCACTGAAACTGAAGACGAAGTTGGGTGTATCGTTGGAAGCGGCGCTGGCGATTCTGGCGGCCGCCGCGAAATTGGGTACGACTGTG<br>GAGGAGGCCGTCAAACGCGCGTTGAAATTGAAAACGAAATTAGGGGTGAGCTTGTTTGTTGCGGCGTTAGCGTTGGCGACCGCGGCTAAG<br>CTCGGTACTACTGTCGAAGAGGCGGTTAAACGTGCCTTGAAGTTAAAGACCAAGCTGGGTGTGAGTCTGAAAGAAGCCCTGCTGATTTTG<br>TTAACCGCAGCCCGCCTGGGTACCACGGTGGAAGAGGCAGTGTACCGCGCCCTGAAGTTGAAGACTAAGTTAGGCGTTTCTCTGGAACAG<br>GCGTTGTTGATCTTGTTATTAGCGGCAGCCTTGGGGACCACTGTTGAAGAGGCTGTAAAGCGCGCATTAAAATTAAAAACCAAACTCGGT<br>GGTGGCAGCGGGGGGAGCCATCATTGGGGCAGTGGCTCTCATCATCACCACCATCAT
**IK_HC015_5**	
AA sequence:	MVSLEQAAEFLATAAKLGTTVEEAVKRALWLKTKLGVSLEQAVLLL**H**IAAVLGTTVEEAVKRALKLKTKLGVSLEQALTILATAWSLGTT<br>VEEAVKRALKLKTKLGVSLDQALWILIAAASLGTTVEEAVKRALKLKTKLGVSLKQALWFLILAAQLGTTVEEAVYRALKLKTKLGVSLE<br>QAAKILAAAAALGTTVEEAVKRALKLKTKLGGGSGG*SHHWGSGSHHHHHH*
DNA sequence:	GTGAGCCTGGAACAGGCGGCGGAATTTCTGGCGACCGCGGCGAAACTGGGCACCACCGTGGAAGAAGCGGTGAAACGCGCGCTGTGGCTG<br>AAAACCAAATTAGGCGTGTCTTTGGAGCAAGCCGTGTTGTTGCTGCATATTGCGGCGGTGCTGGGTACGACGGTAGAGGAGGCCGTTAAA<br>CGTGCACTGAAACTGAAGACGAAGTTGGGCGTATCTCTTGAGCAGGCGCTGACCATTCTGGCCACTGCGTGGTCGTTGGGGACCACTGTG<br>GAGGAAGCAGTGAAGCGTGCTTTGAAGTTGAAGACCAAGCTGGGTGTTTCGTTGGATCAGGCGTTATGGATTCTGATTGCCGCCGCGAGT<br>CTGGGCACCACCGTTGAGGAAGCTGTAAAGCGCGCATTGAAATTGAAAACGAAATTGGGTGTTAGCCTGAAACAGGCCCTGTGGTTTTTG

	ATTTTAGCGGCGCAGTTAGGGACTACCGTCGAAGAGGCGGTGTATCGCGCCCTCAAGCTCAAGACTAAACTCGGCGTGTCTCTGGAGCAG<br>GCCGCAAAAATTTTGGCCGCTGCTGCCGCGTTAGGCACGACTGTCGAAGAGGCAGTAAAACGCGCTCTCAAATTGAAGACCAAACTTGGT<br>GGTGGCAGCGGCGGCAGCCATCATTGGGGCTCGGGGTCGCATCATCACCACCATCAT
**IK_HC015_6**	
AA sequence:	MVSLEQAARFLATAALLGTTVEEAVKRALWLKTKLGVSLEQAVLLL**H**IAANLGTTVEEAVKRALKLKTKLGVSLEQALNILATANALGTT<br>VEEAVKRALKLKTKLGVSLDQALWILIAAARLGTTVEEAVKRALKLKTKLGVSLKQALLFLILAAALGTTVEEAVYRALKLKTKLGVSLE<br>QAALILLAAAALGTTVEEAVKRALKLKTKLGGGSGG*SHHWGSGSHHHHHH*
DNA sequence:	GTGAGCCTGGAACAGGCGGCGCGCTTTTTAGCGACCGCGGCGCTGCTGGGCACCACCGTGGAAGAAGCGGTGAAACGCGCGCTGTGGCTG<br>AAAACCAAACTGGGTGTTAGCTTGGAACAAGCCGTGTTGCTGTTGCATATTGCGGCGAATCTGGGTACGACGGTTGAGGAGGCCGTTAAG<br>CGTGCGCTGAAACTGAAGACGAAGTTGGGCGTGTCGTTGGAGCAGGCGTTGAATATTCTGGCGACTGCGAATGCGTTGGGTACCACTGTG<br>GAGGAAGCAGTCAAACGTGCCTTGAAGTTGAAGACCAAGCTGGGTGTTTCTTTGGATCAGGCCTTATGGATTCTGATTGCCGCCGCGCGC<br>CTGGGGACCACGGTAGAGGAGGCCGTAAAGCGCGCCCTCAAGCTCAAGACTAAATTAGGTGTAAGCCTGAAACAGGCTTTACTGTTTTTG<br>ATTCTCGCGGCCGCGCTGGGCACTACCGTTGAGGAGGCTGTGTATCGCGCACTGAAATTGAAAACTAAGCTTGGTGTCAGTCTGGAGCAA<br>GCTGCCCTGATTTTATTAGCCGCAGCCGCCCTCGGTACGACTGTCGAAGAGGCTGTTAAACGTGCATTAAAATTAAAAACGAAATTGGGT<br>GGTGGCAGCGGCGGCAGCCATCATTGGGGCTCGGGGTCGCATCATCACCACCATCAT
**IK_HC015_7**	
AA sequence:	MVSLLQALWILIVAAKLGTTVEEAVKRALWLKTKLGVSLDQALRILLAAANTGTTVEEAVKRALKLKTKLGVSLEAALAILAAAAQLGTT<br>VEEAVKRALKLKTKLGVSLETAALALKTAALLGTTVEEAVKRALKLKTKLGVSLIEAL**H**ILITAAVLGTTVEEAVYRALKLKTKLGVSLI<br>QAAAILITAALLGTTVEEAVKRALKLKTKLGGGSGG*SHHWGSGSHHHHHH*
DNA sequence:	GTGAGCCTGCTGCAGGCGCTGTGGATTCTGATTGTGGCGGCGAAACTGGGCACCACCGTGGAAGAAGCGGTGAAACGCGCGTTATGGCTG<br>AAAACCAAATTAGGCGTGAGTCTGGATCAGGCACTGCGCATTCTGCTGGCCGCCGCCAATACCGGCACGACGGTTGAAGAGGCCGTTAAA<br>CGTGCGCTGAAACTGAAGACGAAGTTGGGTGTTTCGTTGGAAGCGGCGCTGGCGATTTTAGCGGCCGCAGCGCAGCTGGGTACCACGGTG<br>GAGGAGGCGGTCAAGCGCGCCTTGAAATTGAAAACGAAGCTGGGCGTCAGCTTGGAAACCGCGGCGTTGGCGTTAAAGACTGCGGCCCTG<br>TTGGGTACGACCGTTGAGGAAGCAGTGAAGCGTGCCCTGAAGCTCAAAACTAAATTGGGGGTAAGCTTGATTGAAGCGCTGCATATTTTA<br>ATTACCGCCGCGGTGTTAGGTACTACCGTAGAGGAGGCCGTGTATCGCGCCCTCAAGCTTAAGACTAAACTCGGCGTTAGTCTGATTCAG<br>GCAGCGGCGATCTTGATCACGGCCGCCTTGCTCGGGACGACTGTGGAAGAAGCTGTTAAGCGTGCACTGAAGCTCAAAACCAAGTTGGGT<br>GGTGGCTCGGGCGGCAGCCATCATTGGGGCAGTGGTTCGCATCATCACCACCATCAT
**IK_HC015_9**	
AA sequence:	VSLEQALKILAVAAWLGTTVEEAVKRALWLKTKLGVSLEQALRLLANAAALGTTVEEAVKRALKLKTKLGVSLEQALLILLVAAKLGTTV<br>EEAVKRALKLKTKLGVSLEQALKIL**H**AAAALGTTVEEAVKRALKLKTKLGVSLLQAIKILAVARLLGTTVEEAVYRALKLKTKLGVSLEQ<br>ALLILFTAAVLGTTVEEAVKRALKLKTKLGGGSGG*SHHWGSGSHHHHHH*
DNA sequence:	GTGAGCCTGGAACAGGCGCTGAAAATTTTAGCGGTGGCGGCGTGGCTGGGCACCACCGTGGAAGAAGCGGTGAAACGCGCGCTGTGGTTG<br>AAAACCAAACTGGGCGTGTCTTTGGAGCAAGCACTGCGTTTATTGGCGAATGCGGCCGCGTTGGGTACCACGGTTGAAGAGGCCGTTAAA<br>CGTGCGTTGAAATTGAAGACGAAGTTGGGTGTTAGCTTGGAACAAGCCCTGCTGATTCTGCTGGTTGCAGCGAAATTAGGTACGACGGTG<br>GAGGAGGCGGTTAAGCGTGCCTTAAAACTGAAGACTAAATTGGGGGTTTCGTTGGAGCAAGCCTTGAAGATCTTGCATGCGGCAGCAGCG<br>CTGGGGACGACTGTGGAGGAGGCAGTCAAACGTGCTTTGAAGCTGAAAACGAAGCTGGGTGTCAGTCTGCTGCAGGCGATTAAAATCCTC<br>GCCGTGGCGCGCTTGCTGGGTACTACCGTCGAGGAAGCCGTGTATCGCGCCCTGAAACTTAAAACTAAGTTAGGCGTTTCGCTTGAGCAG<br>GCTTTGTTGATCCTGTTTACCGCGGCGGTTTTAGGGACTACGGTTGAGGAGGCAGTAAAGCGCGCGTTAAAATTAAAAACGAAACTGGGT<br>GGTGGCAGCGGCGGGAGCCATCATTGGGGCTCGGGGTCGCATCATCACCACCATCAT
**IK_HC015_11**	
AA sequence:	VSLDQALLILAAAAVLGTTVEEAVKRALWLKTKLGVSLEQALWLLAEAAILGTTVEEAVKRALKLKTKLGVSLEQALKILAVAAKLGTTV<br>EEAVKRALKLKTKLGVSLEQALWILFVAAAAGTTVEETVKRALKLKTKLGVSLEQALLILAVANLLGTTVEEAVYRALKLKTKLGVSLEQ<br>ALLIL**H**TAALLGTTVEEAVKRALKLKTKLGGGSGG*SHHWGSGSHHHHHH*
DNA sequence:	GTGAGCCTGGATCAGGCGCTGCTGATTTTAGCGGCGGCCGCGGTGCTGGGCACCACCGTGGAAGAAGCGGTGAAACGCGCGCTGTGGCTG<br>AAAACCAAACTGGGCGTGAGCTTGGAACAGGCGTTGTGGTTATTGGCGGAAGCGGCGATTCTGGGTACGACGGTTGAAGAGGCCGTTAAA<br>CGTGCGCTGAAACTGAAGACGAAGCTCGGTGTGTCGTTGGAGCAAGCACTCAAAATTCTGGCGGTGGCGGCGAAATTGGGTACCACTGTC<br>GAGGAGGCGGTAAAGCGTGCCTTGAAGCTGAAAACGAAGTTGGGCGTTTCGTTAGAGCAGGCCTTATGGATTCTGTTTGTTGCGGCCGCT<br>GCGGGCACTACTGTAGAAGAAACCGTGAAGCGCGCACTGAAGTTAAAGACCAAATTAGGTGTATCGCTGGAACAAGCCCTCCTGATCTTG<br>GCCGTGGCGAATCTGCTGGGGACCACGGTCGAAGAGGCAGTGTACCGCGCTTTGAAATTAAAAACTAAACTCGGCGTGTCTTTGGAGCAA<br>GCTCTCTTGATTCTGCATACCGCGGCGCTCTTGGGGACGACTGTGGAGGAAGCGGTAAAACGTGCATTAAAATTGAAAACGAAGCTGGGT<br>GGTGGCAGCGGGGGCAGCCATCATTGGGGCAGTGGCTCGCATCATCACCACCATCAT
**IK_HC015_12**	
AA sequence:	VSLEQALLIIAVAAKLGTTVEEAVKRALWLKTKLGVSLEQALLLL**H**NAAVIGTTVEEAVKRALKLKTKLGVSLEQALKILAVAAALGTTV<br>EEAVKRALKLKTKLGVSLEQALFILAVAAILGTTVEEAVKRALKLKTKLGVSLEQALKILVAAYKLGTTVEEAVYRALKLKTKLGVSLEQ<br>ALLILLTAASLGTTVEEAVKRALKLKTKLGGGSGG*SHHWGSGSHHHHHH*
DNA sequence:	GTGAGCCTGGAACAGGCGCTGCTGATTATTGCGGTGGCGGCGAAACTGGGCACCACCGTGGAAGAAGCGGTGAAACGCGCGCTGTGGCTG<br>AAAACCAAATTAGGCGTGTCGTTGGAGCAAGCCCTGCTGTTGCTGCATAATGCGGCGGTGATTGGCACGACGGTTGAGGAGGCCGTTAAG<br>CGTGCACTGAAACTGAAGACGAAGTTGGGTGTTTCGCTGGAGCAGGCATTGAAAATTTTAGCGGTTGCCGCGGCGCTGGGTACGACCGTC<br>GAAGAGGCAGTCAAACGTGCCCTCAAGTTGAAGACCAAGCTGGGGGTCTCTTTGGAACAAGCACTGTTTATTCTGGCGGTCGCCGCGATT<br>CTGGGGACCACGGTGGAGGAAGCTGTTAAACGTGCCTTGAAGTTAAAAACGAAATTGGGCGTCTCGCTCGAACAAGCTTTGAAAATTCTG<br>GTTGCGGCGTATAAACTCGGTACCACCGTAGAGGAGGCAGTGTATCGCGCATTAAAGTTGAAGACTAAATTAGGGGTTAGCCTTGAGCAA<br>GCCTTGTTAATTCTGCTGACCGCCGCGTCGTTAGGTACTACTGTCGAGGAGGCAGTGAAGCGTGCTCTGAAGCTTAAAACCAAACTCGGT<br>GGTGGCAGCGGGGGCAGCCATCATTGGGGCTCGGGCTCTCATCATCACCACCATCAT
**IK_HC015_13**	
AA sequence:	VSLDQALLIIAVAAKLGTTVEEAVKRALWLKTKLGVSLEQALLLL**H**EARVLGTTVEEAVKRALKLKTKLGVSLEQALQILAVAAALGTTV<br>EEAVKRALKLKTKLGVSLEQALKILAFAAILGTTVEEAVKRALKLKTKLGVSLEQALWILLVAASLGTTVEEAVYRALKLKTKLGVSLEQ<br>ALLALATAAALGTTVEEAVKRALKLKTKLGGGSGG*SHHWGSGSHHHHHH*
DNA sequence:	GTGAGCCTGGATCAGGCGCTGCTGATTATTGCGGTGGCGGCGAAACTGGGCACCACCGTGGAAGAAGCGGTGAAACGCGCGCTGTGGCTG<br>AAAACCAAATTAGGCGTGTCGTTGGAACAGGCTCTGCTGTTGCTGCATGAAGCGCGCGTGCTGGGTACCACGGTTGAAGAGGCCGTTAAA

	CGTGCACTGAAACTGAAGACGAAGTTGGGTGTTTCGCTTGAGCAAGCTCTGCAGATTTTAGCGGTTGCGGCGGCGTTGGGCACGACGGTG<br>GAGGAGGCTGTTAAGCGTGCCTTGAAGCTCAAGACCAAGCTCGGTGTATCGTTAGAGCAGGCATTGAAAATTCTGGCGTTTGCCGCGATT<br>CTGGGGACGACTGTGGAGGAGGCAGTCAAACGTGCTTTGAAGTTAAAGACTAAATTGGGGGTTAGTCTGGAACAAGCATTGTGGATTCTG<br>CTGGTCGCGGCGAGTTTGGGTACTACCGTAGAGGAAGCTGTGTACCGCGCCCTGAAATTAAAAACGAAGCTGGGCGTCTCGCTGGAGCAA<br>GCGTTGTTAGCGTTAGCGACCGCGGCCGCCCTCGGCACTACGGTCGAGGAGGCCGTTAAGCGCGCGTTGAAACTCAAAACTAAACTCGGT<br>GGTGGCAGCGGGGGCAGCCATCATTGGGGCAGTGGGTCGCATCATCACCACCATCAT
**IK_HC015_16**	
AA sequence:	VSLKQAASFLTVAALLGTTVEEAVKRALWLKTKLGVSLEQALKLL**H**IAAVIGTTVEEAVKRALKLKTKLGVSLEQALRILATAAQLGTTV<br>EEAVKRALKLKTKLGVSLDQALTILIAAALLGTTVEEAVKRALKLKTKLGVSLKQALFFLALAAALGTTVEEAVYRALKLKTKLGVSLEV<br>AAAILAAAAALGTTVEEAVKRALKLKTKLGGGSGG*SHHWGSGSHHHHHH*
DNA sequence:	GTGAGCCTGAAACAGGCGGCGAGCTTTCTGACCGTGGCGGCGCTGCTGGGCACCACCGTGGAAGAAGCGGTGAAACGCGCGCTGTGGCTG<br>AAAACCAAACTGGGCGTGAGCTTAGAACAAGCGTTGAAACTGCTGCATATTGCGGCGGTGATTGGCACGACGGTTGAGGAGGCCGTTAAG<br>CGTGCGCTGAAGCTGAAGACGAAGTTGGGTGTCAGCCTGGAACAGGCCCTGCGCATTTTAGCGACCGCGGCGCAGCTGGGTACGACTGTG<br>GAGGAAGCAGTCAAACGTGCCTTGAAGTTGAAGACTAAATTAGGGGTTTCGTTGGATCAGGCACTGACCATTCTGATTGCCGCCGCCTTG<br>TTAGGCACTACCGTTGAGGAGGCTGTAAAGCGCGCTTTGAAACTGAAGACGAAGCTCGGCGTTTCTTTGAAGCAAGCACTGTTTTTTCTC<br>GCGTTAGCGGCTGCATTGGGTACTACGGTCGAAGAGGCGGTGTATCGCGCATTAAAATTAAAAACTAAGTTAGGCGTGTCGCTGGAAGTG<br>GCCGCAGCGATTCTGGCTGCAGCTGCCGCTTTGGGGACCACGGTGGAAGAAGCGGTGAAGCGTGCCCTCAAATTGAAAACGAAATTAGGT<br>GGTGGCAGCGGCGGCAGCCATCATTGGGGCAGTGGCTCGCATCATCACCACCATCAT
**IK_HC015_17**	
AA sequence:	VSLEQAAVILAVAARLGTTVEEAVKRALWLKTKLGVSLKQALLLL**H**IAAALGTTVEEAVKRALKLKTKLGVSLKQALTILATAAQLGTTV<br>EEAVKRALKLKTKLGVSLDQALTILIAAALLGTTVEEAVKRALKLKTKLGVSLKQALLFLQLAAALGTTVEEAVYRALKLKTKLGVSLEQ<br>AAKILAAAAALGTTVEEAVKRALKLKTKLGGGSGG*SHHWGSGSHHHHHH*
DNA sequence:	GTGAGCCTGGAACAGGCGGCGGTGATTTTAGCGGTTGCGGCGCGCCTGGGCACCACCGTGGAAGAAGCGGTGAAACGCGCGCTGTGGCTG<br>AAAACCAAACTGGGCGTGAGCTTGAAACAAGCGCTGTTGTTATTGCATATTGCCGCGGCGCTGGGTACGACCGTAGAGGAGGCCGTTAAA<br>CGTGCACTGAAACTGAAGACGAAGCTCGGTGTGAGTCTGAAACAGGCACTGACCATTCTGGCGACCGCAGCGCAGTTGGGTACTACGGTT<br>GAGGAAGCCGTCAAGCGTGCTTTGAAGTTGAAGACCAAGTTAGGTGTTTCGTTGGATCAGGCCTTGACGATTCTGATTGCAGCCGCCCTG<br>TTAGGTACCACGGTCGAAGAGGCAGTGAAGCGCGCATTAAAATTGAAAACTAAATTGGGTGTGTCTCTGAAGCAAGCTCTGCTGTTTTTG<br>CAATTAGCGGCCGCCTTGGGGACCACTGTAGAAGAGGCAGTGTATCGCGCTCTGAAATTAAAAACGAAACTGGGCGTTTCGCTGGAGCAA<br>GCAGCGAAAATTCTCGCTGCAGCGGCAGCTTTGGGCACGACGGTGGAGGAGGCTGTTAAGCGCGCGTTAAAGCTCAAGACTAAATTGGGT<br>GGTGGCAGCGGGGGCAGCCATCATTGGGGCAGTGGGTCGCATCATCACCACCATCAT
**IK_HC015_19**	
AA sequence:	VSLEQAAWFLAIAAALGTTVEEAVKRALWLKTKLGVSLEQAVTLL**H**AAAKLGTTVEEAVKRALKLKTKLGVSLEQALLILAAAAELGTTV<br>EEAVKRALKLKTKLGVSLDQALTILVAAARLGTTVEEAVKRALKLKTKLGVSLKQALLFLITAASLGTTVEEAVYRALKLKTKLGVSLEQ<br>AALIAAAAAKLGTTVEEAVKRALKLKTKLGGGSGG*SHHWGSGSHHHHHH*
DNA sequence:	GTGAGCCTGGAACAGGCGGCGTGGTTTCTGGCGATTGCGGCGGCGCTGGGCACCACCGTGGAAGAAGCGGTGAAACGCGCGCTGTGGCTG<br>AAAACCAAACTGGGTGTTAGCTTGGAACAAGCGGTTACCTTGTTGCATGCCGCGGCGAAATTGGGTACGACGGTTGAAGAGGCCGTTAAA<br>CGTGCGCTGAAACTGAAGACGAAGCTGGGCGTGTCGTTGGAGCAGGCGCTGCTGATTTTAGCGGCCGCCGCGGAATTAGGTACTACCGTT<br>GAGGAGGCGGTTAAGCGTGCCTTGAAGCTGAAAACTAAACTCGGTGTTTCGCTGGATCAGGCACTGACCATTTTAGTTGCGGCAGCGCGC<br>CTGGGTACCACGGTAGAGGAAGCAGTGAAGCGCGCTTTGAAACTCAAGACCAAGTTGGGCGTTAGCCTGAAACAGGCTTTACTGTTTCTG<br>ATTACCGCAGCGTCGTTAGGGACCACTGTGGAGGAGGCGGTGTATCGCGCCCTGAAATTAAAAACGAAGTTAGGCGTCAGCTTAGAGCAA<br>GCCGCTCTGATCGCCGCAGCCGCTGCCAAGCTCGGCACGACTGTCGAAGAGGCTGTCAAGCGTGCGTTGAAACTCAAGACTAAACTTGGT<br>GGTGGCTCGGGTGGCAGCCATCATTGGGGCAGCGGCTCGCATCATCACCACCATCAT
**IK_HC015_22**	
AA sequence:	VSLEQALWILATAALLGTTVEEAVKRALWLKTKLGVSLEQAILLL**H**AAALLGTTVEEAVKRALKLKTKLGVSLEQALKILSVAAQLGTTV<br>EEAVKRALKLKTKLGVSLEQALFILAAAAILGTTVEEAVKRALKLKTKLGVSLKQALLILIVAAALGTTVEEAVYRALKLKTKLGVSLEQ<br>ALWILLTAADLGTTVEEAVKRALKLKTKLGGGSGG*SHHWGSGSHHHHHH*
DNA sequence:	GTGAGCCTGGAACAGGCGCTGTGGATTTTAGCGACCGCGGCGCTGCTGGGCACCACCGTGGAAGAAGCGGTGAAACGCGCGTTATGGCTG<br>AAAACCAAACTGGGCGTGTCGTTGGAGCAGGCGATTCTGTTGCTGCATGCGGCGGCCCTCTTGGGTACCACGGTTGAAGAGGCCGTTAAA<br>CGTGCACTGAAACTGAAGACGAAGTTGGGTGTTTCTTTGGAACAAGCACTCAAAATTCTGAGCGTGGCCGCGCAGTTAGGTACGACCGTC<br>GAGGAGGCAGTCAAGCGTGCTTTGAAGCTGAAAACTAAATTAGGCGTTAGCTTAGAACAAGCCCTGTTTATTCTGGCCGCCGCGGCCATT<br>CTGGGTACTACTGTCGAAGAGGCAGTTAAACGCGCCTTGAAATTAAAAACGAAACTCGGTGTTAGCCTGAAACAGGCTCTGCTGATTCTG<br>ATTGTTGCCGCTGCGTTAGGCACGACGGTGGAGGAAGCCGTGTATCGCGCATTAAAACTCAAGACCAAGCTCGGTGTGAGCTTGGAACAA<br>GCTTTGTGGATTCTGCTGACGGCAGCGGATTTGGGCACTACCGTTGAGGAGGCTGTTAAGCGCGCCCTGAAATTGAAAACTAAGTTAGGT<br>GGTGGCTCGGGTGGCAGCCATCATTGGGGCAGTGGCTCTCATCATCACCACCATCAT

2. Crystallographic data

Crystallization, refinement and model building

Protein sample for crystallography was prepared following the procedure outlined in section "Protein production and purification of in vitro loaded dnHEM1", on page S4. The holoprotein was purified using Ni-affinity and size exclusion chromatography. The C-terminal hexahistidine tag was left intact. The *holo* dnHEM1 was crystallized at 7 mg mL^{-1} in assay buffer (50 mM KPi, 200 mM NaCl, pH 7.2). Crystallization conditions for dnHEM1 were identified using the LMB screen (Molecular Dimensions). Crystals suitable for diffraction experiments were obtained by sitting drop vapor diffusion at 4 °C in 200 nL drops containing equal volumes of protein and crystallization solution. For dnHEM1 this contained 0.1M HEPES pH 7.7, 70% (4S)-2-methyl-2,4-pentanediol. The crystals were cryoprotected using paraffin oil and flash-cooled in liquid nitrogen. Data were collected on beamline iO3 (wavelength 0.9763 Å) at the Diamond Light Source Facility and reduced and scaled with Xia2. The resolution limit of 1.6 Å was determined via paired refinement in PDBREDO.[9] The dnHEM1 crystal structure was solved by molecular replacement using the PHASER program in the CCP4 suite[10] using the initial left-handed closed α-solenoid design as the starting model (PDB code: 4YXX).[11] The dnHEM1 models were completed by iterative cycles of manual model building and real space refinement using the program COOT and crystallographic refinement using PHENIX.refine.[12] The processing and final refinement statistics are presented in Supplementary Table 6. The dnHEM1 coordinates and structure factors have been deposited in the Protein Data Bank under accession number 8C3W.

Supplementary Table 6. X-ray diffraction and refinement statistics. Data for the outermost resolution shell are given in parenthesis.

	**dnHEM1**
PDB ascension number	8C3W
Wavelength (Å)	0.9763
Resolution range (Å)	41.66 – 1.6<br>(1.782 - 1.72)[a]
Space group	$P\ 2_1\ 2_1\ 2_1$
Unit cell dimensions<br>a, b, c, (Å)<br>α, β, γ (°)	 51.75, 65.16, 70.20<br>90, 90, 90
Total reflections	419315 (33686)
Unique reflections	31364 (2878)
Multiplicity	13.4 (11.7)
Completeness (%)	97.98 (92.02)
Mean I/sigma(I)	22.91 (1.33)
Wilson B-factor ($Å^2$)	26.80
R-merge	0.0561 (1.036)
R-meas	0.05836 (1.083)
R-pim	0.01589 (0.3091)
$CC_{1/2}$	1 (0.82)
CC*	1 (0.949)
Reflections used in refinement	31353 (2873)

Reflections used for R-free	1488 (129)
R-work	0.1827 (0.3229)
R-free[b]	0.2001 (0.3311)
CC (work)	0.958 (0.871)
CC (free)	0.952 (0.884)
Number of non-hydrogen atoms	1769
macromolecules	1574
ligands	158
solvent	115
Protein residues	211
RMS (bonds)	0.011
RMS (angles)	0.74
Ramachandran favoured (%)	99.04
Ramachandran allowed (%)	0.96
Ramachandran outliers (%)	0
Rotamer outliers (%)	0
Clashscore	4.24
Average B-factor ($Å^2$)	30.65

[a]Values in parentheses are for highest resolution shell. [b]R-free was calculated using ~5% of the data separate from the rest

3. Computational details

3.1. Heme binding site design

Matching heme into protein scaffolds

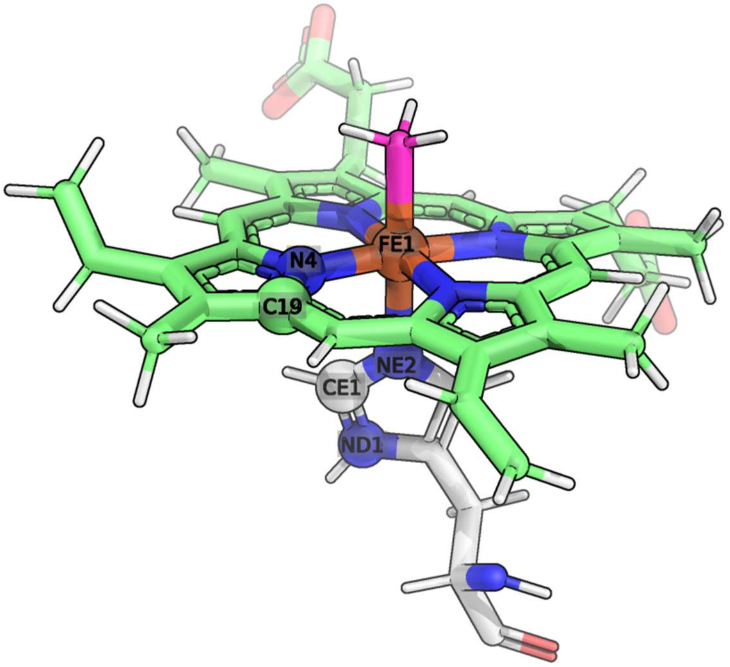

Supplementary Fig. 28. Fe-methyl heme model used in heme binding site matching and design. Atoms used for defining the heme-histidine constraints are labelled and shown as spheres. The added methyl group carbon atom is shown in magenta, heme carbon atoms are shown in green, and the coordinating histidine carbon atoms in white. Nitrogen = blue, oxygen = red, iron atom = orange, and hydrogen atoms are shown as small white sticks.

The heme model was built by adding a methyl group to the iron atom, *trans* to the coordinating imidazole ligand (acting as a mimic for histidine) and is depicted in Supplementary Fig. 28. The Rosetta params file, together with the rotamer library was created following the procedures described in section 3.2. The imidazole ligand was removed before creating the params file. A constraint file describing the heme-His interaction geometry was constructed based on the geometries found in native heme enzymes (peroxidases and globins), but allowing for flexible sampling of the torsional angle around the Fe-N bond. Unprotonated histidine nitrogen atom with Rosetta atom type 'NHis' was used for matching against heme iron atom. For steric reasons, only δ-protonated tautomer of histidine was used, and thus the three histidine atoms used for defining the constraints were: NE2, CE1, ND1 (Supplementary Fig. 28). Optionally, an additional hydrogen bonding residue (Glu or Asp) was matched downstream from His, to the protonated nitrogen atom, constrained to an idealized hydrogen bond geometry.

<u>Rosetta Matcher constraints used for matching heme and histidine:</u>

```
CST::BEGIN

  TEMPLATE::   ATOM_MAP: 1 atom_name: FE1 N4 C19
  TEMPLATE::   ATOM_MAP: 1 residue3:  HMM

  TEMPLATE::   ATOM_MAP: 2 atom_type: Nhis
  TEMPLATE::   ATOM_MAP: 2 residue3: HIS

  CONSTRAINT:: distanceAB:    2.09   0.1  100.0    1   1
  CONSTRAINT::    angle_A:   90.0   10.0    50.0  360.  1
  CONSTRAINT::    angle_B:  126.0   10.0    50.0  360.  1
  CONSTRAINT::  torsion_A:   90.0    5.0    25.0  360.  1
  CONSTRAINT:: torsion_AB:   51.4   20.0     0.0   90.  3
  CONSTRAINT::  torsion_B:  180.0    5.0    25.0  360.  1

  ALGORITHM_INFO:: match
     MAX_DUNBRACK_ENERGY 50.0
  ALGORITHM_INFO::END

CST::END
```

<u>Rosetta Matcher constraints used for matching heme, histidine and Glu/Asp:</u>

```
CST::BEGIN

  TEMPLATE::   ATOM_MAP: 1 atom_name: FE1 N4 C19
  TEMPLATE::   ATOM_MAP: 1 residue3:  HMM

  TEMPLATE::   ATOM_MAP: 2 atom_type: Nhis
  TEMPLATE::   ATOM_MAP: 2 residue3: HIS

  CONSTRAINT:: distanceAB:    2.09   0.1  100.     1  1
  CONSTRAINT::    angle_A:   90.0   10.0   50.0  360. 1
  CONSTRAINT::    angle_B:  126.0   10.0   50.0  360. 1
  CONSTRAINT::  torsion_A:   90.0    5.0   25.0  360. 1
  CONSTRAINT:: torsion_AB:   51.4   20.0    0.0   90. 3
  CONSTRAINT::  torsion_B:  180.0    5.0   25.0  360. 1

  ALGORITHM_INFO:: match
     MAX_DUNBRACK_ENERGY 50.0
  ALGORITHM_INFO::END

CST::END


# ED backing up HIS
CST::BEGIN
TEMPLATE::   ATOM_MAP: 1 atom_type: Ntrp
TEMPLATE::   ATOM_MAP: 1 residue3:  HIS

TEMPLATE::   ATOM_MAP: 2 atom_type: OOC
TEMPLATE::   ATOM_MAP: 2 residue1: ED

CONSTRAINT:: distanceAB:   2.66  0.2   100.  1    2
CONSTRAINT::    angle_A:  125.0  15.0   50.0  360. 3
CONSTRAINT::    angle_B:  108.9  25.0   50.0  180. 3
CONSTRAINT::  torsion_A:    0.0  25.0   50.0  180. 3
CONSTRAINT::  torsion_B:  180.0  20.0   50.0  360. 2

  ALGORITHM_INFO:: match
     SECONDARY_MATCH: UPSTREAM_CST 1
     MAX_DUNBRACK_ENERGY 50.0
  ALGORITHM_INFO::END

CST::END
```

A crystal structure of a toroidal repeat protein (PDB id: 4YXX) was relaxed using Rosetta FastRelax in multiple trajectories, and the resulting models were used as input scaffolds for Rosetta Matcher. Matching was restricted to positions inside the pore of the toroid: 4, 7, 8, 11, 12, 15, 39, 42, 43, 46, 47, 50, 74, 77, 78, 81, 82, 85, 109, 112, 113, 116, 117, 120, 144, 147, 148, 151, 152, 155, 179, 182, 183, 186, 187, 190 (defined in a file `donut.pos`).

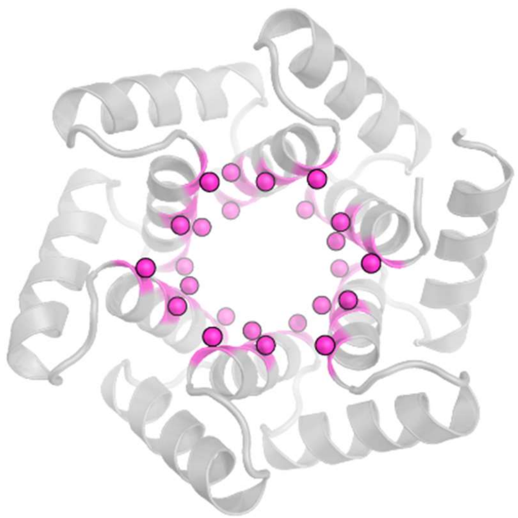

Supplementary Fig. 29. Positions in the toroid scaffold used for matching.

Matching was run using the following command and flags:

```
$ROSETTAPATH/main/source/bin/match.hdf5.linuxgccrelease @match.flags -s donut.pdb
-match:scaffold_active_site_residues donut.pos
```

Contents of the file `match.flags`:

```
-extra_res_fa /path/to/theozyme/HMM/HMM.params
-match::lig_name HMM
-match::dynamic_grid_refinement true
-match::enumerate_ligand_rotamers true
-match::consolidate_matches true
-match::output_matches_per_group 10
-in:ignore_unrecognized_res
-ex1
-ex2
-match:geometric_constraint_file /path/to/theozyme/HMM/HMM_onlyH.cst
```

Models obtained by running Rosetta 'match' application[13] with the aforementioned constraints and scaffolds were lastly evaluated for how solvent-exposed the placed heme molecule is. Structures where more than 20% of heme is exposed, based on calculating the solvent-accessible surface area (SASA) of the heme model were discarded. Matches were filtered using a Python script '`analyze_matches_Heme.py`' available for download on GitHub.[14]

Heme binding site design

The models obtained from the matching step were subjected to binding site sequence optimization using the Rosetta 'enzyme_design' application using the command and flags described below.[15] Positions within 8Å of any ligand heavy atom were set to be designable while positions within 12Å were allowed to be repacked. Iterative application of repacking, design and minimization steps, while applying the same constraints used in matching, yielded models that were then scored by metrics describing the His-Fe interaction geometry, shape-complementarity, and preorganization of the heme binding pocket (assessed by side chain packing calculations in the absence of the heme). The constraint score and no-ligand-repack RMSD metrics were calculated within the design protocol. Thereafter, designs passing thresholds for these metrics were evaluated for how solvent-exposed heme is, the shape complementarity between the heme and the ligand, and whether both of the

carboxylate groups of heme have at least on hydrogen bond partner. The latter three metrics, as well as preliminary filtering of the designs were implemented in a Python script 'analyze_scores_heme_enzdes_pdb.py' available for download on GitHub.[14] The thresholds for filtering are summarized in Supplementary Table 7 and the distribution of scores in Supplementary Fig. 30.

Design jobs were run using the following command and flags:

```
$ROSETTAPATH/main/source/bin/enzyme_design.hdf5.linuxgccrelease
@design.flags -packing:fix_his_tautomer 148 -s /path/to/UM_1_H148_donut_HMM_onlyH_1.pdb
-out:file:o scorefile.txt
```

Contents of the file `design.flags`:

```
-extra_res_fa /path/to/theozyme/HMM/HMM.params
-ex1
-ex2
-use_input_sc
-linmem_ig 10
-nstruct 5
-beta
-score:weights /path/to/beta16_nostab_nocart.wts
-enzdes
 -parser_read_cloud_pdb
 -cstfile /path/to/theozyme/HMM/HMM_onlyH.cst
 -detect_design_interface
 -cut1 6.0
 -cut2 8.0
 -cut3 10.0
 -cut4 12.0
 -cst_min
 -cst_opt
 -chi_min
 -cst_design
 -bb_min
 -design_min_cycles 3
 -lig_packer_weight 1.6
 -favor_native_res 0.8
 -start_from_random_rb_conf
 -final_repack_without_ligand
```

Supplementary Table 7. Thresholds used for filtering the heme binder design models.

Metric	Rosetta score name	Threshold
Heme-His (and His-Glu/Asp) constraint score	all_cst	<= 3.0
No-ligand-repack RMSD of catalytic residues (His and Glu/Asp)	nlr_rms_SR1<br>nlr_rms_SR2	<= 0.5
No-ligand-repack RMSD of all residues	nlr_rms_total	<= 0.8
Shape complementarity	sc	>= 0.6
Heme relative SASA (SASA_bound / SASA_free)	-	<= 0.2
Heme COO H-bond partners (True / False)	-	= True

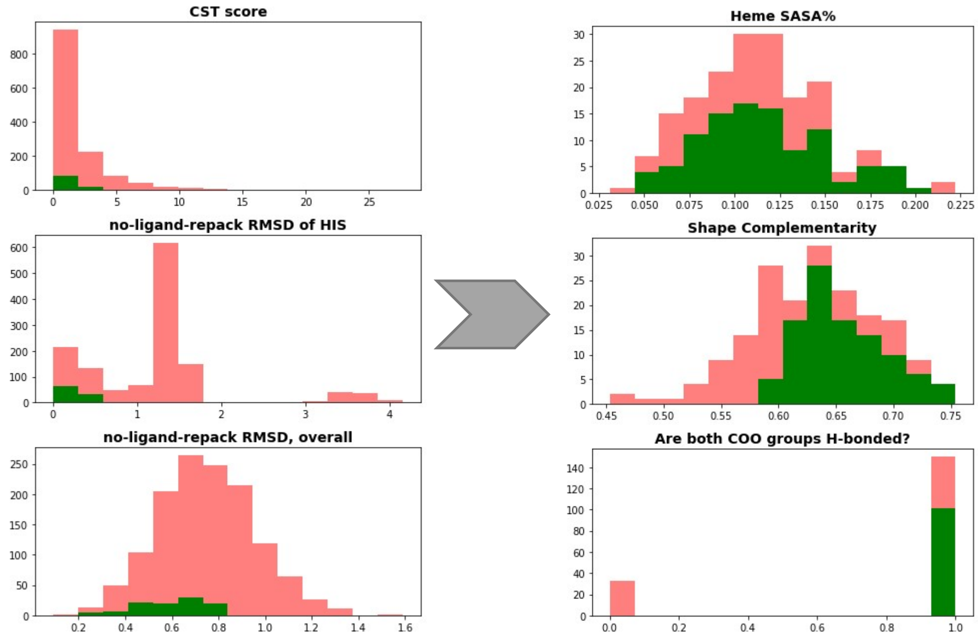

Supplementary Fig. 30. Distribution of metrics used for filtering designed heme binder models. Red – all scores, green – scores passing the filters. Three metrics in the last column were calculated only for the designs passing the first three metrics.

Ligand docking

Docking of heme into design models was performed using the Rosetta GALigandDock[16] mover and 'beta_genpot' scorefunction. The methyl-containing heme model was replaced with a model having an open coordination site, and 20 docking trajectories were seeded from the designed heme orientation. Each trajectory identified 20 best docks that were minimized and scored. Structures from all trajectories of a given design were combined and their Rosetta total score and ligand_rmsd (relative to the design model) values analyzed. A p_{near} score was calculated from the score-rmsd relationship to numerically describe how similar the lowest scoring docks are to the design model.[17] A p_{near} cutoff of 0.75 was used to filter most designs. The obtained forward docking funnels for each of the ordered designs are shown in Supplementary Fig. 31, together with the calculated p_{near} values.

A command that was used to run GALigandDock on a given design:

```
$ROSETTAPATH/main/source/bin/rosetta_scripts.default.linuxgccrelease -s holo.pdb -parser:protocol flexdock.xml -extra_res_fa /path/to/theozyme/HMM/genpot/HMM.params -beta -gen_potential
```

Where holo.pdb refers to a design model PDB file containing a ligand.

Contents of the ‘`flexdock.xml`’ file:

```
<ROSETTASCRIPTS>
  <SCOREFXNS>
    <ScoreFunction name="dockscore" weights="beta">
      <Reweight scoretype="gen_bonded" weight="1.0"/>
      <Reweight scoretype="fa_rep" weight="0.2"/>
      <Reweight scoretype="coordinate_constraint" weight="0.1"/>
    </ScoreFunction>
    <ScoreFunction name="relaxscore" weights="beta_cart"/>
    <ScoreFunction name="genpot" weights="beta">
      <Reweight scoretype="gen_bonded" weight="1.0"/>
    </ScoreFunction>
  </SCOREFXNS>

  <MOVERS>
    <GALigandDock    name="dock"    runmode="dockflex"    scorefxn="dockscore"    scorefxn_relax="relaxscore"
nativepdb="holo.pdb"  sidechains="aniso"  cartmin_lig="False"  premin_ligand="False"  final_exact_minimize="sc"
nreport="20" nrelax="20" >
                <Stage npool="20" repeats="5" />
    </GALigandDock>
  </MOVERS>

  <PROTOCOLS>
    <Add mover="dock" />
  </PROTOCOLS>
  <OUTPUT scorefxn="genpot" />
</ROSETTASCRIPTS>
```

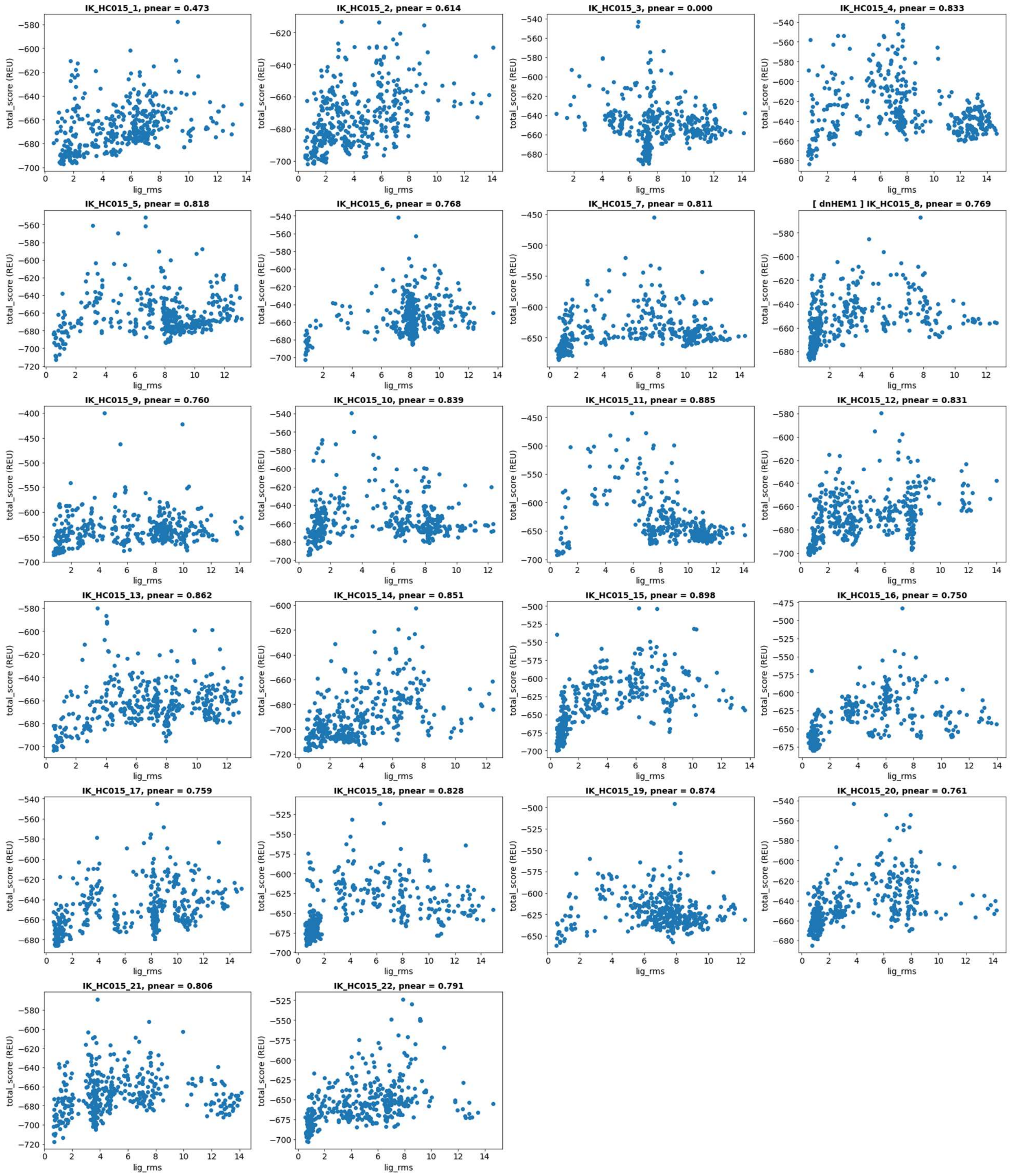

Supplementary Fig. 31. Docking funnels of each of the tested 22 designs showing ligand rmsd vs Rosetta score, obtained with GALigandDock.

3.2. DFT optimization of transition states

All calculations were performed using Gaussian 16 software.[18] Structural optimizations and frequency calculations were performed with B3LYP-D3 method along with 6-31G(d) basis set and the SDD ECP on Fe atom. Single point energy calculations were performed with M06L, M06 and B3LYP-D3 methods and def2-TZVP basis set. D3 dispersion correction was applied using the Becke-Johnson damping function.[19] Solvent effects of water and diethyl ether were included using the CPCM solvation model during optimization and single point energy calculations. This method has previously been shown to be appropriate for modeling similar systems.[20] Frequency calculations were performed to confirm whether the structure is a minimum or a transition state. Intrinsic reaction coordinate (IRC) analysis was used to confirm that the obtained transition states connect the correct minima.

Transition states leading to the formation of the *R,R* and *S,S* enantiomers of ethyl 2-phenyl-cycloclopropanecarboxylate were located in five different conformations resulting from the rotation around the Fe-C bond (Supplementary Fig. 32). The charge of the system was kept at -2 and the singlet electron configuration was considered. Imidazole was coordinated to the proximal site of heme to mimic histidine coordination. To verify that all of these transition states are energetically relevant their single point energies were calculated with multiple DFT functionals and the resulting corrected free energies compared (Supplementary Table 8). Implicit solvation by water and diethyl ether were used to mimic aqueous and protein pocket environments (dielectric constant of diethyl ether has been reported to approximate that of a protein pocket[21]). As judged by the relative free energies at all tested levels of theory, all of the conformers are energetically feasible. In particular, when considering potential perturbations to the energies in a specific protein pocket environment.

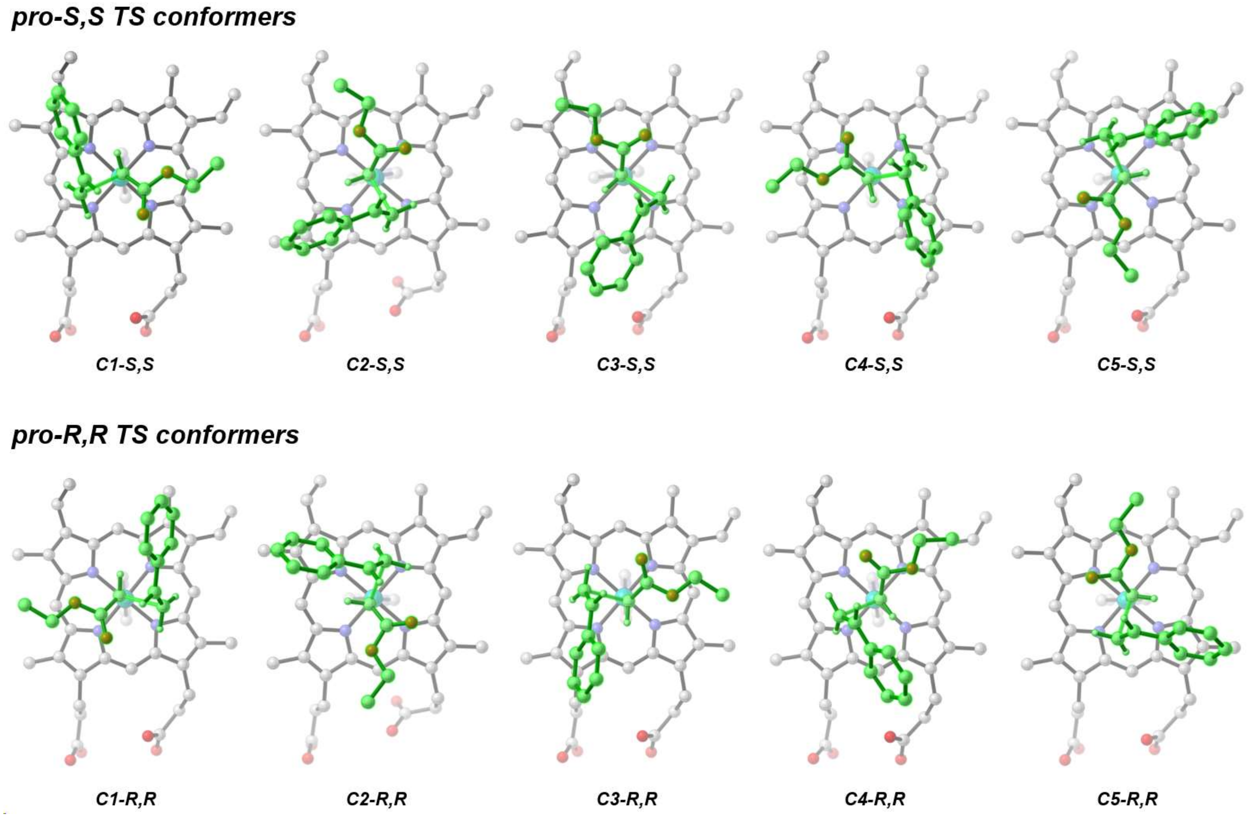

Supplementary Fig. 32. Conformations of the pro-*S,S* and pro-*R,R* cyclopropanation transition states.

Supplementary Table 8. Relative activation free energies of pro-*S*,*S* and pro-*R*,*R* transition state conformers (in kcal/mol), obtained by using different DFT methods and implicit solvents. Bold = lowest, underlined = highest energy conformer.

Conformer	*CPCM solvent*	*H_2O*	*H_2O*	*H_2O*	*Et_2O*	*Et_2O*	*Et_2O*
	Method	**M06L**	**M06**	**B3LYP-D3**	**M06L**	**M06**	**B3LYP-D3**
**C1-*S,S***		**0.0**	**0.0**	**0.0**	0.4	0.5	0.7
**C2-*S,S***		2.2	1.3	1.2	0.8	**0.0**	**0.0**
**C3-*S,S***		3.6	3.3	3.1	2.0	1.7	1.6
**C4-*S,S***		0.3	0.3	0.1	**0.0**	**0.0**	**0.0**
**C5-*S,S***		2.0	2.1	1.9	1.0	1.2	1.1
**C1-*R,R***		0.2	**0.0**	0.5	1.4	1.8	1.8
**C2-*R,R***		1.1	0.4	1.0	**0.0**	**0.0**	**0.0**
**C3-*R,R***		**0.0**	**0.0**	**0.0**	0.1	0.7	0.1
**C4-*R,R***		3.4	3.1	2.8	2.5	2.7	1.9
**C5-*R,R***		0.7	0.7	1.5	1.0	1.8	2.0

Rotamer library creation

Conformational diversity of each of these transition states was further increased by sampling the rotamers of the flexible propionic acid groups of heme, as well as the conformers of the vinyl groups. We aimed to find various low energy conformers that aren't necessarily exactly representing possible lowest energy local minima but are still sufficiently close to them. To achieve that, dihedral angles were randomly sampled for the 8 rotable bonds corresponding to the two propionic acid and two vinyl groups. To ensure conformational diversity, all saved conformers have at least one of the dihedral angles at least ±20° different from any other structure. Lastly, the conformers were sampled as frozen coordinates with no geometry optimization, in order not to affect the transition state geometries. Energies of the conformers were evaluated using the GFN2-XTB semiempirical QM method.[22] This procedure was performed using a Python script that packages together conformer sampling, energy evaluation and analysis, and is available on GitHub.[23] 100 conformers were saved for each transition state rotamer by sampling 2000 random configurations.

Rosetta params file creation

The generated conformers were initially saved as XYZ files that were subsequently converted to MOLfiles using OpenBabel.[24] The bonding information in the MOLfile was manually inspected to ensure that the entire structure is represented as a single fragment, and edited, if necessary. Thereafter, *mol2params.py* script, available within Rosetta, was used to convert the MOLfile to a Rosetta-compatible *.params* file. The partial charges of the carboxylate oxygen atoms of the propionate groups were adjusted in the params files from -0.74 to -1.24 to increase the likelihood of H-bonds being created with these atoms during Rosetta design. The conformers described above were included in the params file via an accompanying PDB file. All created params files are available for download at GitHub.[14]

3.3. Carbene transferase active site design

The transition state models of *S*,*S* and *R*,*R* addition styrene to iron carbenoid, the Rosetta params files and the accompanying rotamer libraries were created following the procedures described in section 3.2. The imidazole ligand was removed before creating the params file. The corresponding theozyme models HSS and HRR are available on GitHub.[14]

Transition state models were aligned to the heme molecule in the dnHEM1 design model based on the positions of the corresponding porphyrin nitrogen atoms. A subset of designs used, as the starting point, a structural model obtained by predicting the structure of dnHEM1 from single sequence with AlphaFold2[25] model 4, and relaxing it with FastRelax together with heme. Design was performed using the Rosetta FastDesign[26] mover and 'beta_nov_16' scorefunction, and implemented through a PyRosetta script '`replace_HMM_HXX_design.py`' available for download on GitHub.[14] A constraint defining the interaction geometry between His148 and the heme model was applied during design using the Rosetta 'AddOrRemoveMatchCsts' mover. Design trajectories were seeded by selecting a random ligand rotamer in each iteration. Positions 5, 7, 8, 11, 12, 39, 42, 43, 46, 74, 75, 78, 109, 183 were set to be designable (Supplementary Fig. 33), and other positions within 12Å of any ligand heavy atom were allowed to be repacked. The designed models were scored by metrics describing the His-Fe interaction geometry, shape-complementarity, and preorganization of the heme binding pocket (assessed by side chain packing calculations in the absence of the heme), with the cutoff criteria and distributions of scores depicted in Supplementary Table 7 and Supplementary Fig. 34, respectively.

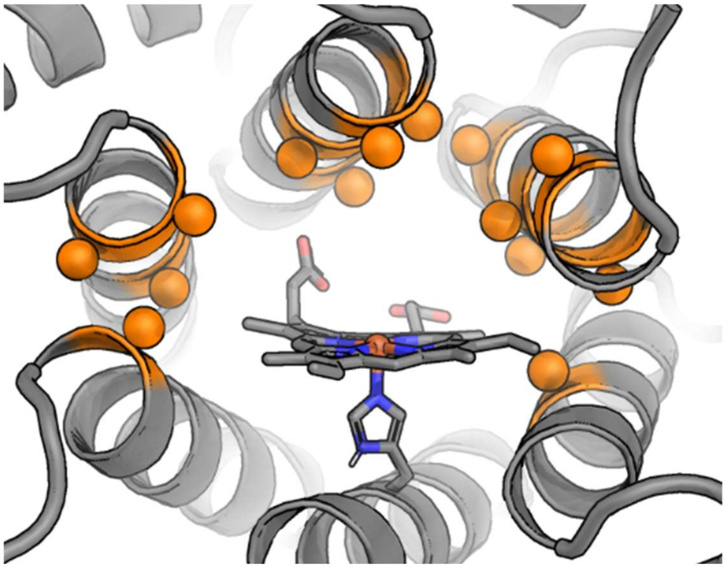

Supplementary Fig. 33. Positions in the heme binding pocket that were considered for redesigning for the olefin cyclopropanation active site, indicated as orange spheres.

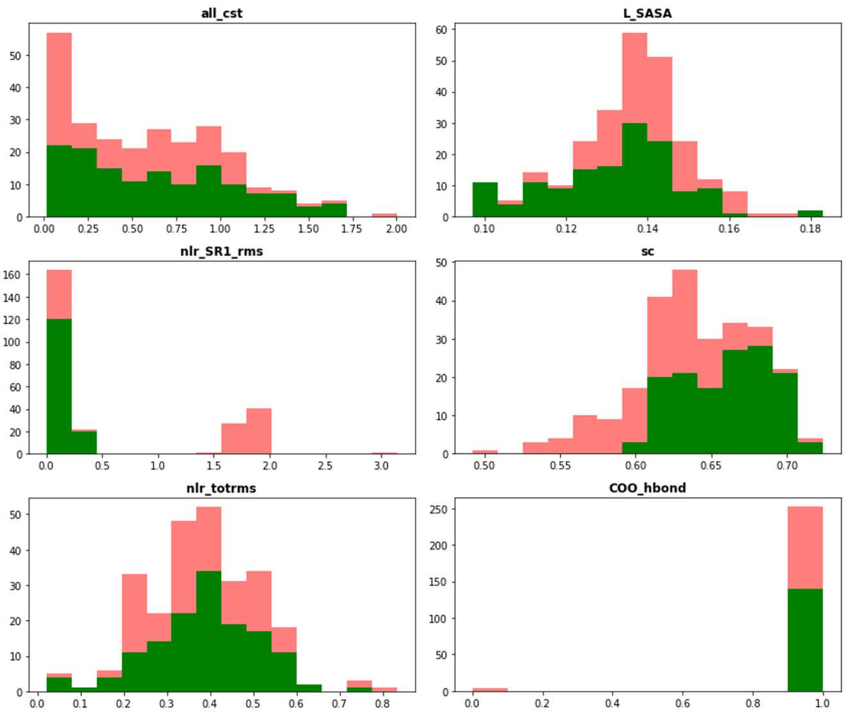

Supplementary Fig. 34. Distribution of metrics used for filtering designed carbene transferase models. Red – all scores, green – scores passing the filters.

Successful designs were relaxed with the transition state model of the original, as well as the opposite enantiomer using the FastRelax mover (Supplementary Fig. 25). 45 separate relax trajectories were seeded from random theozyme conformations, together with randomized pocket residue rotamers. Designs yielding both lower total score and dd$G$ metric with the original enantiomer TS, as well as maintaining the designed TS conformation (based on rotation around the Fe-C bond) were considered for ordering (Supplementary Fig. 36). The ligand replacement, relaxing and scoring have been implemented in a Python script '`replace_HSS_HRR_relax.py`' available for download on GitHub.[14] Lastly, surface mutations, mostly the same as those used for dnHEM1_pI6, were introduced to the successful designs in order to lower the pI of the protein to 5.5-6.

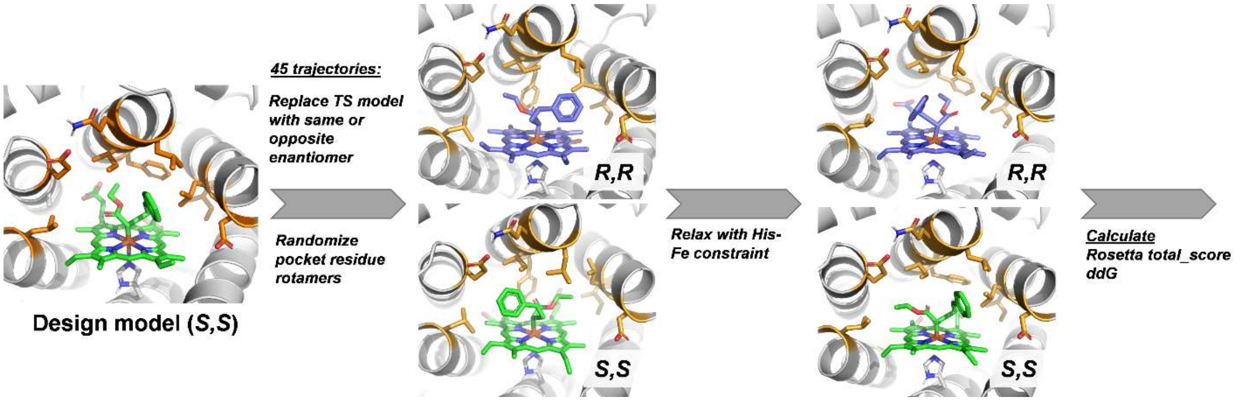

Supplementary Fig. 35. Workflow for olefin cyclopropanation enantioselectivity analysis of dnHEM1 redesigns with Rosetta. Successful designs must have both Rosetta total score and dd$G$ values lower for the enantiomer present in the design model.

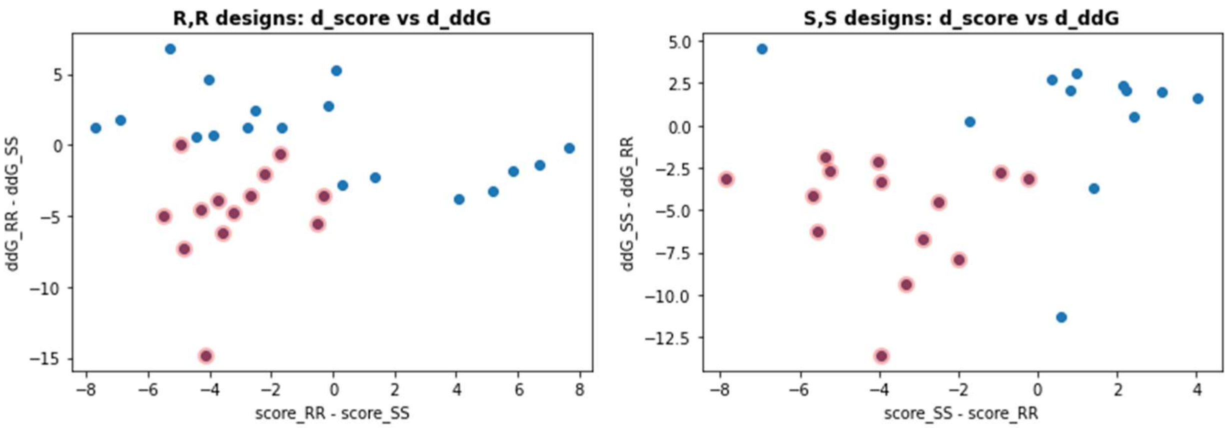

Supplementary Fig. 36. Distribution of Rosetta score and dd*G* differences between the designed enantiomer and the opposite enantiomer. Datapoints corresponding to designs selected for experimental testing are highlighted in red.

3.4. Energetic and thermal data for computed structures

XYZ coordinates of computed structures are available for download as a separate multiXYZ file. Raw Gaussian output files are available for download on GitHub.[14]

C1-*S,S*

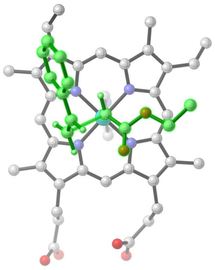

```
Zero-point correction=                           0.879440 (Hartree/Particle)
 Thermal correction to Energy=                    0.938962
 Thermal correction to Enthalpy=                  0.939906
 Thermal correction to Gibbs Free Energy=         0.781047
 Sum of electronic and zero-point Energies=          -2799.591067
 Sum of electronic and thermal Energies=             -2799.531545
 Sum of electronic and thermal Enthalpies=           -2799.530601
 Sum of electronic and thermal Free Energies=        -2799.689460

CPCM (H2O) M06L/def2TZVP E = -3940.696132
CPCM (H2O) M06/def2TZVP E = -3939.12817868
CPCM (H2O) B3LYP/def2TZVP E = -3941.38621554
CPCM (Et2O) M06L/def2TZVP E = -3940.62228403
CPCM (Et2O) M06/def2TZVP E = -3939.05299838
CPCM (Et2O) B3LYP/def2TZVP E = -3941.31164525
```

C2-*S,S*

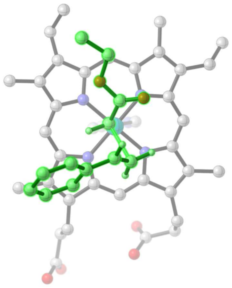

```
Zero-point correction=                           0.880396 (Hartree/Particle)
 Thermal correction to Energy=                    0.939704
 Thermal correction to Enthalpy=                  0.940649
 Thermal correction to Gibbs Free Energy=         0.782273
 Sum of electronic and zero-point Energies=          -2799.589584
 Sum of electronic and thermal Energies=             -2799.530275
 Sum of electronic and thermal Enthalpies=           -2799.529331
 Sum of electronic and thermal Free Energies=        -2799.687706

CPCM (H2O) M06L/def2TZVP E = -3940.69386044
CPCM (H2O) M06/def2TZVP E = -3939.12733989
CPCM (H2O) B3LYP/def2TZVP E = -3941.38555322
CPCM (Et2O) M06L/def2TZVP E = -3940.62280683
CPCM (Et2O) M06/def2TZVP E = -3939.05491197
CPCM (Et2O) B3LYP/def2TZVP E = -3941.31393049
```

C3-*S,S*

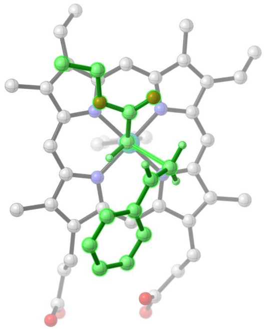

```
Zero-point correction=                           0.879740 (Hartree/Particle)
 Thermal correction to Energy=                    0.939235
 Thermal correction to Enthalpy=                  0.940179
 Thermal correction to Gibbs Free Energy=         0.781513
 Sum of electronic and zero-point Energies=          -2799.587800
 Sum of electronic and thermal Energies=             -2799.528305
 Sum of electronic and thermal Enthalpies=           -2799.527361
 Sum of electronic and thermal Free Energies=        -2799.686027

CPCM (H2O) M06L/def2TZVP E = -3940.69088687
CPCM (H2O) M06/def2TZVP E = -3939.12340748
CPCM (H2O) B3LYP/def2TZVP E = -3941.38179208
CPCM (Et2O) M06L/def2TZVP E = -3940.62020639
CPCM (Et2O) M06/def2TZVP E = -3939.05146322
CPCM (Et2O) B3LYP/def2TZVP E = -3941.31059534
```

C4-*S,S*

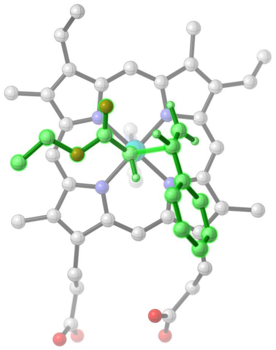

```
Zero-point correction=                           0.879626 (Hartree/Particle)
 Thermal correction to Energy=                    0.939112
 Thermal correction to Enthalpy=                  0.940057
 Thermal correction to Gibbs Free Energy=         0.781509
 Sum of electronic and zero-point Energies=          -2799.590613
 Sum of electronic and thermal Energies=             -2799.531126
 Sum of electronic and thermal Enthalpies=           -2799.530182
 Sum of electronic and thermal Free Energies=        -2799.688729

CPCM (H2O) M06L/def2TZVP E = -3940.69613222
CPCM (H2O) M06/def2TZVP E = -3939.12822735
CPCM (H2O) B3LYP/def2TZVP E = -3941.38647608
CPCM (Et2O) M06L/def2TZVP E = -3940.62335264
CPCM (Et2O) M06/def2TZVP E = -3939.05421718
CPCM (Et2O) B3LYP/def2TZVP E = -3941.31311131
```

C5-*S,S*

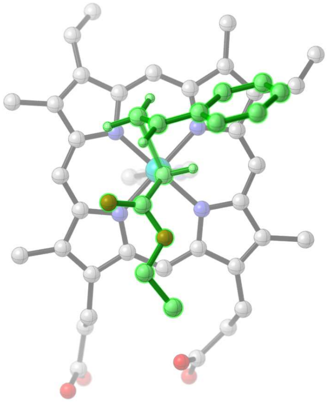

```
Zero-point correction=                           0.880038 (Hartree/Particle)
 Thermal correction to Energy=                    0.939310
 Thermal correction to Enthalpy=                  0.940254
 Thermal correction to Gibbs Free Energy=         0.783211
 Sum of electronic and zero-point Energies=          -2799.590280
 Sum of electronic and thermal Energies=             -2799.531008
 Sum of electronic and thermal Enthalpies=           -2799.530064
 Sum of electronic and thermal Free Energies=        -2799.687108

CPCM (H2O) M06L/def2TZVP E = -3940.69512387
CPCM (H2O) M06/def2TZVP E = -3939.12694248
CPCM (H2O) B3LYP/def2TZVP E = -3941.38539755
CPCM (Et2O) M06L/def2TZVP E = -3940.62349726
CPCM (Et2O) M06/def2TZVP E = -3939.05403296
CPCM (Et2O) B3LYP/def2TZVP E = -3941.31316674
```

C1-*R,R*

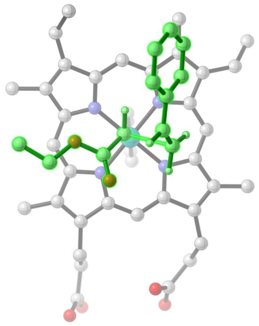

```
Zero-point correction=                           0.879542 (Hartree/Particle)
 Thermal correction to Energy=                    0.939068
 Thermal correction to Enthalpy=                  0.940012
 Thermal correction to Gibbs Free Energy=         0.780879
 Sum of electronic and zero-point Energies=          -2799.590177
 Sum of electronic and thermal Energies=             -2799.530650
 Sum of electronic and thermal Enthalpies=           -2799.529706
 Sum of electronic and thermal Free Energies=        -2799.688839

CPCM (H2O) M06L/def2TZVP E = -3940.69507333
CPCM (H2O) M06/def2TZVP E = -3939.12742671
CPCM (H2O) B3LYP/def2TZVP E = -3941.38575752
CPCM (Et2O) M06L/def2TZVP E = -3940.62116766
CPCM (Et2O) M06/def2TZVP E = -3939.0522336
CPCM (Et2O) B3LYP/def2TZVP E = -3941.31116291
```

C2-*R,R*

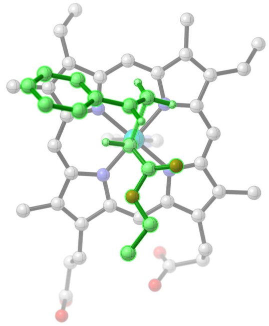

```
Zero-point correction=                           0.880262 (Hartree/Particle)
 Thermal correction to Energy=                    0.939522
 Thermal correction to Enthalpy=                  0.940467
 Thermal correction to Gibbs Free Energy=         0.782685
 Sum of electronic and zero-point Energies=          -2799.590648
 Sum of electronic and thermal Energies=             -2799.531388
 Sum of electronic and thermal Enthalpies=           -2799.530443
 Sum of electronic and thermal Free Energies=        -2799.688225

CPCM (H2O) M06L/def2TZVP E = -3940.69550877
CPCM (H2O) M06/def2TZVP E = -3939.12866269
CPCM (H2O) B3LYP/def2TZVP E = -3941.38674409
CPCM (Et2O) M06L/def2TZVP E = -3940.62513829
CPCM (Et2O) M06/def2TZVP E = -3939.05695099
CPCM (Et2O) B3LYP/def2TZVP E = -3941.31582537
```

C3-*R,R*

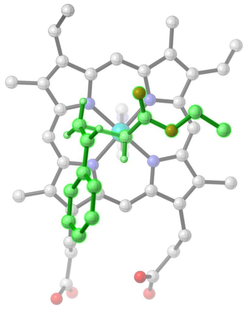

```
Zero-point correction=                           0.879384 (Hartree/Particle)
 Thermal correction to Energy=                    0.939009
 Thermal correction to Enthalpy=                  0.939953
 Thermal correction to Gibbs Free Energy=         0.780403
 Sum of electronic and zero-point Energies=          -2799.590602
 Sum of electronic and thermal Energies=             -2799.530977
 Sum of electronic and thermal Enthalpies=           -2799.530033
 Sum of electronic and thermal Free Energies=        -2799.689583

CPCM (H2O) M06L/def2TZVP E = -3940.69492505
CPCM (H2O) M06/def2TZVP E = -3939.12693724
CPCM (H2O) B3LYP/def2TZVP E = -3941.38603336
CPCM (Et2O) M06L/def2TZVP E = -3940.6227326
CPCM (Et2O) M06/def2TZVP E = -3939.05356303
CPCM (Et2O) B3LYP/def2TZVP E = -3941.31334333
```

C4-*R,R*

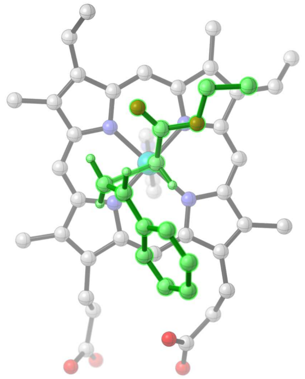

```
Zero-point correction=                           0.879834 (Hartree/Particle)
 Thermal correction to Energy=                    0.939316
 Thermal correction to Enthalpy=                  0.940260
 Thermal correction to Gibbs Free Energy=         0.781807
 Sum of electronic and zero-point Energies=          -2799.589066
 Sum of electronic and thermal Energies=             -2799.529583
 Sum of electronic and thermal Enthalpies=           -2799.528639
 Sum of electronic and thermal Free Energies=        -2799.687092

CPCM (H2O) M06L/def2TZVP E = -3940.69083322
CPCM (H2O) M06/def2TZVP E = -3939.12349142
CPCM (H2O) B3LYP/def2TZVP E = -3941.38298806
CPCM (Et2O) M06L/def2TZVP E = -3940.62025292
CPCM (Et2O) M06/def2TZVP E = -3939.05171129
CPCM (Et2O) B3LYP/def2TZVP E = -3941.31190005
```

C5-*R,R*

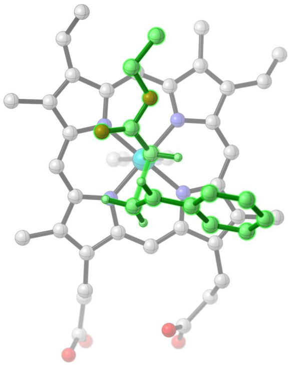

```
Zero-point correction=                           0.879929 (Hartree/Particle)
 Thermal correction to Energy=                    0.939204
 Thermal correction to Enthalpy=                  0.940149
 Thermal correction to Gibbs Free Energy=         0.782791
 Sum of electronic and zero-point Energies=          -2799.590154
 Sum of electronic and thermal Energies=             -2799.530879
 Sum of electronic and thermal Enthalpies=           -2799.529935
 Sum of electronic and thermal Free Energies=        -2799.687292

CPCM (H2O) M06L/def2TZVP E = -3940.69618627
CPCM (H2O) M06/def2TZVP E = -3939.12816708
CPCM (H2O) B3LYP/def2TZVP E = -3941.38599526
CPCM (Et2O) M06L/def2TZVP E = -3940.62363561
CPCM (Et2O) M06/def2TZVP E = -3939.05421459
CPCM (Et2O) B3LYP/def2TZVP E = -3941.31270298
```

4. References

1. Dang, B. *et al.* SNAC-tag for sequence-specific chemical protein cleavage. *Nat. Methods* **16**, 319–322 (2019).
2. Gibson, D. G. *et al.* Enzymatic assembly of DNA molecules up to several hundred kilobases. *Nat. Methods* **6**, 343–345 (2009).
3. Wicky, B. I. M. *et al.* Hallucinating symmetric protein assemblies. *Science* **378**, 56–61 (2022).
4. Berry, E. A. & Trumpower, B. L. Simultaneous determination of hemes a, b, and c from pyridine hemochrome spectra. *Analytical Biochemistry* (1987) doi:10.1016/0003-2697(87)90643-9.
5. Ennist, N. M., Stayrook, S. E., Dutton, P. L. & Moser, C. C. Rational design of photosynthetic reaction center protein maquettes. *Front Mol Biosci* **9**, 997295 (2022).
6. Gasteiger, E. *et al.* Protein Identification and Analysis Tools on the ExPASy Server. in *The Proteomics Protocols Handbook* (ed. Walker, J. M.) 571–607 (Humana Press, 2005).
7. Pott, M. *et al.* A Noncanonical Proximal Heme Ligand Affords an Efficient Peroxidase in a Globin Fold. *J. Am. Chem. Soc.* **140**, 1535–1543 (2018).
8. Bordeaux, M., Tyagi, V. & Fasan, R. Highly diastereoselective and enantioselective olefin cyclopropanation using engineered myoglobin-based catalysts. *Angew. Chem. Int. Ed Engl.* **54**, 1744–1748 (2015).
9. Joosten, R. P., Long, F., Murshudov, G. N. & Perrakis, A. The PDB-REDO server for macromolecular structure model optimization. *IUCrJ* **1**, 213–220 (2014).
10. McCoy, A. J. *et al.* Phaser crystallographic software. *Journal of Applied Crystallography* **40**, 658–674 (2007).
11. Doyle, L. *et al.* Rational design of α-helical tandem repeat proteins with closed architectures. *Nature* **528**, 585–588 (2015).
12. Adams, P. D. *et al.* PHENIX: A comprehensive Python-based system for macromolecular structure solution. *Acta Crystallographica Section D: Biological Crystallography* (2010) doi:10.1107/S0907444909052925.

13. Zanghellini, A. *et al.* New algorithms and an in silico benchmark for computational enzyme design. *Protein Sci.* **15**, 2785–2794 (2006).

14. Kalvet, I. De novo heme binder design repository. https://github.com/ikalvet/heme_binder_design.git.

15. Richter, F., Leaver-Fay, A., Khare, S. D., Bjelic, S. & Baker, D. De novo enzyme design using Rosetta3. *PLoS One* **6**, e19230 (2011).

16. Park, H., Zhou, G., Baek, M., Baker, D. & DiMaio, F. Force Field Optimization Guided by Small Molecule Crystal Lattice Data Enables Consistent Sub-Angstrom Protein-Ligand Docking. *J. Chem. Theory Comput.* **17**, 2000–2010 (2021).

17. Bhardwaj, G. *et al.* Accurate de novo design of hyperstable constrained peptides. *Nature* **538**, 329–335 (2016).

18. Frisch, M. J. *et al. Gaussian 16 Rev. C.01*. (2016).

19. Grimme, S., Ehrlich, S. & Goerigk, L. Effect of the damping function in dispersion corrected density functional theory. *J. Comput. Chem.* **32**, 1456–1465 (2011).

20. Lewis, R. D. *et al.* Catalytic iron-carbene intermediate revealed in a cytochrome *c* carbene transferase. *Proc. Natl. Acad. Sci. U. S. A.* **115**, 7308–7313 (2018).

21. Li, B. *et al.* Mechanism of the Stereoselective Catalysis of Diels-Alderase PyrE3 Involved in Pyrroindomycin Biosynthesis. *J. Am. Chem. Soc.* **144**, 5099–5107 (2022).

22. Bannwarth, C. *et al.* Extended tight - binding quantum chemistry methods. *Wiley Interdiscip. Rev. Comput. Mol. Sci.* **11**, (2021).

23. Kalvet, I. frozen-conf-xtb: A python script for performing frozen coordinate conformational searches using GFN2-XTB energies. https://github.com/ikalvet/frozen-conf-xtb.git.

24. O'Boyle, N. M. *et al.* Open Babel: An open chemical toolbox. *J. Cheminform.* **3**, 33 (2011).

25. Jumper, J. *et al.* Highly accurate protein structure prediction with AlphaFold. *Nature* **596**, 583–589 (2021).

26. Maguire, J. B. *et al.* Perturbing the energy landscape for improved packing during computational protein design. *Proteins* **89**, 436–449 (2021).

27. Dauparas, J. *et al.* Robust deep learning-based protein sequence design using ProteinMPNN. *Science* **378**, 49–56 (2022).
